# Insights into the transcriptomic response of the plant engineering bacterium *Ensifer adhaerens* OV14 during transformation

**DOI:** 10.1038/s41598-019-44648-8

**Published:** 2019-07-17

**Authors:** Evelyn Zuniga-Soto, David A. Fitzpatrick, Fiona M. Doohan, Ewen Mullins

**Affiliations:** 1 Department of Crop Science, Teagasc Crops Research Centre, Oak Park, Carlow, Ireland; 2 Department of Biology, National University of Ireland Maynooth, Maynooth, Ireland; 3 School of Biology and Environmental Sciences, University College Dublin, Belfield, Dublin 4 Ireland

**Keywords:** Molecular engineering in plants, Plant biotechnology

## Abstract

The ability to engineer plant genomes has been primarily driven by the soil bacterium *Agrobacterium tumefaciens* but recently the potential of alternative rhizobia such as *Rhizobium etli* and *Ensifer adhaerens* OV14, the latter of which supports *Ensifer* Mediated Transformation (EMT) has been reported. Surprisingly, a knowledge deficit exists in regards to understanding the whole genome processes underway in plant transforming bacteria, irrespective of the species. To begin to address the issue, we undertook a temporal RNAseq-based profiling study of *E*. *adhaerens* OV14 in the presence/absence of *Arabidopsis thaliana* tissues. Following co-cultivation with root tissues, 2333 differentially expressed genes (DEGs) were noted. Meta-analysis of the RNAseq data sets identified a clear shift from plasmid-derived gene expression to chromosomal-based transcription within the early stages of bacterium-plant co-cultivation. During this time, the number of differentially expressed prokaryotic genes increased steadily out to 7 days co-cultivation, a time at which optimum rates of transformation were observed. Gene ontology evaluations indicated a role for both chromosomal and plasmid-based gene families linked specifically with quorum sensing, flagellin production and biofilm formation in the process of EMT. Transcriptional evaluation of *vir* genes, housed on the pCAMBIA 5105 plasmid in *E*. *adhaerens* OV14 confirmed the ability of *E*. *adhaerens* OV14 to perceive and activate its transcriptome in response to the presence of 200 µM of acetosyringone. Significantly, this is the first study to characterise the whole transcriptomic response of a plant engineering bacterium in the presence of plant tissues and provides a novel insight into prokaryotic genetic processes that support T-DNA transfer.

## Introduction

The soil-borne plant pathogen *Agrobacterium tumefaciens* underpins *Agrobacterium*-mediated transformation (AMT) as the primary platform for the generation of engineered crop varieties, which cover ~189.8 million hectares globally^1^ (ISAAA, 2018). Achieved via the transfer of the T-DNA section of *A*. *tumefaciens*’ Ti plasmid into a targeted plant cell, the suitability of non-*Agrobacterium* strains to facilitate this process of horizontal gene transfer was first described by^2^ in 1997. Based on investigations around the potential of non-*Agrobacterium* strains to overcome some of the technical limitations (e.g. genotype dependency) of AMT, previous studies have examined *Rhizobium sp*. NGR234, *Sinorhizobium meliloti* and *Mesorhizobium loti*, collectively termed Transbacter™ ^3^. Evidence of T-DNA transfer into plant cells was shown for all three-bacterial species, but transformation efficiencies were considered too low for mainstream applications^4^. The non-pathogenic bacterium *Ensifer adhaerens* OV14 has been used in *Ensifer*-Mediated Transformation (EMT) to transfer DNA into rice^5^, canola^6^, cassava^7^ and potato^8^. Recently, *Rhizobium etli*^9^ and *Ochrobactrum haywardense*^10^ have also been added to the list of non-*Agrobacterium* spp capable of transforming plant cells. A complementary exploration of the transcriptional activation of *vir* genes contained on the *R*. *etli* p42a has provided further insight into similar and distinctive features compared to their *A*. *tumefaciens* counterpart^11^.

At a structural level, whole genome comparisons have identified key differences between *A*. *tumefaciens* and some of the non-*Agrobacterium spp*. For example, a comparative analysis of the 7.7 Mb genome of *E*. *adhaerens* OV14 against *A*. *tumefaciens* C58 (5.67 Mb) and the symbiont *Sinorhizobium meliloti* 1021 (6.7 Mb), revealed that *E*. *adhaerens* OV14 is equipped with homologs to several chromosomal-based genes, which are known to be essential for AMT. Of significance, genes that are deemed non-essential but exert a positive influence on the ability to transform a plant genome, while absent from the *S*. *meliloti* strain 1021 genome were found present in the genome of *E*. *adhaerens* OV14^12^. The same study noted that phylogenetically *E*. *adhaerens* OV14 and *A*. *tumefaciens* C58 reside in separate superclades of the Rhizobiacae family, which has been recently confirmed^13^. A syntenic analysis of the three studied bacteria revealed that *E*. *adhaerens* OV14 is void of functional *vir* gene orthologs across its four replicons. Yet, when *E*. *adhaerens* OV14 carries a Ti equivalent-plasmid (e.g. pCAMBIA 5105) containing a complement of *vir* genes it acquires the ability to accommodate, transfer and successfully integrate stable copies of T-DNA. The processes supporting this remain unknown, as indeed does the rate of *vir* gene transcription relative to whole genome activity and the role (if any) of individual *E*. *adhaerens* OV14 replicons in supporting EMT.

While multiple studies have investigated the processes that directly support the transfer and stable integration of T-DNA into host chromatin^14–23^, a complete understanding of the eukaryotic and indeed the complementary prokaryotic networks that support intranuclear targeting has yet to be fully attained^22^. To date, gene expression studies conducted to better understand the functional transformation machinery within *Agrobacterium* and non-*Agrobacterium* species have been restricted to a limited number of genes^11,17,24–27^, or oriented towards the plant’s response in the presence of the bacterium^28–32^. For example, the importance of the plant phenolic acetosyringone in eliciting the induction of virulence genes within *A*. *tumefaciens* is well understood^24,25^, while chromosomal genes such as *att*, *exoC*, *chvA*, *chvD*, *chvB*, and *chvE* are known to support processes central to transformation such as bacteria-plant cell attachment, exopolysaccharide production, secretion and sugar transportation^18^. Yet, in spite of the relevance of genetic engineering to global crop production, little else has been achieved in regards to characterizing whole transcriptional activity in plant transforming bacteria during the transformation process itself.

In response, here we report the first transcriptome analysis of a bacterium with the capacity for plant transformation. By surveying the response of the core *E*. *adhaerens* OV14 transcriptome as well as the unitary plasmid pCAMBIA 5105 (henceforth pC5105) following plant root inoculation, the temporal expression patterns of 25 vir genes (residing on pC5105) in parallel to the activity of the 7074 transcriptionally active sequences within the *E*. *adhaerens* OV14 genome were examined. The impact of this research is relevant to both the non-*Agrobacterium* and the *Agrobacterium* research communities interested in the molecular mechanisms supporting bacterial-mediated transformation of plant cells and provides a baseline dataset from which further functionality studies can now be completed.

## Results

### Rate and degree of EMT infection in *A*. *thaliana* roots

**Figure 1:**
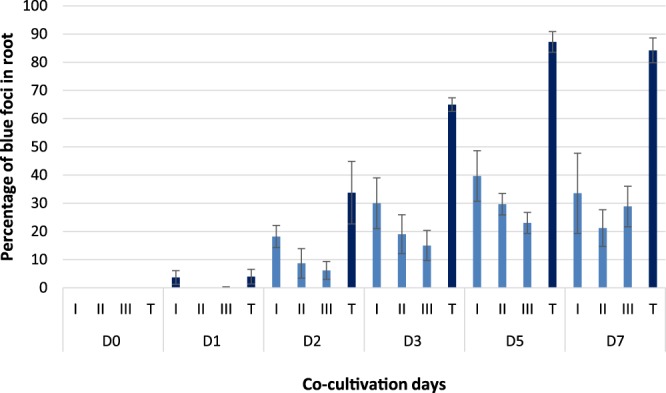
Progression of transient T-DNA transfer in *A*. *thaliana* roots co-cultivated with EOV14_5105 in a 7-day time course as measured via histochemical GUS staining. Incidence of GUS transient staining measured as percentage of blue foci ranked as: roots with a single spot (Class I), roots with <50% stained (Class II); roots with >50% stained (Class III) and total number of roots stained (T). D0, D1, D2, D3, D5, D7 indicate the days after co-cultivation. Error bars indicate standard error from three biological replicates.

Optimum transient T-DNA transfer (87.2%, Fig. 1) was observed five days after inoculation with *E. adhaerens* OV14 containing pC5105 (EOV14_5105). Recorded as the percentage of blue foci visible on treated *A*. *thaliana* roots, the first signs of transient transformation were noted by D1 (3.9%), which increased significantly (P < 0.05) through to D3 (33.7%) and D5 (87.2%), decreasing slightly to 84.2% at 7 days post-treatment (Fig. 1). In regards to the degree of staining on treated roots (class I, II or III) class I was the highest recorded for each time point and peaked at D5. Class II staining was consistently lower in occurrence compared to class I but was also optimum at D5, while class III was the lowest recorded form of staining.

### Transcriptomic activity in EOV14_5105 following co-cultivation with *A*. *thaliana* roots

**Figure 2:**
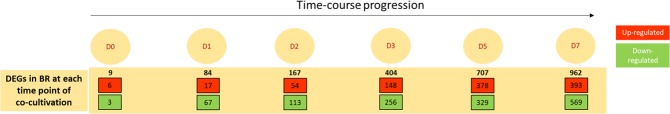
Schematic representation detailing the relative number of differentially expressed genes (DEGs) identified within EOV14_5105 following exposure to *A*. *thaliana* root tissues (BR) at 0, 1, 2, 3, 5, and 7 days (D0, D1, D2, D3, D5, D7) after inoculation. Details of the identification of these genes are depicted in Supplementary Fig. S3.

Thirty-six sequencing libraries/samples were created with raw reads ranging from 10 to 12 million per sample and a total of 406.9 million reads were recorded across the experiment. An average of 97.1% of reads were successfully mapped against the reference genome of EOV14_5105 plus the pC5105 plasmid (Supplementary Table S1). A total of 7074 and 29 genes were identified for EOV14_5105 and the plasmid pC5105 respectively, with 3756 genes on chromosome 1, 1774 on chromosome 2, 1441 associated with pOV14b and 103 with pOV14c. A total of 2333 DEGs were identified in the present study. After extracting the DEGs at each specific timepoint, 9 were identified at D0, 84 by D1, 167 by D2, 404 by D3, 707 by D5 and 962 by D7 (Fig. 2), with a greater overall proportion of down-regulated compared to up-regulated genes. The highest fold increase (9.3-fold) in the number of DEGs (84 at D1 v. 9 at D2) was observed in the transition from D0 to D1 (Fig. 2), with the increase in the subsequent time points ranging from 1.5- to 2.5-fold.

**Figure 3:**
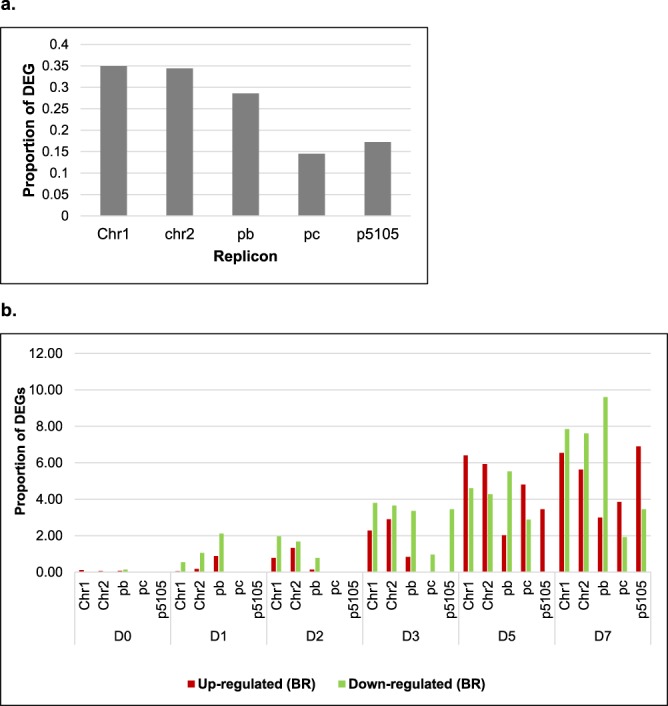
Relative distribution of DEGs in BR located across four replicons in *E*. *adhaerens* and the plasmid pCAMBIA5105. The DEGs from Fig. 2 sorted across the respective replicons from *E*. *adhaerens* and the plasmid pCAMBIA5105 (**a**). Detail of the number of DEGs found per replicon at each co-cultivation day (**b**). Proportions were calculated by dividing the number of DEGs for a specific replicon by the total number of genes for that replicon. Notations for replicons in *E*. *adhaerens* OV14: Chromosome 1 (Chr1); chromosome 2, (Chr2); plasmid pOV14b (pb); plasmid pOV14c (pc). Plasmid pCAMBIA5105 (p5105). Co-cultivation days of EOV14_5105 following exposure to *A*. *thaliana* roots from day 0 (D0) day 1, (D1) day 2, (D2) day 3, (D3) day 5 (D5) and day 7 (D7).

In order to observe how the DEGs were distributed across replicons in *E*. *adhaerens* OV14 and the plasmid pCAMBIA5105, the genes observed in Fig. 2 were sorted according to their corresponding replicon. The results indicated that the DEGs were represented across all four replicons, with the highest proportion of genes located on chromosome 1 and 2 followed by pOV14b (pb) and pOV14c (Fig. 3a). Approximately one fifth of DEGs recorded were associated with pCAMBIA5105 during the time course (Fig. 3a). Relative to the total number of genes on each respective replicon, the proportion of DEGs increased steadily through to D5–D7 (Fig. 3b). Meanwhile, during the first day of co-cultivation (D1) the proportion of DEGs in plasmid pOV14b (pb) was superior compared to the proportion of chromosomal-derived DEGs. This phenomenon was also observed in D5 and D7 for the proportion of down-regulated genes.

**Figure 4:**
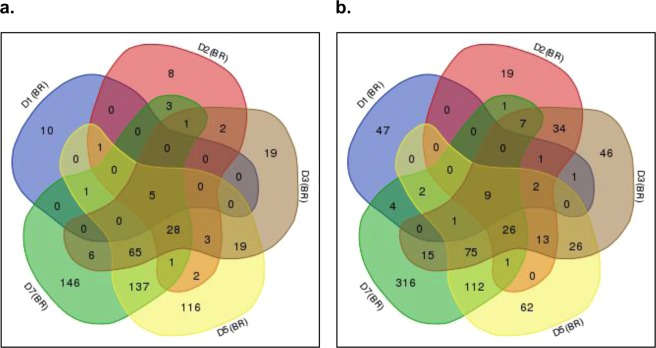
Number of specific and common DEGs in bacteria treated with roots (BR) at different timepoints. Venn diagrams were constructed using differentially expressed (**a**) up- and (**b**) down-regulated genes, to illustrate the number of genes expressed per time point and the genes common to more than one timepoint. Labels in the Venn diagrams D1(BR), D2(BR), D3(BR), D5(BR) and D7(BR) indicate the number of co-cultivation days or days after inoculation of the bacteria treated with roots. Venn diagrams were constructed with the genes associated with a *q*-*value* < *0*.*05* for at least one-time point as previously illustrated in Supplementary Fig. S3. Only one up-regulated (OV14_RS18105) and one down-regulated gene (OV14_RS29425) was shared between timepoints D0-D5 and D0-D7 respectively, data not shown in the diagrams. The full list of genes, accession numbers, location and function associated to these Venn diagrams are available in Supplementary Table S2.

Visualizing how the DEGs from Fig. 2 were either independently expressed at specific timepoints or present across several timepoints, a total of 10, 8, 19, 116 and 146 up-regulated genes and 47, 19, 46, 62 and 316 down-regulated genes (located on the edges of the diagram) were found to be expressed at specific timepoints D1, D2, D3, D5 and D7 respectively (Fig. 4). A core set of five up-regulated and nine down-regulated genes were common to all timepoints with eight out of 14 of potential relevance in virulence and host defence evasion (Table 1). Day 0 (D0) is not shown in the Venn diagrams; only one out of six up-regulated genes (OV14_RS18105) was shared between D0 and D5 and no down-regulated genes were shared between D0 and any other time points. The full list of genes, accession numbers, location and function associated to the Venn diagrams are available in Supplementary Table S2.

**Table 1:** Functional detail of five (up-regulated) and nine (down-regulated) genes differentially expressed from D1 to D7 located in the centre of the Venn diagrams from Fig. 3.

Gene ID	Location	Gene name	Bacterial function	Reference	BLAST% identity
**Up-regulated**					
*OV14_RS24990*	Chromosome 2	Tetratricopeptide repeat protein (TPR)	Role in virulence mechanisms Translocation of virulence factors into host cells and adhesion to host cells.	Cerveny *et al*.^42^	74% *S*. *meliloti* 66% *R*. *leguminosarum* 67% *A*. *tumefaciens* complex
*OV14_RS24995*	Chromosome 2	Hypothetical protein	ND	—	73% *S*. *meliloti* No hit - *A*. *tumefaciens* complex
*OV14_RS16135*	Chromosome 1	Nucleoside-diphosphate sugar epimerase	Potentially in the evasion of the bacteria from the host immune system.	Sousa *et al*.^44^ Stiens *et al*.^45^	72% *S*. *meliloti* 54% *A*. *tumefaciens* complex
*OV14_RS30440*	pOV14b	Adenylate/guanylate cyclase domain-containing protein	Involved in the cellulose production in *Agrobacterium*, facilitates colonization of plant surfaces.	Ausmees *et al*. 2001	71% *S*. *meliloti* 60% *A*. *tumefaciens* complex
*OV14_RS21660*	Chromosome 2	Dioxygenase	Wide function: From energetic adenosine triphosphate (ATP) generation to xenobiotic degradation	Suenaga *et al*. 2009	89% *S*. *meliloti* 78% *A*. *tumefaciens* complex
**Down-regulated**					
*OV14_RS23300*	Chromosome 2	Rhizopine-binding protein	Involved in symbiosis. Rhizopines are compounds produced by bacterioids from symbiotic bacteria	Gordon *et al*. 1996 Spaink *et al*. 1998	93% *S*. *meliloti* 78% *A*. *tumefaciens* complex
*OV14_RS05820*	Chromosome 1	gfo/Idh/MocA family oxidoreductase	Potentially involved in rhizopine metabolism	Dilworth *et al*. 2008	88% *S*. *meliloti* 58% *A*. *tumefaciens* complex
*OV14_RS18300*	Chromosome 1	Inositol 2-dehydrogenase	Inositol catabolism is involved in rhizopine utilization in *S*. *meliloti* Associated in the formation of healthy nodules in *S*. *fredii* from soybean.	Galbraith *et al*.^48^ Jiang *et al*.^49^	91% *S*. *meliloti* 72% *A*. *tumefaciens* complex
*OV14_RS05810*	Chromosome 1	5-dehydro-2-deoxygluconokinase	Inositol catabolism	Anderson *et al*. 1971	92% *S*. *meliloti* 69% *A*. *tumefaciens* complex
*OV14_RS23305*	Chromosome 2	Sugar ABC transporter ATP-binding protein	ABC transporters potentially involved in virulence	Davidson *et al*. 2008	93% *S*. *meliloti* 86% *A*. *tumefaciens* complex
*OV14_RS14055*	Chromosome 1	Phytanoyl-CoA dioxygenase	Catalyzes the α-hydroxylation of phytanoyl-CoA	You *et al*. 2006	86% *S*. *meliloti* 78% *A*. *tumefaciens* complex
*OV14_RS23310*	Chromosome 2	ABC transporter permease	ABC transporters potentially involved in virulence	Davidson *et al*. 2008	85% *A*. *tumefaciens* complex 70% *S*. *meliloti*
*OV14_RS00280*	Chromosome 1	Phage major capsid protein	ND Transferred by horizontal gene transfer??	BLAST result	71% *S*. *meliloti* 46% *A*. *tumefaciens* complex
*OV14_RS00295*	Chromosome 1	Terminase large subunit	Plays a role in DNA translocation and packaging termination in lambda bacteriophage	Duffy *et al*. 2002	30% *S*. *meliloti* 29% *A*. *tumefaciens* complex

Some of these functions suggest that these genes might be related to the transformation process.

ND: Not determined.

### Gene ontology (GO) enrichment analysis of DEGs

**Figure 5:**
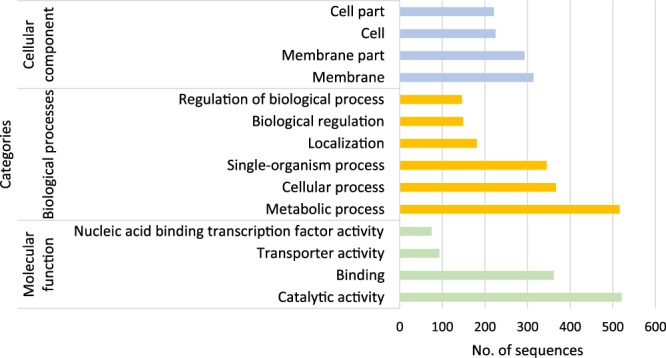
Distribution of the gene ontology (GO) categories assigned to the EOV14_5105 transcriptome using Blast2GO. 1345 complete CDS were used for the Gene Ontology (GO) analysis with 435, 699 and 752 sequences grouped in three main categories of cellular components (GO:0005575), biological processes (GO:0008150) and molecular functions (GO:0003674) using Blast2GO.

Out of the 2333 DEGs initially identified, 1345 with complete CDS were used for the Gene Ontology (GO) analysis with 435, 699 and 752 sequences grouped in three main categories of cellular components (GO:0005575), biological processes (GO:0008150) and molecular functions (GO:0003674) using Blast2GO. In the cellular component, 72% and 67.3% of the sequences were associated with membrane components (GO:0016020 and GO:0044425), whereas 51.7% and 50.8% were associated with cell/cytoplasm components (GO:0005623 and GO:0044464). In the biological processes, the sub-categories of metabolic processes (GO:0008152), cellular processes (GO:0009987), single organism processes (GO:0044699) and localization (GO:0051179) were enriched 73.82%, 52.5%, 49.3% and 25.8% respectively. Finally, for metabolic function the enriched sub-categories were catalytic activity (GO:0003824), binding (GO:0005488), transporter activity (GO:0005215) and transcription factor activity (GO:0001071) with 69.2%, 48.1%, 12.3% and 9.9% respectively (Fig. 5). Further analysis using BlastKoala and KEGG indicated that out of the 1345 protein sequences uploaded, 1211 (>90%) showed evident orthology with sequences belonging to the α-proteobacteria class and 44.6% were fully annotated in the KEGG database, with the overrepresented functional categories corresponding to Environmental Information Processing (EIP), Cellular Processes (CP) and Genetic Information Processing (GIP).

### Transcriptional activity of genes located on the pC5105 unitary plasmid

In the absence of *A*. *thaliana* roots but presence of 200 µM of acetosyringone, a higher level of transcription was recorded across the suite of *vir* genes on the pC5105 plasmid (Supplementary Table S3), relative to their expression following co-cultivation with roots (Supplementary Table S4). While 200 µM acetosyringone was present in both B and BR treatments, a general (q > 0.05) decrease in the FPKM values in the BR treatment compared to B was consistent across all the *vir* genes. Significant differences in expression (q < 0.05) when comparing B and BR within timepoints were only found for *virE1*(D3), *virE2*(D5), *virK*(D7), *virB6*(D7), *virD1*(D7).

**Figure 6:**
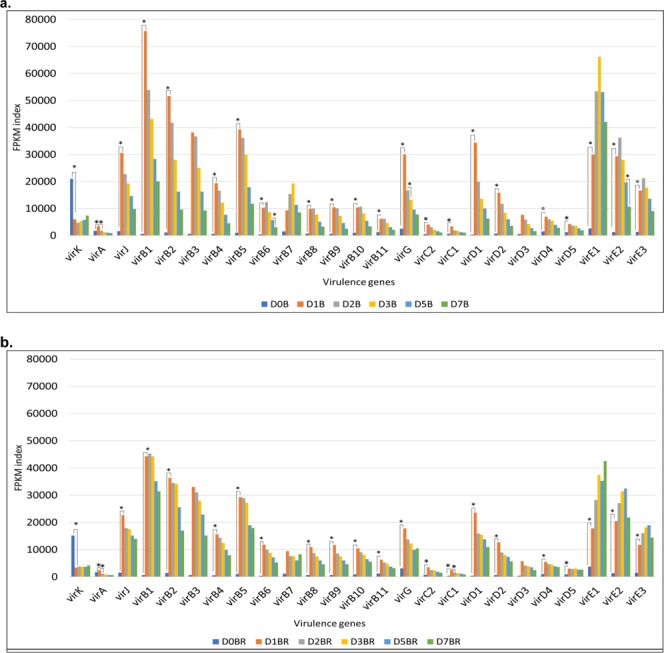
FPKM expression values of 25 *vir* genes from plasmid pCAMBIA5105. Gene expression of twenty-five *vir* genes measured across 6 timepoints in (**a**) EOV14_5105 only (Bacteria only, B). Time points: D0B, D1B, D2B, D3B, D5B and D7B indicate the days after co-cultivation in bacteria without roots: day1 (D1B), day2 (D2B), day3 (D3B), day5 (D5B) and day7 (D7B) and (**b**) EOV14_5105 co-cultivated with *A*. *thaliana* roots (Bacteria plus roots, BR). Time points: D0BR, D1BR, D2BR, D3BR, D5BR and D7BR indicate the days after co-cultivation in bacteria with *A*. *thaliana* roots: day1 (D1BR), day2 (D2BR), day3 (D3BR), day5 (D5BR) and day7 (D7BR). Asterisks indicate where significant changes in expression were observed across timepoints (indicated by connecting lines) for each respective *vir* gene.

Comparing expression values across timepoints independent of treatment we found that 21 out of 25 *vir* genes (*virA*, *virJ*, *virB1*, *virB2*, *virB4*, *virB5*, *virB7*, *virB8*, *virB9*, *virB1*0, *virB11*, *virG*, *virC1*, *virC2*, *virD1*, *virD2*, *virD4*, *virD5*, *virE1*, *virE2*, *virE3*) were significantly induced in the transition from D0 to D1 (Fig. 6), with minimum FPKM values ranging from 326.85 and 372.47 (in D0) to maximum values of 75693 and 45249 (in D1) for B and BR respectively. (Supplementary Tables S3 and S4). Additionally, significant differences in expression were observed for *virA* and *virG* (D1–D2, treatment B), *virB6* and *vir*E2 (D5–D7, treatment B) and *virA* and *virC1* (D1–D2, treatment BR). (Supplementary Tables S3 and S4).

Of the genes induced, those belonging to *virA*, *J*, *B, G*, *C* and *D* operons showed peak expression at D1 irrespective of treatment, whereas *virK* was significantly down-regulated at the same timepoint (Fig. 6). For *virB6* and *virB7* expression peaks were noted at D2 and D3 respectively in the absence of roots while in the presence of root tissue peak expression shifted to D1 in both cases. For genes within the *virE* operon, maximal FPKM values in *virE1*, *virE2 and virE3* occurred at D3 and D2 in B (Fig. 6a) and at D7, D5 and D5 respectively in BR (Fig. 6b). Interestingly, and contrary to what was commonly observed in most of the *vir* genes from this study, in *virE1* from BR (Fig. 6b), peak expression was noted at D7, compared to a peak of expression at D3 in the absence of roots (Fig. 6a).

### Validation of RNAseq data with relative gene expression analysis

**Figure 7:**
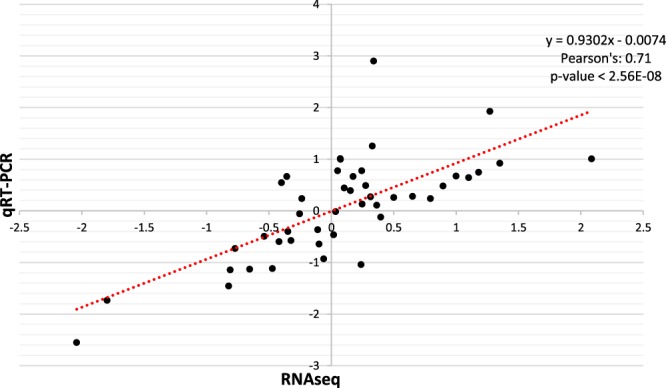
Validation of RNAseq data with corresponding quantitative real time qRT-PCR analyses. Correlation plot between RNAseq and qRT-PCR data for log_2_fold changes between BR and B conditions of nine genes in six consecutive time-points. Pearson’s correlation coefficient = 0.71, p-value < 2.56E-08.

Correlation analysis of the log_2_fold changes between B and BR treatments from generated RNAseq and qRT-PCR data of nine selected genes (*virB1*, *virK*, *OV14_RS30665*, *OV14_RS18490*, *OV14_RS225*05, *OV14_RS31975*, *OV14_RS30910*, *OV14_RS16760* and *trbD*) returned a positive significant relationship: r = 0.71, p-value < 2.56E-08 (Fig. 7 and Supplementary Fig. S1). The genes used for validation were selected because of their potential relevance in the process of transformation (plant-bacteria interaction), and their differential patterns of expression, which are suitable for comparing both techniques (RNAseq-vs-qPCR). Relative expression analysis (Supplementary Fig. S7) indicated strong up-regulation of *virB1* from D0 to D1 and then consistent down-regulation up to D7 for both the B and BR treated samples (Supplementary Fig. S7a). In contrast, *virK* was down-regulated from D0 to D1 and then upregulated from D3 through to D7 (Supplementary Fig. S7b). Genes *OV14_RS30665*-FMN-binding protein (Supplementary Fig. S7c) and *OV14_*RS18490-DUF2325 (Supplementary Fig. S7d) showed a significant decrease in expression from D0 to D1, remaining relatively constant at a lower relative expression levels up to D7. Gene *OV14_RS22505* which encodes an exopolysaccharide production repressor-type protein (*exoX*) increased its expression from D1 to D7 (Supplementary Fig. S7e). The membrane-related protein gene *OV14_RS31975* located in pOV14b exhibited a constant expression throughout the time course (Supplementary Fig. S7f). The expression of *OV14-RS30910* (cell-envelope protein) located on pOV14b was up-regulated from D0 to D1 (Supplementary Fig. S7g) as was *OV14_RS16760* (*chvD*) which encodes a chromosomal virulence D ortholog in EOV14_5105 (Supplementary Fig. S7h). Separately, the *trbD* (conjugal-transfer protein) gene with 97% identity with *A*. *tumefaciens trbD* was steadily up-regulated from D0 to D7 (Supplementary Fig. S7i).

### Genetic pathways in *E*. *adhaerens* OV14 responsive to the presence of plant tissue

#### ABC-transporters and bacterial secretion systems

**Figure 8:**
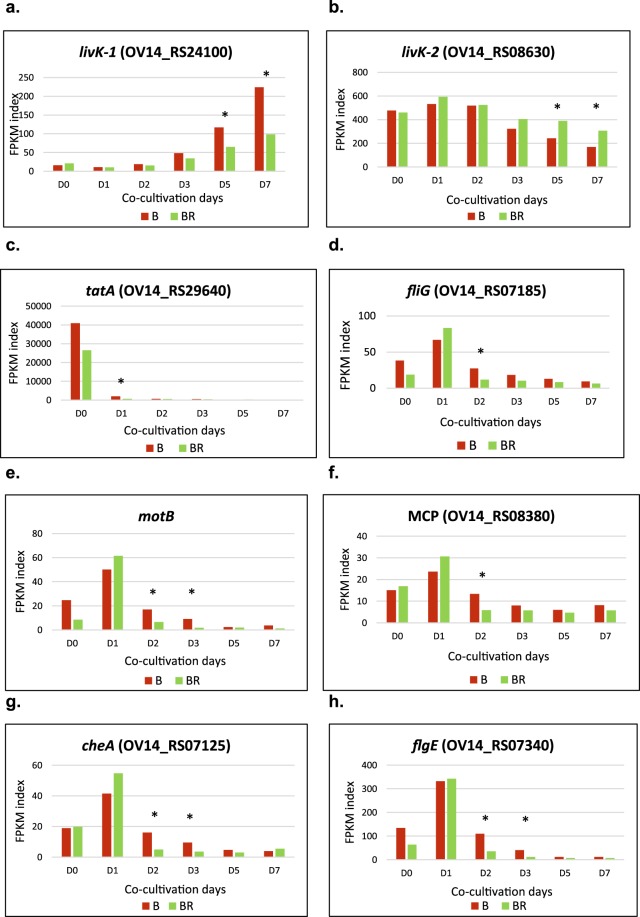
Temporal FPKM expression values for genes belonging to the categories environmental information processing, cellular processes and processing of genetic information as determined using the KEGG database. D0 to D7 indicates abbreviations for co-cultivation days as described in materials and methods. Asterisks indicate evidence of significant differential expression using Tophat2 v2.1.1 (p < 0.05) between EOV14_5105 un-treated (B) and treated with roots (BR) for three biological replicates. Red and green bars indicate the gene expression level for EOV14_5105 un-treated (B) and treated bacteria (BR) respectively in regards to (**a**) *livK (OV14_RS24100);* quorum sensing, (**b**) *livK (OV14_RS08630);* quorum sensing, (**c**) *tatA*; cell motility and bacterial chemotaxis (**d**) *fliG*, (**e**) *motB*, (**f**) *MCP*, (**g**) *cheA* and flagellar assembly (**h**) *flgE*.

A total of 1345 protein accessions were uploaded in BlastKOALA of which 44.9% had an annotated function in the KEGG database. The KEGG database provided us with a KEGG identifier linked to each protein ID uploaded, with three main categories found: (i) genetic information processing (ii) cellular processes and (iii) processing of genetic information (Supplementary Table S5). Under the environmental information processing category the sub-category membrane transporters is composed of two sub-groups: ABC-transporters and Bacterial Secretion System. Within the ABC-transporters group there were 54 KEGG Orthology (KO) identifiers, associated with a total of 72 protein ID sequences. This represented the highest number of identifiers found in a single sub-group. Most of the genes from this group were related to oxidative phosphorylation; however, four KO identifiers including eight protein ID sequences were related to quorum sensing (Supplementary Table S5). The genes (*livK*, *livH*, *livM* and *livF*) identified that relate to quorum sensing act together in the same biosynthetic pathway with two accessions of *livK-1* and *livK-2* (OV14_RS24100 and OV14_RS08630) reporting contrasting expression patterns. In general terms, *livK-1* was induced and *livK-2* was repressed across timepoints (Fig. 8a,b). *LivF*, *livH* and *livM* were found to be down-regulated in respect to D0 under both B and BR conditions (Supplementary Fig. S8). Examining the effect of root tissue, significant (P < 0.05) changes occurred towards the end of the time course (D5 and D7) for *livK-1* and *livK-2* (*OV14_RS24100*, *OV14_RS08630*), *livH* (*OV14_RS18800*) and *livM* (*OV14_RS18795*) (Fig. 8a,b and Supplementary Fig. S8). Only *livK-2* (OV14_RS08630) was up-regulated in BR respect to B, with the other genes from this group showing a reduced expression in the presence of roots.

For the bacterial secretion system sub-category, a single identifier with a single representative gene (*tatA*) was identified. Highly expressed at D0, *tatA* activity abruptly decreased by D1. This continued through to D7 irrespective of the treatment (B, BR). *tatA* was the only gene associated with a bacterial secretion system that was found to be differentially expressed in the presence of root tissues (Fig. 8c).

#### Bacterial chemotaxis and flagellar assembly

The genes *fliG*, *motB*, *mcp*, *cheA*, *fliM*, *fliNY/fliN*, and *cheY* associated with bacterial chemotaxis were found to be differentially expressed under the BR treatment. All these genes exhibited a significant induction up to D1, after which genes were strongly repressed. Specifically for D1, the abundance of transcripts was higher in BR compared to B (Fig. 8d–g [*fliG - cheA*] and Supplementary Fig. S9 [*fliM - cheY*]). In the presence of roots the genes *fliG*, *fliM* and *mcp* exhibited significant down regulation at D2, *motB*, *cheA* and *fliNY* at D2 and D3 and *cheY* at D3 and D7 (Fig. 8d–g and Supplementary Fig. S9). Additionally, 14 DEGs involved in the structural formation of flagella were also identified (Supplementary Table S5). The genes *flgE*, *flgC*, *flgF*, *flgG*, *flgK*, *flgL*, *fliC*, *fliF*, *fliL*. *flgC*, *flgI*, *fliF*, *fliG* and *fliM* similarly to those previously mentioned for bacterial chemotaxis, recorded highly induced FPKM values at D1, which were down-regulated in the subsequent co-cultivation days in bacteria treated and untreated with roots (Fig. 8h [*FlgE*] and Supplementary Fig. S10 [*FlgC – fliM*]). Groups of genes showed significant down-regulation in bacteria treated with roots (BR) at different timepoints during co-cultivation, the genes *flgC*, *flgF*, *fliC*, *fliF* showed significant down-regulation at D2, *fliL* at D3, *flgE*, *flgG*, *flgL* at D2 and D3 and finally *flgK* at D2, D3 and D7. The expression profiles of the genes *flgC*, *flgF*, *flgG*, *flgK*, *flgL*, *fliC*, *fliF*, *flil*. and KEGG diagram of the genes involved in flagellar assembly can be found in Supplementary Fig. S10. Genes involved in intertwined processes such as bacterial chemotaxis and flagellar assembly showed overall a similar expression pattern in the presence and absence of plant tissue throughout the co-cultivation period.

Genes considered (by sequence homology) in^12^ to be potentially involved in the transformation process include *chvA*, *chvB*, *exoC*, *gabD*, *attL*, *blcB*, *blcC*, *celA*, *celB*, *celC*, *celE*, *celG*, *pcs*, *pmtA*, *choX choV*, *choW*, *kdgF ligE*, *chvD*, *chvE*, *gguA*, *gguB*, *gguC*, *chvH*, *serB*, *miaA*, *acvB katA*, *ParA/VirC1*, *trbB*, *trbC*, *trbD*, *trbE*, *trbJ*, *trbK*, *trbL*, *trbF*, *trbG*, *trbH*, *trbI*, *aopB*, *vbp2-3*, *vbp1*. Out of the previously mentioned genes, *aopB* and *trbD* exhibited significant down-regulation in presence of plant tissue (BR) in later stages of transformation with EOV14_5105 (Supplementary Table S6). *aopB* in *A*. *tumefaciens* is regulated by the *chvD/chvI* two component system and is indirectly involved in virulence, whereas *trbD* is related to plasmid mobilization between bacterial cells.

#### Homologous symbiosis-related genes showing differential expression in E. adhaerens OV14

Based on the *in silico* comparison between *S*. *meliloti* and EOV14_5105 symbiosis-related protein sequences from previous reports^33,34^, we found genes with comparable expression trends after exposure to plant tissues (Table 2). In 2009 Gurich *et al*. observed that suppression of flagellum production and thus down-regulation of flagella-related genes in *S*. *meliloti* plays an important role for the effective nodule formation of *M*. *truncatula* roots. Here, the flagella-related genes *OV14_RS07290*, *OV14_RS07305* and *OV14_RS07300* found in EOV14_5105 (showing >45% identity with *S. meliloti*) were heavily repressed in the BR treatment (Table 2). In fact, our previously depicted results in Fig. 8d–h and Supplementary Figs S9 and S10, show how genes related to assembly and functionality of flagella were heavily repressed in the BR treatment.

**Table 2:** Symbiosis-related genes induced/repressed in *S*. *meliloti* and *E*. *adhaerens* in early stages of plant tissue colonization.

Protein ID *S*. *meliloti*^£^	Protein ID *E*. *adhaerens*	Gene ID *E*. *adhaerens*	Chr. location in *E*. *adhaerens*	BLAST% identity	Response in *S*. *meliloti*	Response at early stage^¥^ in *E*. *adhaerens*	Function	Reference
SMc00371	WP_025430719.1	OV14_RS32210	pb	75%	Down-regulated^¥^	Down-regulated* (D3-D7)	ferritin-like domain-containing protein	Capela *et al*.^33^
SMc00371	WP_025430830.1	OV14_RS32800	pb	61%	Down-regulated^¥^	Down-regulated* (D3-D7)	ferritin-like domain-containing protein	Capela *et al*.^33^
SMc00371	WP_025428892.1	OV14_RS22675	2	50%	Down-regulated^¥^	Down-regulated* (D3-D5)	ferritin-like domain-containing protein	Capela *et al*.^33^
SMc00796	WP_025426013.1	OV14_RS36805	1	91%	Down-regulated^¥^	Down-regulated* (D2-D7)	DUF1328 domain-containing protein	Capela *et al*.^33^
SMc00796	WP_025427496.1	OV14_RS36825	1	55%	Down-regulated^¥^	Down-regulated* (D2-D7)	DUF1328 domain-containing protein	Capela *et al*.^33^
SMc00800	WP_025426018.1	OV14_RS07740	1	73%	Down-regulated^¥^	Down-regulated* (D3-D7)	hypothetical protein	Capela *et al*.^33^
SMc00885	WP_025426083.1	OV14_RS08080	1	59%	Down-regulated^¥^	Down-regulated* (D3-D7)	photosystem reaction center subunit H	Capela *et al*.^33^
SMc01467	WP_025426897.1	OV14_RS12335	1	43%	Down-regulated^¥^	Down-regulated* (D3-D7)	hypothetical protein	Capela *et al*.^33^
SMc01580	WP_025427900.1	OV14_RS17535	1	80%	Down-regulated^¥^	Up-regulated* (D5-D7)	marine proteobacterial sortase target protein	Capela *et al*.^33^
SMb20661	WP_025429803.1	OV14_RS27445	2	68%	Down-regulated^¥^	Up-regulated* (D5-D7)	sn-glycerol-3-phosphate ABC transporter ATP-binding protein UgpC	Capela *et al*.^33^
SMc00280	WP_025430740.1	OV14_RS32325	pb	30%	Down-regulated^¥^	Up-regulated* (D3-D7)	hypothetical protein	Capela *et al*.^33^
SMc01718	WP_025425752.1	OV14_RS06360	1	62%	Down-regulated^¥^	Up-regulated (D3)	TVP38/TMEM64 family protein	Capela *et al*.^33^
ccmA (WP_025425187)	WP_018097396.1	OV14_RS03440	1	80%	Down-regulated^¥^	Up-regulated (D2-D7)	Maturation of cytochrome *c*	Capela *et al*.^33^
SMb21456.1	WP_081788833.1	OV14_RS05910	1	36%	Down-regulated^¥^	Up-regulated (D2-D7)	chromosomal replication initiator protein DnaA	Capela *et al*.^33^
SMa0763	WP_025429268.1	OV14_RS24650	pb	48%	Down-regulated^¥^	Up-regulated (D5-D7)	pyridoxamine 5′-phosphate oxidase family protein	Capela *et al*.^33^
flaA	WP_025425931.1	OV14_RS07290	1	71%	Down-regulated^ζ^	Down-regulated* (D3)	Flagellin	Gurich *et al*.^34^
flaA	WP_025425932.1	OV14_RS07305	1	49%	Down-regulated^ζ^	Down-regulated* (D3)	Flagellin	Gurich *et al*.^34^
flaA	WP_081788891.1	OV14_RS07300	1	58%	Down-regulated^ζ^	Down-regulated* (D2-D3)	Flagellin	Gurich *et al*.^34^

^¥^Early stage: 5 days post inoculation.

^ζ^Evaluation 4-weeks post inoculation.

Induction/repression of expression in EOV14_5105 in contact with roots (BR) respect to bacteria un-treated with roots (B).

## Discussion

The genome of *E*. *adhaerens* OV14 has been previously characterized and compared with that of the alternative rhizobia *A*. *tumefaciens* C58 and *S*. *meliloti* 1021^12^. Yet, prior to this study the relative activity of the *E*. *adhaerens* OV14 genome in the presence and absence of plant tissues was undetermined. Indeed, the results presented here are novel not only in their capacity to provide insight into the genetic processes underway in a non-*Agrobacterium* spp but they are also the first survey of whole genome transcription in a plant engineering bacterium following co-cultivation with plant tissues.

Plant tissue conditions used were as per detailed in the *Arabidopsis* root assay^35^, which was a modified version of the original *A*. *tumefaciens*-based assay^36^. This provided a rapid and simple method of co-cultivating EOV14_5105 with root tissues and critically allowed for the rapid isolation of EOV14_5105 cells at each timepoint. Based on this assay, an optimum rate of transient transformation with *A*. *thaliana* roots was achieved after 5 days of co-cultivation. This contrasts with *A*. *tumefaciens*, where the optimal co-cultivation period for most plant species is typically 2–3 days^18^. The extended co-cultivation period is a requirement of EMT that has been used in the transformation of the recalcitrant rice variety IR64^5^ and in several different plant species (e.g. potato, barley, wheat and oilseed rape) transformed using EOV14_5105 in our laboratory.

Here, the number of DEGs increased across timepoints in the presence of roots. The highest fold-change in the number of DEGs occurred between D0 and D1 co-cultivation. It is reasonable to think that a large proportion of genes potentially related to the plant-bacteria interaction and specifically to plant transformation are triggered at the early stages of the plant-prokaryote interaction. In *A*. *tumefaciens*, virulence genes directly related to transformation are triggered in the presence of plant tissue or phenolic compounds within 4 to 12 hours after inoculation^24–26^. Also, chromosomal genes such as *chvE/I*, *aopB*, *rbsC*, *flaA*, *flaB*, *mcpA*, *cheY2* among others, have been found to be differentially expressed under acidic conditions suitable for transformation within 7 hours^37^. While, in *S*. *meliloti* nodulation, conjugal transfer and invasion related genes can be induced in bacteria under symbiotic conditions as early as 18 hours post inoculation^38–40^. These findings reinforce our assumption that many of the DEGs triggered here within the first 24 hours could be related to an early plant-bacteria sensing/attachment interaction and/or might be precursor genes that act as initiation factors involved in transformation-related pathways downstream in the process.

In broad terms the DEGs found in the BR treatment were represented across all four replicons from EOV14_5105. This supports the conclusion of Rudder^12^ who proposed the presence of chromosomal homolog genes from *A*. *tumefaciens* within EOV14_5105. However, when dissecting the process within the different co-cultivation days, an overrepresentation of DEGs derived from pOV14b was observed at day 1 (D1), which concurs with *A*. *tumefaciens* and *S*. *meliloti*, where most of the genes related to virulence and symbiosis respectively are also located on plasmid structures^18,41^.

In this study we found a core set of 14 DEGs consistently activated from D1 through to D7. Eight are related to virulence and/or symbiosis. The up-regulated gene *OV14_RS24990* with BLAST identity of 74% and 67% with *S*. *meliloti* and *A*. *tumefaciens* identified as Tetratricopeptide repeat protein (TPR) present in prokaryotic and eukaryotic organisms, mediates protein–protein interactions and is related to the virulence potential of pathogenic bacteria such as *Y*. *pestis* and *P*. *aeruginosa*^42^. The gene *OV14_RS30440* (71% identity with *S*. *meliloti*) contains an adenylate/guanylate cyclase domain. In *Agrobacterium* and *Rhizobium* this domain is associated with cellulose production which helps in cell aggregation and anchoring to plant surfaces thereby facilitating colonization^43^. Finally, *OV14_RS16135* (72% identity with *S*. *meliloti*) is a nucleoside-diphosphate sugar epimerase. Sugar nucleotides are relevant for the biosynthesis of sugar-containing bacterial cell structures. In pathogenic bacteria, this structure can lead to the evasion of the bacteria from the host immune system^44^. In *S*. *meliloti*, nucleoside-diphosphate sugar epimerase can be involved in cell envelope biogenesis, carbohydrate transport and metabolism^45^. Two steadily down-regulated genes (*OV14_RS23300* and *OV14_RS05820*) are related to rhizopine metabolism. Rhizopines are secreted plant compounds, which are synthesized by bacteroids inside the plant-nodule in *Rhizobium*-legume interactions^46^. The production of certain rhizopines can confer selective advantage to free-living cells located in the vicinity of the rhizosphere. In 2014 Krysciak^47^ and colleagues discovered that genes responsible for rhizopine catabolism in *S*. *fredii* NGR234 were repressed in the presence of N-acyl-homoserine-lactones. They hypothesized that the downregulation is due to a link between rhizopine metabolism and quorum sensing signals mediated by the activation of the conjugative gene *traR*^47^. In EOV14_5105, *traR- OV14_RS28560* (82% identity with *S*. *meliloti*) (located in pOV14c) was up-regulated in the presence of roots at D1 (p = 0.0061; q = 0.234). The possibility of a link between the down-regulation of the rhizopine-related genes by *traR* gene and its involvement in transformation in EOV14_5105 requires further clarification. The genes *OV14_RS18300* (91% identity with *S*. *meliloti*) coding for inositol 2-dehydrogenase and *OV14_RS05810* (92% identity with *S*. *meliloti*) for 5-dehydro-2-deoxygluconokinase are involved in inositol catabolism. Catabolism of inositol is essential for the utilization of rhizopines as a carbon source in *S*. *meliloti* L5–30^48^ and inositol 2-dehydrogenase is also involved in the formation of healthy nodules in soybean colonized by *S*. *fredii*^49^. Lastly, the gene *OV14_RS05820* a sugar ABC sugar transporter (93% identity with *S*. *meliloti*) containing ribose (RbsA) and galactose (MglA) import ATP-binding motifs was identified. Sugars available extracellularly in bacteria can be transported into the cytoplasm by ABC transporters^50^. In *A*. *tumefaciens* the ABC transporter chvE-MmsAB is crucial for sugar utilization and virulence^51^. Bacteria can optimize energy usage by taking in certain available sugars; for this, repression of specific sugar uptake pathways is required^52^. The mechanism by which the *OV14_RS05820-*sugar transporter is downregulated throughout all timepoints in the presence of roots and its involvement in transformation remains to be elucidated.

In *A*. *tumefaciens* the *vir* genes are tasked with the transfer of T-DNA complex from bacteria to host cells^18,19,53,54^. In this study, *E*. *adhaerens* OV14 was equipped with an *A*. *tumefaciens* derived suite of *vir* genes on the pC5105 plasmid. The induction effect of acetosyringone on the activation of the virulence genes on the Ti plasmid from *A*. *tumefaciens* has been long known^25^ and this phenomenon has recently been described in *R*. *etli*^11^ and *O*. *haywardense* (patent WO2017040343A1)^55^. With the current EOV14_5105 genotype, the supplementation of co-cultivation media with acetosyringone is an important step for improving infection efficiency^8^. Here, all virulence genes (except *virK*) exhibited low levels of basal expression at D0, with a subsequent induction at D1, which was significant and in agreement with reports from^21,22,56,57^. In line with data reported by^24,25,58^, *virA* showed modest levels of induction in the presence of acetosyringone. This differs with the results obtained by^17^, where *virA* was expressed at basal constitutive levels and it was not found to be induced by acetosyringone. This difference can be attributed to the plasmid background present in our bacteria. In octopine (pTiA6) and nopaline (pTiC58)-type plasmids, expression of *virA* is constitutive^17^ and weakly inducible^58–60^ respectively. In our case, pCAMBIA5105 is derived from the ‘supervirulent’ succinaminopine-type plasmid pTiBo542, which is acetosyringone dependent^61^. Overall, these results highlight the effect of acetosyringone on EOV14_5105 gene expression relative to the limited number of *vir* genes from *A*. *tumefaciens* and *R*. *etli* evaluated to date in response to this compound^11,62–64^.

While both B and BR treatments included acetosyringone, when co-cultivated with roots a decrease (non-significant) in *vir* gene transcription within EOV14_5105 was recorded in BR. Bolton and collaborators originally reported this phenomenon with tobacco protoplasts^24^. The underlying reason for this downregulation in EOV14_5105 is most likely due to a feedback system of repression arising from a MAMP triggered elicitation of the plants immune system^65,66^. For example, it is known that salicylic acid^67,68^ and ethylene-associated^69^ defense responses in plants can shut down the expression of the *vir* regulon suppressing *Agrobacterium*-mediated transformation. EOV14_5105 possesses the salicylate-hydroxylase gene (*OV14_RS11845*) which could potentially degrade plant derived salicylic acid. However, *OV14_RS11845* was not differentially expressed in bacteria treated with roots, suggesting that additional genes might be involved in the general down-regulation of the *vir* operon in the presence of roots. In considering known *Agrobacterium* MAMPs such as peptidoglycan^70^ and the EF-Tu elongation factor^71^, EOV14_5105 does indeed possess homologs of several peptidoglycan-binding type proteins and EF-Tu, but neither registered significant levels of expression in the BR treatment under the conditions of this study. Meanwhile the absence of a functional Type III secretion system in EOV14_5105 would suggest the absence of a delivery system for the secretion of specialized effector proteins directly into plant cells, which is a recognized mechanism for stimulating a plant’s defense response^72^. Of course, the stress induced by the physical cutting of the roots would have led to the release of defense compounds such as reactive oxygen species (ROS) among others that can decrease the activity of several metabolic pathways in bacteria^73,74^, which may have led to a general decrease in *vir* gene expression. Supporting this theory is the observation that the gene *OV14_RS09075*, a *superoxide dismutase-sodA* associated with the detoxification/elimination of ROS^75,76^ showed a significant induction in EOV14_5105 exposed to roots at D3, D5 and D7.

In *A*. *tumefaciens*, the VirB1 protein has been found to be extracellularly secreted, associated with the T-complex and linked to local lysis of the peptidoglycan wall^77^. This protein exhibited high levels of induction under our experimental conditions. The lipoprotein VirB7 stabilizes other VirB proteins and is associated with VirB2 in the T-pilus^78,79^. It is reasonable to assume therefore that it’s expression once initiated would be relatively constant during periods of bacterium-plant co-cultivation. In our results, whereas the expression of *virB7* significantly decreased in untreated bacteria, it remained stable in bacteria treated with roots. virE1 acts as a chaperone for virB2 facilitating its stabilization and solubilization^80,81^ while virE2 and virE3 are imported into the host cell together with virE2 also assisting in T-complex stability^82–84^. In light of the critical roles played by virE2 and virE3^64,82,85^ the delayed peak-expression of these genes, could explain the additional two days co-cultivation typically required in transformation experiments with EOV14_5105 compared to *A*. *tumefaciens* to obtain comparable infection efficiencies. Separately, a genetic complementation study has revealed that *virB* operon genes from the plasmid pTiA6 can mutually co-stabilize each other and that *virB1/virB2/virB3/virB4* and *virB7/virB8/virB9*/*virB10*, are probably translationally coupled^86^. Interestingly, our expression results showed a clear differentiation in the expression pattern of both group of genes after exposure to acetosyringone, suggesting that there might also be a feedback system acting between these group of genes within pCAMBIA5105.

*Vir* genes aside, the whole genome response triggered in EOV14_5105 following contact with roots provides an opportunity to survey complementary genes that support T-DNA transfer. For example, the majority of genes from the cellular component category were membrane-associated and the KEGG database analysis retrieved 55 genes related to metabolic pathways that could be relevant to the transformation process. Genes from the *liv* operon are involved in the transport of branched-chain amino acids in *E*. *coli*^87^ and *R*. *leguminusarium*^88^. Branched-chain amino acids are essential for symbiosis in bacterioid structures in *R*. *leguminusarium*^88^. LivH/LivM and LivF/LivG are hydrophobic and hydrophilic components of the membrane structure respectively. LivK is a substrate binding protein, LivF is an ATP binding protein and LivH is part of the inner membrane complex encoding for a large hydrophobic protein. According to^12^ key genes involved in nodulation and nitrogen fixation are absent in EOV14_5104. This leads to a series of questions: are *liv* genes fulfilling a function related to N_2_ fixation in EOV14_5105? Is the intake of branched-chain amino acids essential for transformation in EOV14_5105? Or is the differential expression of the *liv* genes related to plant transformation?

An intact TAT secretion system is vital for *A*. *tumefaciens* infection of plant cells^89^ with the *tat* operon involved in the transfer of folded proteins from the cytoplasm into the cell periplasm^90^, cell division, chemotaxis and flagellar biogenesis^89^. EOV14_5105 *tatA* was significantly down-regulated at D1 in the presence of roots and approximately 34-fold in response to acetosyringone through the time course. Interestingly, this gene reached FPKM values >40,000 which is equivalent to expression levels observed with the virulence genes. The significant decrease in *tatA* activity after exposure to roots and acetosyringone suggests that there is a repression of accessory protein export systems possibly to re-direct energy towards transformation-specific protein export machinery from the *vir* genes. Crosstalk between Tat and Sec secretions systems has been shown in *Streptomyces*^91^, also sequential regulation of secretion systems has been evidenced using Boolean modelling in *S*. *typhymorium* between elements of T3SS and T6SS^92^.

The processes of biofilm formation, flagellar assembly and quorum sensing are closely intertwined and related to virulence^93,94^. In *A*. *tumefaciens* biofilm formation deficiency is correlated with a reduced virulence phenotype^83^ and a decreased ability to colonize roots in *S*. *meliloti*^95^. In a heterologous system using *E*. *coli* to evaluate *A*. *tumefaciens* genes, a mutation in *crp* dramatically reduced the expression of the lacZ gene under the control of the promoter from the *virB* operon^96^. *Crp* (cyclic AMP receptor protein) controls biofilm production in pathogenic bacteria^97–101^ and not surprisingly in this study *crp* was significantly increased in the presence of root tissues from D2 through to D7, suggesting a possible contribution of biofilm formation in enhancing surface attachment of EOV14_5105 with host tissues. The presence of functional flagella on EOV14_5105 has been confirmed (Rathore *et al*. unpublished) and 14 DEGs involved in flagella structural assembly and motor responses were identified here. All these genes were initially induced by acetosyringone and subsequently repressed after 24 h in the presence and absence of roots. Reduced motility of bacteria in response to high concentrations of plant inducers helps to maintain a close contact between bacteria and plant tissue possibly contributing in the process of transformation^102^.

Chemotaxis is a crucial step for *A*. *tumefaciens’* colonization of the plant surface^93,102^. *A*. *tumefaciens* and *S*. *meliloti* chromosomal genes relevant for chemotaxis include *MCP* ((Methyl-accepting chemotaxis, which structurally resembles *virA*), *cheA*, *cheY* (resembles *virG*), *fliG*, *fliM*, *fliNY* and *motB* which act together in a pathway where the chemotactic stimuli are detected by the transmembrane MCP sensor protein^93,103–107^. Located on chromosome 1, all of the aforementioned genes had a strong response to acetosyringone. After D1 however, all these genes were heavily repressed, which coincides again with the observation that a high concentration of inducers (e.g. acetosyringone + plant-based inducers) can suppress chemotaxis and motility responses in *A*. *tumefaciens*^102^.

The *rseP* gene (Regulator of Sigma E, Protease) encodes a protease involved in the regulation of cell surface signaling systems (CSS) in *P*. *putida*^108^ and *P*. *aeruginosa*^109^. Little is known about the function of *rseP* in *A*. *tumefaciens*; however, it has been documented that similar proteins called Lon-proteases are required for normal growth, cellular morphology and full virulence^110^. Additionally, in *S*. *meliloti* Lon is involved in the regulation of exopolysaccharide synthesis and is required for effective nodulation in alfalfa^111^. In our results, *rseP* was triggered in the presence of roots, suggesting that these proteases are involved in the regulation of cell surface signaling systems in EOV14_5105. As an additional note on bacterial surface/secreted proteins, it has been found that exopolysaccharides (EPS) can suppress host defense responses towards *S*. *meliloti*^112^. Interestingly, in EOV14_5105 the gene OV14_RS22500 (exopolysaccharide biosynthesis protein-*ebp*) is significantly induced in the presence of roots at D5 and D7 (when T-DNA transfer is more pronounced). The suppression of host defense response by triggering the synthesis of exopolysaccharides in EOV14_5105 could therefore be enhancing host susceptibility by facilitating a closer interaction between plant and bacteria.

To summarize, the work detailed provides a novel insight into the genetic processes that underpin EMT across the examined timecourse. This study has characterized the temporal response of the EOV14_5105 genome to acetosyringone as well as to the presence of plant tissues. As the first published study describing the transcriptional response of a plant transforming bacterium in the presence/absence of plant tissues exposed to acetosyringone and with the recent identification of additional non-*Agrobacterium* species such as *R*. *etli* and *O*. *haywardense*, the presented datasets provide a benchmark for future studies focused on improving plant transformation processes in order to facilitate downstream applications.

## Materials and Methods

### *E*. *adhaerens* OV14_pC5105 growth conditions

Strain *E*. *adhaerens* OV14_pC5105 (henceforth EOV14_5105) was generated as described previously^8^ and cultivated overnight in 20 ml of YEP liquid media at 28 °C and 220 rpm. Thereafter, bacteria were harvested at O.D 1.0, centrifuged at 4000 rpm for 10 minutes and re-suspended in the same volume of filter sterilized Inoculation Media (IM; 4.4 g/l Murashige and Skoog salts plus Gamborg B5 vitamins, 200 mg/l hydrolysate casein, 10 g/l sucrose, 0.5 g/l MES, pH5.5). This bacterial suspension was incubated at 21 °C, 220 rpm for up to 60 minutes and used to inoculate *Arabidopsis* thaliana root tissues.

### Plant material preparation and co-cultivation with EOV14_5105

The preparation and growth of *A*. *thaliana* roots for co-cultivation with EOV14_5105 was completed *in-vitro* as previously described^35^. A root-based assay was selected because of its logistical advantages and also because *E*. *adhaerens* is a soil bacterium and under natural conditions has been shown to colonize root systems^8^. For co-cultivation, roots of approximately 100 mm in length from up to ten plants per treatment were aseptically cut into ~5 mm fragments using a small amount of water in a petri dish. Cut roots were grouped into bundles that were transferred to solid co-cultivation media (4.32 g/l of MS salts, 1X vitamin solution (0.5 mg/l nicotinic acid, 0.5 mg/l pyridoxine, 0.5 mg/l thiamine-HCl), 100 mg/l myo-inositol (stock 100X), 0.5 g/l MES, 10 g/l sucrose, 7.5 g/l Agar technical No. 3 (Oxoid LP0011), adjusted to pH 5.5 and supplemented with 200 µM of sterile-filtered acetosyringone post-autoclaving after media temperature reached 50 °C. Unless otherwise indicated media were autoclaved at 121 °C for 20 min and the supplier company for the reagents was Ducehfa Biochemie^®^. Excess of water was removed, and roots were immediately inoculated with 1 ml of EOV14_5105 resuspended in Inoculation Media. Roots were then manually spread to an approximate 2 cm radius to maximize contact with the media. Plates were co-cultivated at 21 °C in the dark for the designated period: day 0 (D0), day 1 (D1), day 2 (D2), day 3 (D3), day 5 (D5) and day 7 (D7) days post-inoculation. For day 0 (D0), bacteria were briefly pipetted onto roots, immediately removed and used for RNA extraction. After each co-cultivation time, collected suspensions were stabilized with 3 ml of RNAprotect Bacteria Reagent ®(Qiagen) for 5 min, vortexed for 5 min and the bacterial suspension collected using a micropipette. The suspension was then centrifuged at 12,000 rpm for 5 min after which the pellet was immediately placed in liquid nitrogen and transferred to −80 °C. In order to account for the potential variation in gene expression due to the time of co-cultivation, untreated bacteria (control) were handled as above for each timepoint but in the absence of *A*. *thaliana* roots. Three biological replicates (derived from three independent EOV14_5105 starter cultures) were processed for both treated and un-treated bacteria at six timepoints covering D0, D1, D2, D3, D5 and D7 (Supplementary Fig. S6). EOV14_5105 bacteria treated with *A*. *thaliana* roots was denominated as BR (bacteria + roots) and EOV14_5105 bacterium un-treated with roots was denominated as B (bacteria only). Supplementary Fig. S6.

Separately, a portion of treated roots from each respective timepoint was collected and histochemically stained to monitor EOV14_5105 infection efficiency, based on transient *GUS* gene expression^35^. Infection efficiency was recorded by counting the presence/absence of blue stained foci along each root length (T), but also semi-quantitative measurements were made by counting the number of roots with a single blue foci (class I), the number of roots with <50% surface area stained blue (class II) and finally the number of roots with >50% and <100% surface area stained blue (class III). Significant differences between staining levels were evaluated using ANOVA complemented with Tukey-Kramer post-hoc test (Microsoft Excel^®^). Supplementary Fig. S2.

### RNA isolation and quality verification

For RNA extraction, collected bacterial pellets were thawed on ice and then re-suspended in 300 µl of resuspension/lysis buffer (TE pH: 8.0, lysozyme 1 mg/ml and Proteinase K®, Qiagen). The following RNA extraction steps were performed in 1.5 ml vials using Trizol Reagent® as per manufacturer’s instructions. Pelleted RNA was washed with 70% ethanol, centrifuged, air dried (briefly, <1 min), re-suspended in ~30 µl of sterile Sigma® water and incubated at 65 °C for 10 min to evaporate residual ethanol. RNA was then precipitated and resulting pellets were once again washed with 70% ethanol, centrifuged, dried briefly, resuspended and incubated at 65 °C for 10 min. RNA quality was verified using a 2100 Bioanalyzer (Agilent Technologies; Santa Clara, CA, USA), with recorded RIN values for total RNA >7.4.

### rRNA depletion, cDNA library preparation and next-generation sequencing

rRNA depletion, cDNA library preparation and RNA sequencing was performed externally by the Beijing Genomics Institute (BGI, China). Briefly, rRNA depletion and fragmentation was carried out using Ribo-Zero Magnetic Kit (Bacteria, EPICENTRE). RNA was fragmented into 130–170 nt and purified with RNAClean XP Beads (AGENCOURT). cDNA synthesis was generated using First Strand Master Mix and Super Script II reverse transcription (Invitrogen). Adapters ligation was achieved by combining the Adenylate 3’Ends DNA, RNA Index Adapter, Ligation Mix and then purifying with DNA with Ampure XP Beads (AGENCOURT). Several rounds of PCR amplification with PCR Primer Cocktail and PCR Master Mix were performed to enrich the cDNA fragments. Resulting PCR products were purified using Ampure XP Beads (AGENCOURT), with the final library validated via an Agilent 2100 bioanalyzer instrument (Agilent DNA 1000 Reagents) and using real-time quantitative PCR (qRT-PCR) (TaqMan Probe). Qualified libraries were amplified on cBot to generate the cluster on the flowcell (TruSeq PE Cluster Kit V3–cBot–HS, Illumina). The amplified flowcell was sequenced under paired ends on a HiSeq. 4000 System (TruSeq SBS KIT-HS, Illumina), with an expected read length of 100 bp. Depth coverage was calculated at 10–12 million reads per sample, with 36 samples in total (6 timepoints × 2 treatments (B, BR) × 3 independent replications).

### Mapping and processing of sequencing data

Sequencing reads were trimmed using Trimmomatic v. 0.36 using paired end parameters. Mapping of reads to the *E*. *adhaerens* OV14 reference genome; acc. No. NZ_CP007236.1, NZ_CP007237.1, NZ_CP007238.1 and NZ_CP007239.1 and the plasmid pC5105 (EF042581) was carried out using Tophat2 v. 2.1.1 using parameters for paired end reads (tophat -p8 -G genes.gtf -o (name) indexgenome read_1.fq read_2.fq). Pairwise comparisons to identify DEGs were obtained using Cuffdiff v. 2.2.1 from the Cufflinks software package. Genes with a depicted [yes] in the excel Cuffdiff-output spreadsheet (False Discovery Rate-FDR or q_value < 0.05) were considered differentially expressed. Six pairwise comparisons were performed in Tophat2 in order to identify DEGs between both treatments (B, BR) at each time point (D0, D1, D2, D3, D5 and D7) (Supplementary Fig. S3). In addition, to visualize the induction dynamics exerted by acetosyringone across the timepoints, pairwise comparisons of pC5105 were carried out independently for both B and BR treatments across timepoints (Supplementary Fig. S4). As Tophat2 assigns the probability of observing each fragment in dataset uploaded, FPKM values found for the comparison between specific timepoints and across timepoints can vary because it corresponds to independent runs in the software^113^. After observing this phenomenon, we performed a Pearson correlation coefficient test using Microsoft Excel^®^ which delivered a value of r = 0.99, indicating that both datasets clearly provide the same information (Supplementary Fig. S5).

### Data mining and gene enrichment analysis

Exploration and visualization of the Cuffdiff output files was conducted in R studio using the package CummeRbund v. 0.1.3 and Microsoft Excel 2016. The DEGs depicted by [yes] in the excel Cuffdiff-output spreadsheet (q_value < 0.05) were retrieved for posterior analysis. Cuffdiff provides a log_2_fold change value in pairwise comparisons for each independent gene. Up- and down-regulated genes were retrieved based on a fold change >0 and <0 respectively. According to^114^ using the statistical correction of the q value using the FDR (False discovery rate: ≤0.05) can provide enough power to discriminate DEGs without the requirement of an extra cutoff value in the log_2_Fold change. To visualize which genes were exclusively expressed at certain time points and which ones were present in several time points, we listed the up- and down-regulated genes found separately from the pairwise comparisons for each independent time point (D0, D1, D2, D3, D5 and D7) and visualized the resulting groups of DEGs in two Venn diagrams using the online tool: http://bioinformatics.psb.ugent.be/webtools/Venn/ (Supplementary Fig. S3). Day 0 (D0) is not shown in the Venn diagrams; only one out of six up-regulated genes was shared between D0 and D5 and no down-regulated genes were shared between D0 and any other time points. The list of DEGs obtained from the Venn diagrams was associated with descriptive Gene Ontology (GO) terms using Blast2GO (v.4.1.5), via protein sequences and default parameters under the taxonomy filter for α-proteobacteria. At the same time, in order to retrieve maps of metabolic pathways potentially relevant for the transformation process, the resulting list of genes was analyzed with BlastKoala v. 2.1^115^ and KEGG^116–118^ (Kyoto Encyclopedia of Genes and Genomes) using default parameters within the taxonomy group prokaryotes-bacteria. BlastKOALA and KEGG allow for comparisons to be made for the functionality of individual genes or, in combination with larger number of genes, in complex metabolic pathways.

To investigate the potential commonality in the symbiotic relationship between *S*. *meliloti* and plant and that of *E*OV14_5105 and plant tissues a basic local alignment using BLAST (NCBI) of *S*. *meliloti* protein sequences with known function was carried out on the *E*. *adhaerens* OV14 genome. The protein ID and percentage of identity were recorded in order to evaluate the FPKM values corresponding to the retrieved-ortholologous protein in *E*. *adhaerens* OV14. This information was then used to evaluate if there was induction/repression of symbiosis-related genes in *E*. *adhaerens* OV14.

### Gene validation using qRT-PCR

To validate RNAseq datasets, the expression patterns of nine DEGs were independently analyzed using qRT-PCR. RNA samples of EOV14_5105 cultivated with root tissues were treated with RQ1 RNase-free DNase (M6101, Promega) as per manufacturer’s instructions, with the exception that the incubation time was increased to 60 min (at 37 °C). cDNA was synthesized using Random Hexamers™ and SuperScript™III Reverse Transcriptase system (LifeTechnologies). qRT-PCR reactions were prepared with in triplicate with three technical replications and samples were processed on a Lightcycler96® platform (Roche) using QuantiFast-Sybr®Green kit (Qiagen). Reaction setup was as follows: 2× QuantiFast SYBR GreenPCR Master Mix (1X), Primer forward (1 uM), Primer reverse (1 uM) and 100 ng of DNA per reaction, final volume of 25 µl was completed with RNase free water. Cycling parameters were as follows: PCR initial heat activation: 5 min at 95 °C; 2 step cycling (40 cycles): Denaturation for 10 sec at 95 °C and combined annealing/extension 30 secs at 60 °C, melting curve: 10 sec at 95 °C, 60 sec at 60 °C and 1 s at 95 °C. For the relative quantification of transcripts, the 2^−ΔCt^ method^119^ of was used, with target gene expression normalized against the *E*. *adhaerens* OV14 *secG* gene using the following equation:

$${2}^{-{\rm{\Delta }}\mathrm{Ct}}=\frac{{2}^{-{\rm{\Delta }}\mathrm{Ct}}({\rm{sample}})}{{2}^{-{\rm{\Delta }}\mathrm{Ct}}({\rm{reference}})}$$

The association of the RNAseq data v. qRT-PCR outputs was determined with Pearson’s correlation in Microsoft excel^®^ (P < 0.05) using the log_2_fold values obtained from both data series.

## Supplementary information

Zuniga-Soto et al Suppl Material

## Data Availability

BAM file alignments for the 36 libraries were deposited in the Sequence Read Archives (SRA) of NCBI under accession number BioProject PRJNA479665 (https://www.ncbi.nlm.nih.gov/bioproject/?term=PRJNA479665). An Excel file including all the CuffDiff results has been included (Supplementary Table 7).
