# Supplementary material for: Insights into the transcriptomic response of the plant engineering bacterium Ensifer adhaerens OV14 during transformation
Source: Sci Rep. 2019 Jul 17;9:10344. doi: 10.1038/s41598-019-44648-8 (PMC6637203; doi:10.1038/s41598-019-44648-8)
Supplement: Supplementary file 1 — Zuniga-Soto et al Suppl Material [file 41598_2019_44648_MOESM1_ESM.docx]

**Supplementary Materials**

**Insights into the transcriptomic response of the plant engineering bacterium *Ensifer adhaerens* OV14 during transformation**

Evelyn Zuniga-Soto^1,3^, David A. Fitzpatrick^2^, Fiona M. Doohan^3^, Ewen Mullins^1*^

^1^Department of Crop Science, Teagasc Crops Research Centre, Oak Park, Carlow, Ireland

^2^Department of Biology, National University of Ireland Maynooth

^3.^School of Biology and Environmental Sciences, University College Dublin, Belfield, Dublin 4, Ireland

**Keywords**

*Ensifer adhaerens* OV14, RNAseq, plant-bacteria interaction, signalling, acetosyringone, gene expression, *A. thaliana*.

*corresponding author: [ewen.mullins@teagasc.ie](mailto:ewen.mullins@teagasc.ie)

**Abstract**

The ability to engineer plant genomes has been primarily driven by the soil bacterium *Agrobacterium tumefaciens*but recently the potential of alternative rhizobia such as *Rhizobium etli*and *Ensifer adhaerens*OV14, which supports *Ensifer* Mediated TransformatIon (EMT) has been reported. Surprisingly, a knowledge deficit exists in regards to understanding the whole genome processes underway in plant transforming bacteria, irrespective of the species. To begin to address the issue, we undertook a temporal RNAseq-based profiling study of *E. adhaerens* OV14 in the presence/absence of *Arabidopsis thaliana* tissues. Following co-cultivation with root tissues, 2333 differentially expressed genes (DEGs) were noted. Meta-analysis of the RNAseq data sets identified a clear shift from plasmid-derived gene expression to chromosomal-based transcription within the early stages of bacterium-plant co-cultivation. During this time, the number of differentially expressed prokaryotic genes increased steadily out to 7 days co-cultivation, a time at which optimum rates of transformation were observed. Gene ontology evaluations indicated a role for both chromosomal and plasmid-based gene families linked specifically with quorum sensing, flagellin production and biofilm formation in the process of EMT. Transcriptional evaluation of *vir* genes, housed on the pCAMBIA 5105 plasmid in *E. adhaerens* OV14 confirmed the ability of *E. adhaerens* OV14 to perceive and activate its transcriptome in response to the presence of 200 µM of acetosyringone. Significantly, this is the first study to characterise the whole transcriptomic response of a plant engineering bacterium in the presence of plant tissues and provides a novel insight into prokaryotic genetic processes that support T-DNA transfer.

| **a.** | **b.** |
| --- | --- |
| **c.** | **d.** |
| **e.** | **f.** |
| **g.** | **h.** |
| **i.** |  |

**Supplementary Figure S1. Comparative plots of log_2_fold change expression derived from RNAseq and qRT-PCR analysis for each gene.** Log_2_fold change plots between BR and B conditions for RNAseq and qRT-PCR for the genes **(a)** *virB1*, **(b)** *virK*, **(c)** *OV14_RS30665*, **(d)** *OV14_RS18490*, **(e)** *OV14_RS22505*, **(f)** OV14_RS31975, **(g)** OV14_RS30910, **(h)** OV14_RS16760 (*chvD*) and **(i)** *trbD*.

| 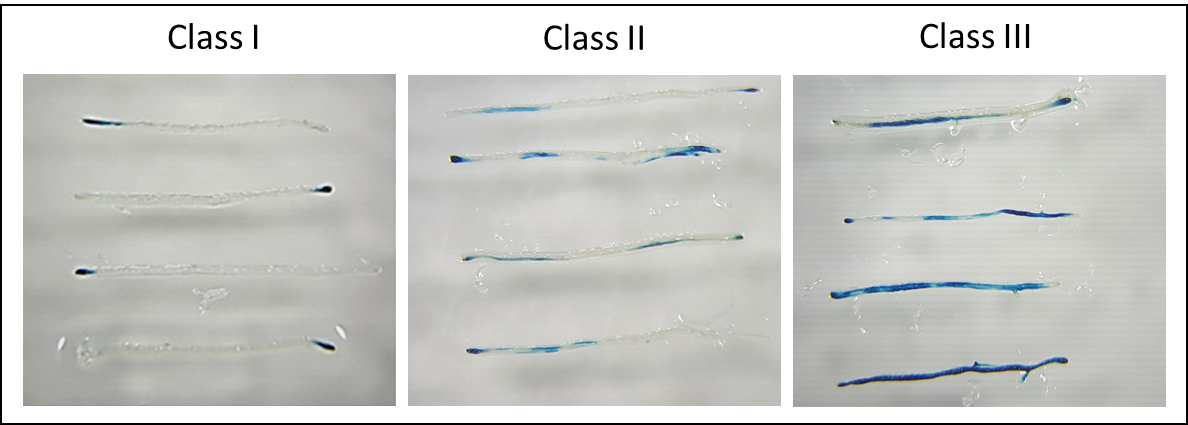 |
| --- |

**Supplementary Figure S2.** Illustration of different classes of blue foci counts described in materials and methods. Class I corresponded to the count of roots with a single foci; class II corresponded to the count of roots wth < 50% of surface area stained blue and class III corresponded to roots with approximately >50% and <100% of roots stained. Class T (not ilustrated here) corresponded to the count presence or absence of blue foci (regardless of the size of the foci).


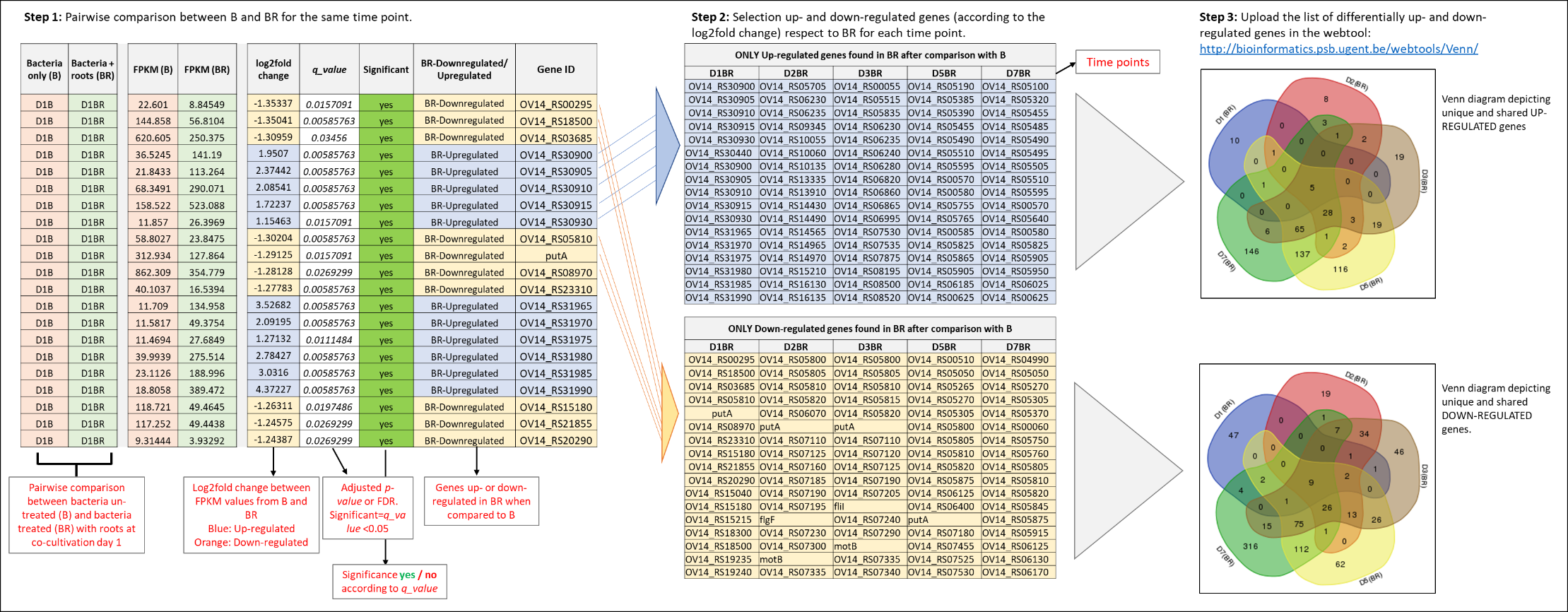


**Supplementary Figure S3.** Identification of differentially expressed genes (DEG) used to construct Venn diagrams collapsing all the timepoints evaluated. In the first step **(step 1)** DEGs were selected by making a pairwise comparison between bacteria un-treated (B) and treated (BR) with *A. thaliana* roots, identifying the genes up-regulated (blue highlighted) and down-regulated (yellow background) genes for BR. The second step **(step 2)** involved the selection of up- and down-regulated genes in two separate tables showing which genes are differentially expressed at each specific time point. Finally, the third step **(step 3)** consisted of uploading the separate list of up-and down-regulated genes in order to obtain the Venn diagrams that show how many and genes are exclusively present or shared between different time points. After obtaining the Venn diagrams, the webtool <http://bioinformatics.psb.ugent.be/webtools/Venn/> was used to provide the list of genes corresponding to each time point.


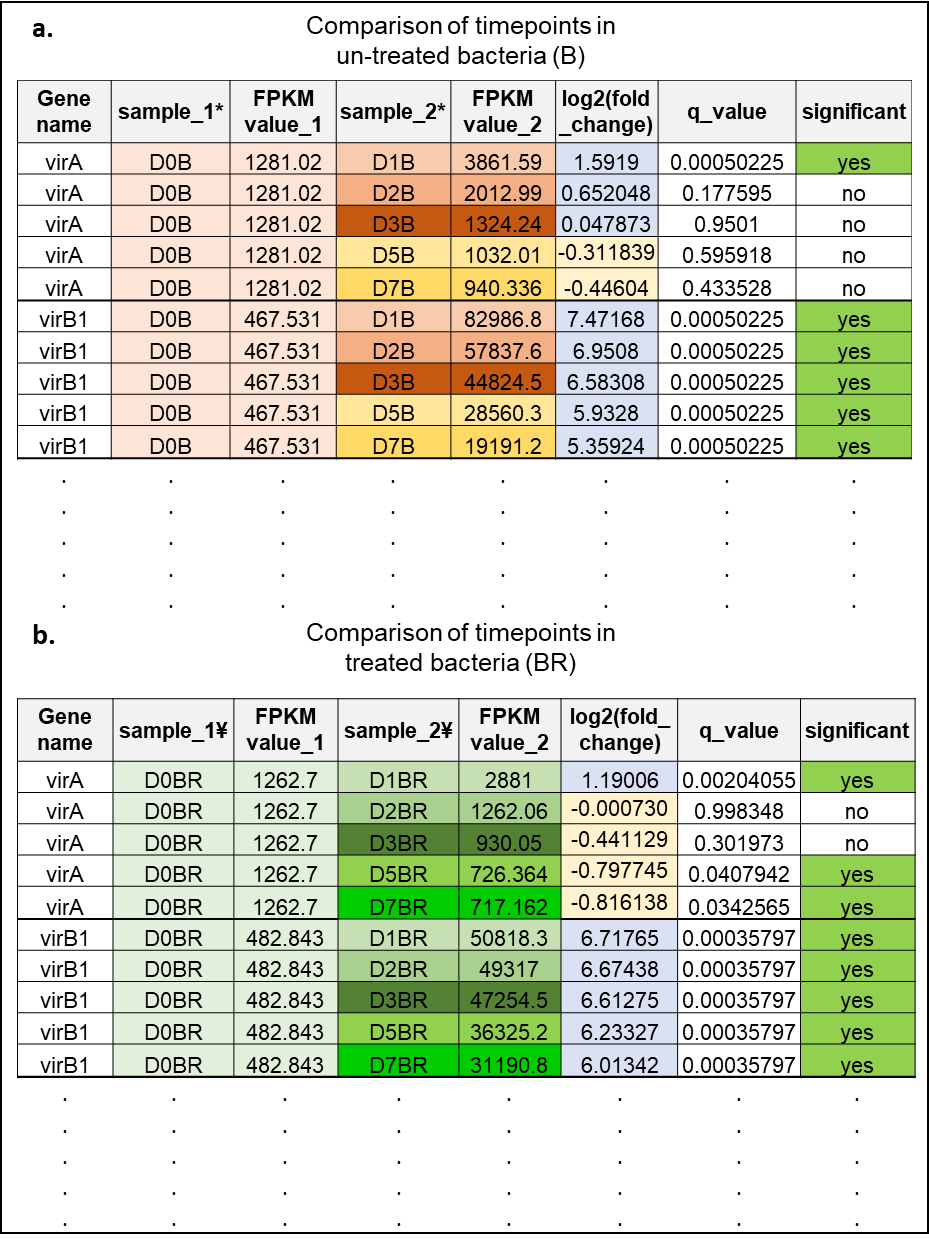


**Supplementary Figure S4.** Independent pairwise comparison in **(a)** un-treated and **(b)** treated bacteria across the different co-cultivation timepoints for the *virA* and virB1 (this pairwise analysis was conducted for the complete set of *vir* genes). Asterisks (*) in 2^nd^ and 4^th^ columns in **(a)** indicate that the samples corresponded to un-treated bacteria (B); the symbol (¥) in 2^nd^ and 4^th^ columns in **(b)** indicate that samples corresponded to treated bacteria (BR). In both tables the FPKM values located in column 3^rd^ correspond to co-cultivation timepoint day 0 (D0) and the values in column 5^th^ correspond to FPKM values from co-cultivation timepoints day 1 (D1), day 2 (D2), day 3 (D3), day 5 (D5) and day 7 (D7). Significance of the comparison is depicted in the last column of each table.

| **a.**   | |
| --- | --- |
| …continue…   | |
| **b.**  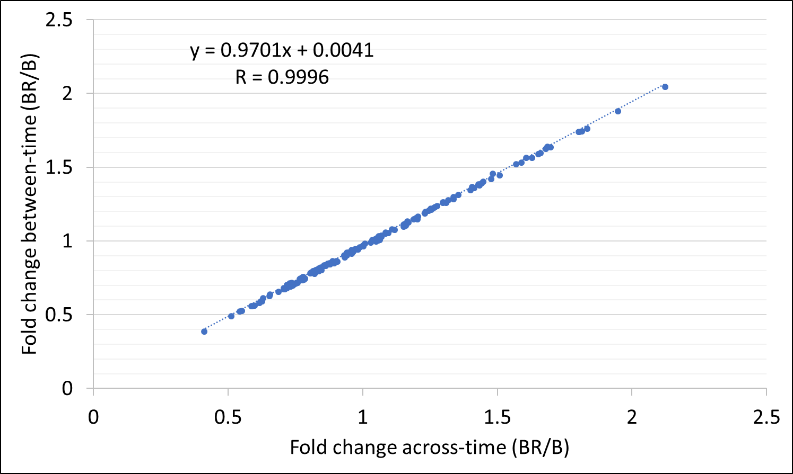 |  |

**Supplementary Figure S5.** **(a)** Pearson correlation coefficient (r=0.99) observed in the 5^th^ column of each dataset between FPKM values obtained from *vir* genes evaluated across timepoints (AC) and between timepoints (BTW) for un-treated (B) and treated bacteria (BR). **(b)** Graph showing the correlation between the fold change values obtained after dividing the FPKM values from treated (BR) and un-treated (B) bacteria from evaluations across (AC) and between (BTW) timepoints.

| **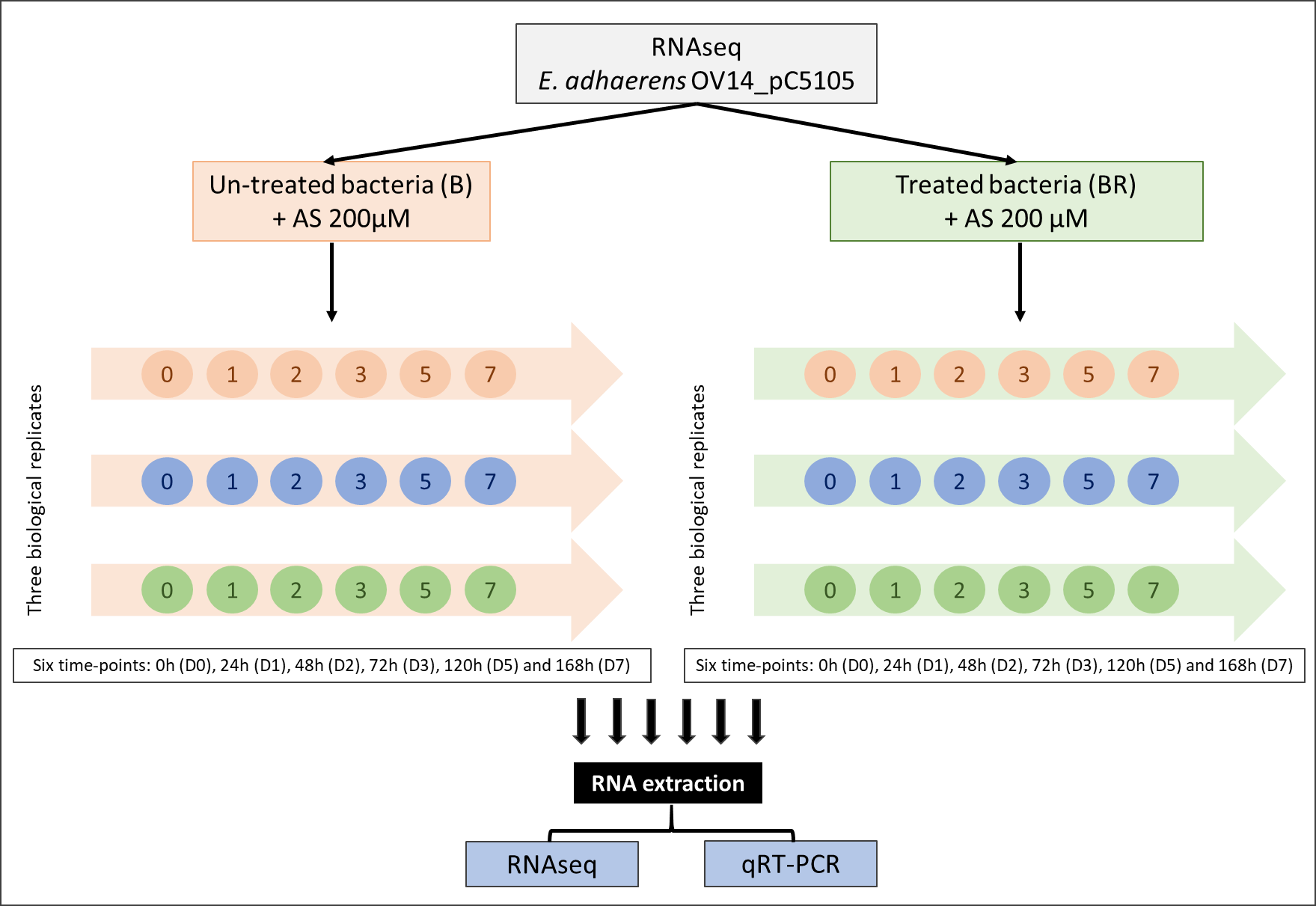**  **Supplementary Figure S6. Experimental design for RNAseq**. Bacterial strain *E. adhaerens* OV14_pC5105 un-treated (control) and treated with *A. thaliana* roots was cultured for up to seven days in co-cultivation media supplemented with acetosyringone 200 µM. There were three biological replicates for each condition. Samples for RNA extraction were taken at six timepoints: D0, D1, D2, D3, D5 and D7, after which RNA was used for RNAseq and qRT-PCR. |
| --- |

| **RNAseq** | | | | | | **qRT-PCR** |
| --- | --- | --- | --- | --- | --- | --- |
| **a.** | | | | | |  |
| **b.** | | | | | |  |
| **c.** | | | | |  | |
| **d.** | | | | | |  |
| **e.** |  | | | | | |
| **f.** | | |  | | | |
| **g.** | |  | | | | |
| **h.** | |  | | | | |
| **i.** | | | |  | | |

**Supplementary Figure S7. RNAseq profile obtained for 9 genes (left column) and corresponding profile obtained using qRT-PCR (right column).** In each graph, the red bars correspond to untreated bacteria (B) and the green bars correspond to bacteria treated with roots (BR) across 7 timepoints (D0, D1, D2, D3, D5 and D7). In the first column of this figure are the graphs corresponding FPKM values obtained using RNAseq and in the second column are the graphs obtained using qRT-PCR. Asterisks indicate evidence of significant differential expression using Tophat2 v2.1.1 (P<0.05) between EOV14_5105 un-treated (B) and treated (BR) with roots for three biological replicates. **a.** *virB* and **b.** *virK* are virulence genes located in the plasmid pCAMBIA5105. The genes **c.** OV14_RS18490 and **d.** OV14_RS16760 are located on chromosome 1, **e.** OV14_RS22505 is located on chromosome 2, **f.** OV14_RS28805 is located on plasmid c (pOV14c) and **g.** OV14_RS30665, **h.** OV14_RS31975 and **i.** OV14_RS30910 are located on plasmid b (pOV14b).

| **a.** | **b.** |
| --- | --- |
| **c.** |  |

**Supplementary Figure S8. Temporal FPKM expression values of genes *livF*, *livH* and *livM* differentially expressed and related to quorum sensing**. D0 to D7 indicate abbreviation for co-cultivation days described in the materials and methods section. Asterisks indicate evidence of significant differential expression using Tophat2 v2.1.1 (P<0.05) between EOV14_5105 un-treated (B) and treated (BR) with roots for three biological replicates. Red and green bars indicate the gene expression level for EOV14_5105 untreated (B) and treated (BR) with roots respectively. **a.** *livF*, **b.** *livH* and **c.** *livM*

| **a.** | **b.** |
| --- | --- |
| **c.** |  |
| **e.**  **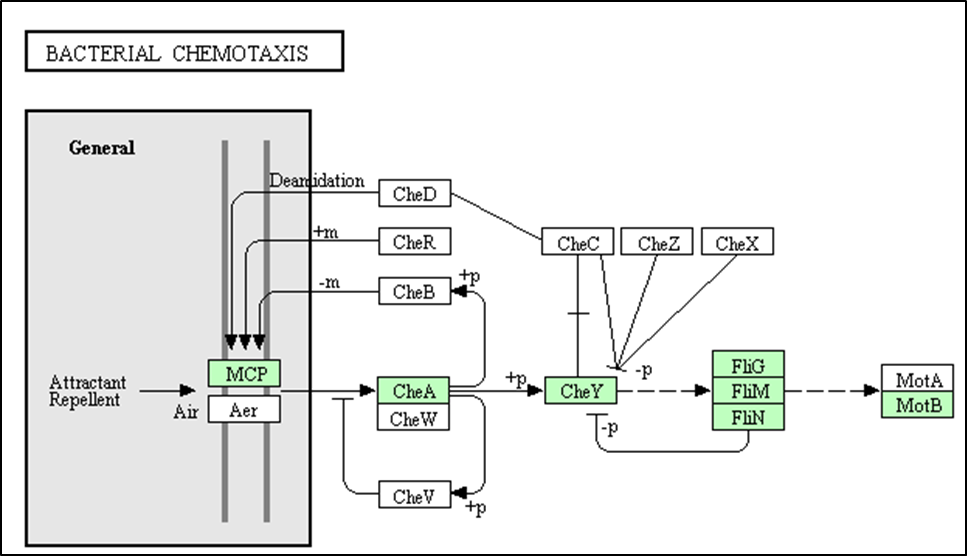** | |

**Supplementary Figure S9. Temporal FPKM expression values of genes *fliM*, *fliNY* and *cheY* differentially expressed and related to bacterial chemotaxis**. D0 to D7 indicate abbreviation for co-cultivation days described in the materials and methods section. Asterisks indicate evidence of significant differential expression using Tophat2 v2.1.1 (P<0.05) between EOV14_5105 un-treated (B) and treated (BR) with roots for three biological replicates. Red and green bars indicate the gene expression level for EOV14_5105 untreated (B) and treated (BR) with roots respectively. **a.** *fliM*, **b.** *fliNY*/*fliN* and **c.** *cheY;* **e.** metabolic KEGG pathway related to bacterial chemotaxis. Highlighted in green are the genes annotated in the KEGG database^116-118^ found to be differentally expressed in bacteria treated with roots (<https://www.kegg.jp/kegg/kegg1.html>).

116. Kanehisa, M., Sato, Y., Furumichi, M., Morishima, K., and Tanabe, M.; New approach for understanding genome variations in KEGG. Nucleic Acids Res. 47, D590-D595 (2019).

117. Kanehisa, Furumichi, M., Tanabe, M., Sato, Y., and Morishima, K.; KEGG: new perspectives on genomes, pathways, diseases and drugs. Nucleic Acids Res. 45, D353-D361 (2017).

118. Kanehisa, M. and Goto, S.; KEGG: Kyoto Encyclopedia of Genes and Genomes. Nucleic Acids Res. 28, 27-30 (2000).

| **a.** | **b.** |
| --- | --- |
| **c.** | **d.** |
| **e.** | **f.** |
| **g.** | **h.** |

| **i.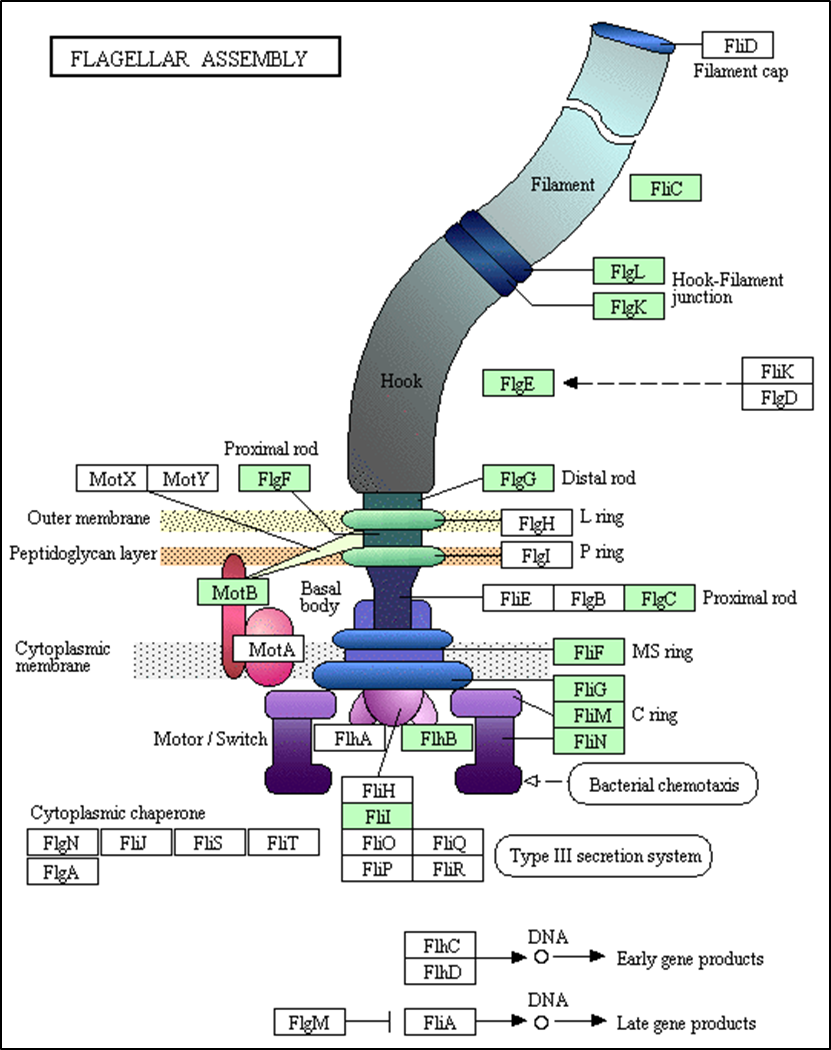** |
| --- |

**Supplementary Figure S10. Temporal FPKM expression values of genes *flgC, flgF, flgG, flgK, flgL, fliC, fliF* and *fliL* differentially expressed and related to flagellar assembly**. Expression levels are indicated as FPKM index (y-axis) and the period of co-cultivation in days (x-axis). D0 to D7 indicate abbreviation for co-cultivation days described in materials and methods section. Asterisks indicate evidence of significant differential expression using Tophat2 v2.1.1 (P<0.05) between EOV14_5105 un-treated (B) and treated (BR) with roots for three biological replicates. Red and green bars indicate the gene expression level for EOV14_5105 un-treated (B) and treated (BR) with roots respectively. **a.** *flgC*, **b.** *flgF*, **c.** *flgG*, **d.** *flgK*, **e.** *flgL*, **f.** *fliC*, **g.** *fliF* and **h.** *fliL*; **i.** metabolic KEGG pathway related to flagellar assembly. Highlighted in green are the genes annotated in the KEGG database^116-118^ found to be differentally expressed in bacteria treated with roots (<https://www.kegg.jp/kegg/kegg1.html>).

116. Kanehisa, M., Sato, Y., Furumichi, M., Morishima, K., and Tanabe, M.; New approach for understanding genome variations in KEGG. Nucleic Acids Res. 47, D590-D595 (2019).

117. Kanehisa, Furumichi, M., Tanabe, M., Sato, Y., and Morishima, K.; KEGG: new perspectives on genomes, pathways, diseases and drugs. Nucleic Acids Res. 45, D353-D361 (2017).

118. Kanehisa, M. and Goto, S.; KEGG: Kyoto Encyclopedia of Genes and Genomes. Nucleic Acids Res. 28, 27-30 (2000).

**Supplementary Table S1.** Total number of clean reads mapped to the reference genome from *E. adhaerens* and the plasmid pCAMBIA5105.

**Supplementary Table S2** Complete list of up- and down-regulated genes expressed exclusively in single timepoints or shared between timepoints associated with the Venn diagrams from Figure 4. See also Supplementary Table S7.

**Up-regulated genes**

| **Day(s)** | **No. genes** | | **Location** | **Gene ID** | | **Protein ID** | | | **NCBI annotated function** | | | |  |
| --- | --- | --- | --- | --- | --- | --- | --- | --- | --- | --- | --- | --- | --- |
| D1BR (10) | 10 | | chromosome 1 | OV14_RS15455 | | WP_025427501.1 | | | >WP_025427501.1 hypothetical protein [Ensifer adhaerens] | | | |  |
|  |  | | plasmid b | OV14_RS30900 | | WP_025430464.1 | | | >WP_025430464.1 hypothetical protein [Ensifer adhaerens] | | | |  |
|  |  | | plasmid b | OV14_RS30905 | | WP_025430465.1 | | | >WP_025430465.1 hypothetical protein [Ensifer adhaerens] | | | |  |
|  |  | | plasmid b | OV14_RS30910 | | WP_025430466.1 | | | >WP_025430466.1 hypothetical protein [Ensifer adhaerens] | | | |  |
|  |  | | plasmid b | OV14_RS30915 | | WP_025430467.1 | | | >WP_025430467.1 hypothetical protein [Ensifer adhaerens] | | | |  |
|  |  | | plasmid b | OV14_RS30930 | | WP_041692924.1 | | | >WP_041692924.1 hypothetical protein [Ensifer adhaerens] | | | |  |
|  |  | | plasmid b | OV14_RS31965 | | WP_025430672.1 | | | >WP_025430672.1 haloperoxidase [Ensifer adhaerens] | | | |  |
|  |  | | plasmid b | OV14_RS31970 | | WP_025430673.1 | | | >WP_025430673.1 pyridine nucleotide-disulfide oxidoreductase [Ensifer adhaerens] | | | |  |
|  |  | | plasmid b | OV14_RS31975 | | WP_025430674.1 | | | >WP_025430674.1 membrane protein [Ensifer adhaerens] | | | |  |
|  |  | | plasmid b | OV14_RS31980 | | WP_025430675.1 | | | >WP_025430675.1 cupin [Ensifer adhaerens] | | | |  |
| D2BR (8) | 8 | | chromosome 1 | OV14_RS02610 | | WP_025425028.1 | | | >WP_025425028.1 MULTISPECIES: arginase [Ensifer] | | | |  |
|  |  | | chromosome 1 | OV14_RS05705 | | WP_025425623.1 | | | >WP_025425623.1 MULTISPECIES: hypothetical protein [Ensifer] | | | |  |
|  |  | | chromosome 1 | OV14_RS13335 | | WP_038576103.1 | | | >WP_038576103.1 MULTISPECIES: hypothetical protein [Ensifer] | | | |  |
|  |  | | chromosome 1 | OV14_RS14565 | | WP_025427323.1 | | | >WP_025427323.1 MULTISPECIES: membrane protein [Ensifer] | | | |  |
|  |  | | chromosome 1 | OV14_RS16130 | | WP_025427630.1 | | | >WP_025427630.1 MULTISPECIES: transcriptional regulator [Ensifer] | | | |  |
|  |  | | chromosome 1 | OV14_RS16685 | | WP_025427738.1 | | | >WP_025427738.1 MULTISPECIES: lytic transglycosylase [Ensifer] | | | |  |
|  |  | | chromosome 2 | OV14_RS26340 | | WP_025429590.1 | | | >WP_025429590.1 hypothetical protein [Ensifer adhaerens] | | | |  |
|  |  | | chromosome 2 | OV14_RS26515 | | WP_025429624.1 | | | >WP_025429624.1 MULTISPECIES: hypothetical protein [Ensifer] | | | |  |
| D3BR (19) | 19 | | chromosome 1 | OV14_RS00040 | | WP_025424542.1 | | | >WP_025424542.1 MULTISPECIES: tRNA (N6-threonylcarbamoyladenosine(37)-N6)-methyltransferase TrmO [Ensifer] | | | |  |
|  |  | | chromosome 1 | OV14_RS00055 | | WP_025424545.1 | | | >WP_025424545.1 MULTISPECIES: iron ABC transporter substrate-binding protein [Ensifer] | | | |  |
|  |  | | chromosome 1 | OV14_RS02115 | | WP_038576520.1 | | | >WP_038576520.1 MULTISPECIES: molybdate ABC transporter substrate-binding protein [Ensifer] | | | |  |
|  |  | | chromosome 1 | OV14_RS02850 | | WP_025425074.1 | | | >WP_025425074.1 MULTISPECIES: polysaccharide biosynthesis protein GumN [Ensifer] | | | |  |
|  |  | | chromosome 1 | OV14_RS03300 | | WP_025425159.1 | | | >WP_025425159.1 MULTISPECIES: exodeoxyribonuclease III [Ensifer] | | | |  |
|  |  | | chromosome 1 | OV14_RS05515 | | WP_025425585.1 | | | >WP_025425585.1 MULTISPECIES: aldehyde-activating protein [Ensifer] | | | |  |
|  |  | | chromosome 1 | OV14_RS05835 | | WP_025425648.1 | | | >WP_025425648.1 MULTISPECIES: hypothetical protein [Ensifer] | | | |  |
|  |  | | chromosome 1 | OV14_RS06820 | | WP_025425840.1 | | | >WP_025425840.1 MULTISPECIES: hypothetical protein [Ensifer] | | | |  |
|  |  | | chromosome 1 | OV14_RS07530 | | WP_025425977.1 | | | >WP_025425977.1 MULTISPECIES: hypothetical protein [Ensifer] | | | |  |
|  |  | | chromosome 1 | OV14_RS07535 | | WP_025425978.1 | | | >WP_025425978.1 MULTISPECIES: isocitrate lyase [Ensifer] | | | |  |
|  |  | | chromosome 1 | OV14_RS07875 | | WP_025426044.1 | | | >WP_025426044.1 MULTISPECIES: membrane protein [Ensifer] | | | |  |
|  |  | | chromosome 1 | OV14_RS08595 | | WP_063963380.1 | | | >WP_063963380.1 MULTISPECIES: hypothetical protein [Ensifer] | | | |  |
|  |  | | chromosome 1 | OV14_RS09120 | | WP_025426286.1 | | | >WP_025426286.1 MULTISPECIES: MerR family transcriptional regulator [Ensifer] | | | |  |
|  |  | | chromosome 1 | OV14_RS17615 | | WP_025427915.1 | | | >WP_025427915.1 MULTISPECIES: protein-tyrosine-phosphatase [Ensifer] | | | |  |
|  |  | | chromosome 2 | OV14_RS22505 | | WP_025428859.1 | | | >WP_025428859.1 exopolysaccharide production repressor exox [Ensifer adhaerens] | | | |  |
|  |  | | chromosome 2 | OV14_RS23870 | | WP_025429114.1 | | | >WP_025429114.1 MULTISPECIES: transcriptional regulator [Ensifer] | | | |  |
|  |  | | chromosome 2 | OV14_RS25155 | | WP_025429364.1 | | | >WP_025429364.1 MULTISPECIES: endoribonuclease L-PSP [Ensifer] | | | |  |
|  |  | | chromosome 2 | OV14_RS26035 | | WP_003525288.1 | | | Pseudogen | | | |  |
|  |  | | chromosome 2 | OV14_RS26445 | | WP_025429610.1 | | | >WP_025429610.1 MULTISPECIES: hypothetical protein [Ensifer] | | | |  |
| D5BR (116) | | 116 | chromosome 1 | OV14_RS00375 | | WP_025424607.1 | | hypothetical protein [Ensifer adhaerens] | | | |  |  |
|  | |  | chromosome 1 | OV14_RS00450 | | WP_025424620.1 | | MULTISPECIES: disulfide bond formation protein | | | |  |  |
|  | |  | chromosome 1 | OV14_RS00585 | | WP_025424645.1 | | MULTISPECIES: hypothetical protein [Ensifer] | | | |  |  |
|  | |  | chromosome 1 | OV14_RS00730 | | WP_025424674.1 | | MULTISPECIES: ArsR family transcriptional regulator | | | |  |  |
|  | |  | chromosome 1 | OV14_RS00820 | | WP_025424692.1 | | MULTISPECIES: histidine ABC transporter substrate-binding | | | |  |  |
|  | |  | chromosome 1 | OV14_RS01085 | | WP_025424742.1 | | MULTISPECIES: Holliday junction ATP-dependent DNA | | | |  |  |
|  | |  | chromosome 1 | OV14_RS01275 | | WP_025424779.1 | | MULTISPECIES: inositol monophosphatase [Ensifer] | | | |  |  |
|  | |  | chromosome 1 | OV14_RS01380 | | WP_025424799.1 | | oligoendopeptidase F [Ensifer adhaerens] | | | |  |  |
|  | |  | chromosome 1 | OV14_RS01865 | | WP_025424891.1 | | MULTISPECIES: protein YebE [Ensifer] | | | |  |  |
|  | |  | chromosome 1 | OV14_RS01895 | | WP_025424897.1 | | MULTISPECIES: dioxygenase [Ensifer] | | | |  |  |
|  | |  | chromosome 1 | OV14_RS02160 | | WP_025424948.1 | | MULTISPECIES: acetyl-CoA acetyltransferase [Ensifer] | | | |  |  |
|  | |  | chromosome 1 | OV14_RS02395 | | WP_025424988.1 | | MULTISPECIES: polyphosphate kinase [Ensifer] | | | |  |  |
|  | |  | chromosome 1 | OV14_RS02520 | | WP_051509186.1 | | MULTISPECIES: IclR family transcriptional regulator | | | |  |  |
|  | |  | chromosome 1 | OV14_RS02540 | | WP_025425014.1 | | MULTISPECIES: hypothetical protein [Ensifer] | | | |  |  |
|  | |  | chromosome 1 | OV14_RS02545 | | WP_025425015.1 | | MULTISPECIES: 1-deoxy-D-xylulose-5-phosphate reductoisomerase [Ensifer] | | | |  |  |
|  | |  | chromosome 1 | OV14_RS02550 | | WP_025425016.1 | | MULTISPECIES: 5-aminolevulinate synthase [Ensifer] | | | |  |  |
|  | |  | chromosome 1 | OV14_RS02860 | | WP_025425076.1 | | MULTISPECIES: dihydrolipoyl dehydrogenase [Ensifer] | | | |  |  |
|  | |  | chromosome 1 | OV14_RS02885 | | WP_025425081.1 | | MULTISPECIES: hypothetical protein [Ensifer] | | | |  |  |
|  | |  | chromosome 1 | OV14_RS02890 | | WP_025425082.1 | | MULTISPECIES: dihydrolipoamide succinyltransferase [Ensifer] | | | |  |  |
|  | |  | chromosome 1 | OV14_RS03165 | | WP_038575756.1 | | MULTISPECIES: DNA polymerase [Ensifer] | | | |  |  |
|  | |  | chromosome 1 | OV14_RS03430 | | WP_025425185.1 | | MULTISPECIES: heme transporter HemC [Ensifer] | | | |  |  |
|  | |  | chromosome 1 | OV14_RS03685 | | WP_025425235.1 | | MULTISPECIES: amino acid ABC transporter | | | |  |  |
|  | |  | chromosome 1 | OV14_RS03735 | | WP_025425245.1 | | MULTISPECIES: DUF1338 domain-containing protein [Ensifer] | | | |  |  |
|  | |  | chromosome 1 | OV14_RS03745 | | WP_025425247.1 | | MULTISPECIES: aldehyde dehydrogenase [Ensifer] | | | |  |  |
|  | |  | chromosome 1 | OV14_RS03850 | | WP_025425268.1 | | MULTISPECIES: lysine transporter LysE [Ensifer] | | | |  |  |
|  | |  | chromosome 1 | OV14_RS04370 | | WP_025425370.1 | | MULTISPECIES: two-component sensor histidine kinase | | | |  |  |
|  | |  | chromosome 1 | OV14_RS04610 | | WP_025425412.1 | | MULTISPECIES: 2,3-bisphosphoglycerate-dependent phosphoglycerate mutase [Ensifer] | | | |  |  |
|  | |  | chromosome 1 | OV14_RS04745 | | WP_038576605.1 | | MULTISPECIES: hypothetical protein [Ensifer] | | | |  |  |
|  | |  | chromosome 1 | OV14_RS04920 | | WP_038575793.1 | | MULTISPECIES: cytochrome c [Ensifer] | | | |  |  |
|  | |  | chromosome 1 | OV14_RS05190 | | WP_025425523.1 | | MULTISPECIES: SelT/selW/selH selenoprotein [Ensifer] | | | |  |  |
|  | |  | chromosome 1 | OV14_RS05385 | | WP_025425561.1 | | MULTISPECIES: alcohol dehydrogenase [Ensifer] | | | |  |  |
|  | |  | chromosome 1 | OV14_RS05390 | | WP_025425562.1 | | MULTISPECIES: oxidoreductase [Ensifer] | | | |  |  |
|  | |  | chromosome 1 | OV14_RS05755 | | WP_025425632.1 | | MULTISPECIES: deoxyuridine 5'-triphosphate nucleotidohydrolase [Ensifer] | | | |  |  |
|  | |  | chromosome 1 | OV14_RS05765 | | WP_025425634.1 | | MULTISPECIES: hypothetical protein [Ensifer] | | | |  |  |
|  | |  | chromosome 1 | OV14_RS05865 | | WP_025425653.1 | | MULTISPECIES: hypothetical protein [Ensifer] | | | |  |  |
|  | |  | chromosome 1 | OV14_RS06185 | | WP_051509148.1 | | MULTISPECIES: nucleoside hydrolase [Ensifer] | | | |  |  |
|  | |  | chromosome 1 | OV14_RS06440 | | WP_025425766.1 | | MULTISPECIES: Crp/Fnr family transcriptional regulator | | | |  |  |
|  | |  | chromosome 1 | OV14_RS07010 | | WP_025425877.1 | | MULTISPECIES: arsenate reductase [Ensifer] | | | |  |  |
|  | |  | chromosome 1 | OV14_RS07950 | | WP_004675403.1 | | MULTISPECIES: molecular chaperone GroES [Rhizobiaceae] molecular chaperone GroES [Rhizobiaceae] | | | |  |  |
|  | |  | chromosome 1 | OV14_RS08095 | | WP_025426086.1 | | MULTISPECIES: gluconolaconase [Ensifer] | | | |  |  |
|  | |  | chromosome 1 | OV14_RS08180 | | WP_025426103.1 | | MULTISPECIES: ribonuclease HII [Ensifer] | | | |  |  |
|  | |  | chromosome 1 | OV14_RS08185 | | WP_025426104.1 | | MULTISPECIES: radical SAM protein [Ensifer] | | | |  |  |
|  | |  | chromosome 1 | OV14_RS08515 | | WP_025426169.1 | | MULTISPECIES: 3,4-dihydroxy-2-butanone-4-phosphate synthase [Ensifer] | | | |  |  |
|  | |  | chromosome 1 | OV14_RS08705 | | WP_025426206.1 | | MULTISPECIES: AraC family transcriptional regulator | | | |  |  |
|  | |  | chromosome 1 | OV14_RS08765 | | WP_025426217.1 | | MULTISPECIES: hypothetical protein [Ensifer] | | | |  |  |
|  | |  | chromosome 1 | OV14_RS36350 | | WP_025426252.1 | | MULTISPECIES: hypothetical protein [Ensifer] | | | |  |  |
|  | |  | chromosome 1 | OV14_RS09015 | | WP_025426265.1 | | MULTISPECIES: 2,5-didehydrogluconate reductase [Ensifer] | | | |  |  |
|  | |  | chromosome 1 | OV14_RS09040 | | WP_025426270.1 | | MULTISPECIES: phosphatase [Ensifer] | | | |  |  |
|  | |  | chromosome 1 | OV14_RS09055 | | WP_025426273.1 | | MULTISPECIES: aminotransferase [Ensifer] | | | |  |  |
|  | |  | chromosome 1 | OV14_RS09130 | | WP_025426288.1 | | MULTISPECIES: class A beta-lactamase [Ensifer] | | | |  |  |
|  | |  | chromosome 1 | OV14_RS09330 | | WP_025426328.1 | | MULTISPECIES: peptidoglycan-binding protein [Ensifer] | | | |  |  |
|  | |  | chromosome 1 | OV14_RS09560 | | WP_025426372.1 | | MULTISPECIES: histidinol-phosphate aminotransferase [Ensifer] | | | |  |  |
|  | |  | chromosome 1 | OV14_RS09850 | | WP_025426429.1 | | MULTISPECIES: Rossman fold protein, TIGR00730 | | | |  |  |
|  | |  | chromosome 1 | OV14_RS10150 | | WP_025426488.1 | | MULTISPECIES: hypothetical protein [Ensifer] | | | |  |  |
|  | |  | chromosome 1 | OV14_RS10195 | | WP_038575993.1 | | MULTISPECIES: AI-2E family transporter [Ensifer] | | | |  |  |
|  | |  | chromosome 1 | OV14_RS10770 | | WP_025426603.1 | | MULTISPECIES: hypothetical protein [Ensifer] | | | |  |  |
|  | |  | chromosome 1 | OV14_RS10955 | | WP_025426638.1 | | MULTISPECIES: 3-dehydroquinate dehydratase [Ensifer] | | | |  |  |
|  | |  | chromosome 1 | OV14_RS11910 | | WP_025426816.1 | | MULTISPECIES: endoribonuclease [Ensifer] | | | |  |  |
|  | |  | chromosome 1 | OV14_RS13390 | | WP_025427090.1 | | MULTISPECIES: alkene reductase [Ensifer] | | | |  |  |
|  | |  | chromosome 1 | OV14_RS13495 | | WP_025427110.1 | | MULTISPECIES: hypothetical protein [Ensifer] | | | |  |  |
|  | |  | chromosome 1 | OV14_RS13835 | | WP_025427178.1 | | MULTISPECIES: pseudouridine-5'-phosphate glycosidase [Ensifer] | | | |  |  |
|  | |  | chromosome 1 | OV14_RS13935 | | WP_025427197.1 | | MULTISPECIES: ubiquinol-cytochrome c reductase iron-sulfur | | | |  |  |
|  | |  | chromosome 1 | OV14_RS14185 | | WP_025427247.1 | | MULTISPECIES: pyridoxamine 5'-phosphate oxidase [Ensifer] | | | |  |  |
|  | |  | chromosome 1 | OV14_RS14400 | | WP_025427290.1 | | MULTISPECIES: ABC transporter substrate-binding protein | | | |  |  |
|  | |  | chromosome 1 | OV14_RS14435 | | WP_025427297.1 | | GNAT family N-acetyltransferase | | | |  |  |
|  | |  | chromosome 1 | OV14_RS14510 | | WP_025427312.1 | | hypothetical protein [Ensifer adhaerens] | | | |  |  |
|  | |  | chromosome 1 | OV14_RS14600 | | WP_025427330.1 | | MULTISPECIES: oxidoreductase [Ensifer] | | | |  |  |
|  | |  | chromosome 1 | OV14_RS14635 | | WP_025427337.1 | | MULTISPECIES: ABC transporter substrate-binding protein | | | |  |  |
|  | |  | chromosome 1 | OV14_RS14745 | | WP_025427358.1 | | MULTISPECIES: beta-ketoacyl-ACP synthase II [Ensifer] | | | |  |  |
|  | |  | chromosome 1 | OV14_RS15190 | | WP_025427448.1 | | MULTISPECIES: short-chain dehydrogenase/reductase [Ensifer] | | | |  |  |
|  | |  | chromosome 1 | OV14_RS15380 | | WP_025427486.1 | | MULTISPECIES: hypothetical protein [Ensifer] | | | |  |  |
|  | |  | chromosome 1 | OV14_RS15735 | | WP_025427552.1 | | MULTISPECIES: aldehyde oxidase [Ensifer] | | | |  |  |
|  | |  | chromosome 1 | OV14_RS15915 | | WP_025427587.1 | | MULTISPECIES: NUDIX hydrolase [Ensifer] | | | |  |  |
|  | |  | chromosome 1 | OV14_RS16110 | | WP_025427626.1 | | MULTISPECIES: ArsR family transcriptional regulator | | | |  |  |
|  | |  | chromosome 1 | OV14_RS16805 | | WP_025427761.1 | | MULTISPECIES: FAD-dependent oxidoreductase [Ensifer] | | | |  |  |
|  | |  | chromosome 1 | OV14_RS17135 | | WP_025427826.1 | | MULTISPECIES: hypothetical protein [Ensifer] | | | |  |  |
|  | |  | chromosome 1 | OV14_RS17890 | | WP_025427969.1 | | MULTISPECIES: protein hupE [Ensifer] | | | |  |  |
|  | |  | chromosome 1 | OV14_RS18085 | | WP_025428008.1 | | MULTISPECIES: glycerol kinase [Ensifer] | | | |  |  |
|  | |  | chromosome 1 | OV14_RS18105 | | WP_025428012.1 | | MULTISPECIES: hypothetical protein [Ensifer] | | | |  |  |
|  | |  | chromosome 1 | OV14_RS18260 | | WP_025428041.1 | | aldehyde dehydrogenase [Ensifer adhaerens] | | | |  |  |
|  | |  | chromosome 1 | OV14_RS18510 | | WP_025428089.1 | | MULTISPECIES: hemin-degrading factor [Ensifer] | | | |  |  |
|  | |  | chromosome 1 | OV14_RS18850 | | WP_025428157.1 | | MULTISPECIES: short-chain dehydrogenase [Ensifer] | | | |  |  |
|  | |  | chromosome 1 | OV14_RS19155 | | WP_025428212.1 | | MULTISPECIES: saccharopine dehydrogenase [Ensifer] | | | |  |  |
|  | |  | chromosome 2 | OV14_RS19245 | | WP_025428230.1 | | MULTISPECIES: glyoxalase [Ensifer] | | | |  |  |
|  | |  | chromosome 2 | OV14_RS20240 | | WP_025428418.1 | | beta-N-acetylhexosaminidase [Ensifer adhaerens] | | | |  |  |
|  | |  | chromosome 2 | OV14_RS20405 | | WP_025428451.1 | | MULTISPECIES: gluconokinase [Ensifer] | | | |  |  |
|  | |  | chromosome 2 | OV14_RS21535 | | WP_025428670.1 | | MULTISPECIES: hypothetical protein [Ensifer] | | | |  |  |
|  | |  | chromosome 2 | OV14_RS21800 | | WP_025428720.1 | | taurine ABC transporter substrate-binding protein | | | |  |  |
|  | |  | chromosome 2 | OV14_RS21835 | | WP_025428727.1 | | alkyl hydroperoxide reductase [Ensifer adhaerens] | | | |  |  |
|  | |  | chromosome 2 | OV14_RS22275 | | WP_025428813.1 | | MxaD family protein [Ensifer adhaerens] | | | |  |  |
|  | |  | chromosome 2 | OV14_RS22450 | | WP_025428848.1 | | MULTISPECIES: CMD domain protein [Ensifer] | | | |  |  |
|  | |  | chromosome 2 | OV14_RS22475 | | WP_025428853.1 | | ABC transporter substrate binding protein | | | |  |  |
|  | |  | chromosome 2 | OV14_RS22480 | | WP_025428854.1 | | UDP-glucose 4-epimerase GalE [Ensifer adhaerens] | | | |  |  |
|  | |  | chromosome 2 | OV14_RS23730 | | WP_025429086.1 | | 4-hydroxythreonine-4-phosphate dehydrogenase [Ensifer adhaerens] | | | |  |  |
|  | |  | chromosome 2 | OV14_RS24230 | | WP_025429186.1 | | molybdopterin-binding protein [Ensifer adhaerens] | | | |  |  |
|  | |  | chromosome 2 | OV14_RS24310 | | WP_025429200.1 | | MULTISPECIES: ABC transporter [Ensifer] | | | |  |  |
|  | |  | chromosome 2 | OV14_RS24650 | | WP_025429268.1 | | MULTISPECIES: hypothetical protein [Ensifer] | | | |  |  |
|  | |  | chromosome 2 | OV14_RS25435 | | WP_025429418.1 | | MULTISPECIES: hypothetical protein [Ensifer] | | | |  |  |
|  | |  | chromosome 2 | OV14_RS25560 | | WP_025429439.1 | | 2,5-dioxovalerate dehydrogenase [Ensifer adhaerens] | | | |  |  |
|  | |  | chromosome 2 | OV14_RS26000 | | WP_025429522.1 | | MFS transporter [Ensifer adhaerens] | | | |  |  |
|  | |  | chromosome 2 | OV14_RS26030 | | WP_025429528.1 | | MFS transporter [Ensifer adhaerens] | | | |  |  |
|  | |  | chromosome 2 | OV14_RS27630 | | WP_063991838.1 | | MULTISPECIES: hypothetical protein [Ensifer] | | | |  |  |
|  | |  | chromosome 2 | OV14_RS27735 | | WP_038578679.1 | | MULTISPECIES: LysR family transcriptional regulator | | | |  |  |
|  | |  | chromosome 2 | OV14_RS28265 | | WP_025429966.1 | | MULTISPECIES: dioxygenase [Ensifer] | | | |  |  |
|  | |  | plasmid b | OV14_RS28965 | | WP_025430088.1 | | MULTISPECIES: dienelactone hydrolase [Ensifer] | | | |  |  |
|  | |  | plasmid b | OV14_RS30090 | | WP_025430308.1 | | hydrolase [Ensifer adhaerens] | | | |  |  |
|  | |  | plasmid b | OV14_RS32525 | | WP_006201008.1 | | Pseudogene | | | |  |  |
|  | |  | plasmid b | OV14_RS32565 | | WP_025430786.1 | | PhnA protein [Ensifer adhaerens] | | | |  |  |
|  | |  | plasmid b | OV14_RS33370 | | WP_025430936.1 | | hypothetical protein [Ensifer adhaerens] | | | |  |  |
|  | |  | plasmid b | OV14_RS33535 | | WP_025430969.1 | | hypothetical protein [Ensifer adhaerens] | | | |  |  |
|  | |  | plasmid b | OV14_RS33580 | | WP_025430978.1 | | aldehyde dehydrogenase [Ensifer adhaerens] | | | |  |  |
|  | |  | plasmid b | OV14_RS33635 | | WP_025430989.1 | | hypothetical protein [Ensifer adhaerens] | | | |  |  |
|  | |  | plasmid b | OV14_RS33700 | | WP_003601684.1 | | Pseudogene | | | |  |  |
|  | |  | plasmid b | OV14_RS34700 | | WP_025431181.1 | | sugar ABC transporter permease [Ensifer | | | |  |  |
|  | |  | plasmid c | OV14_RS28500 | | WP_025430007.1 | | hypothetical protein [Ensifer adhaerens] | | | |  |  |
|  | |  | plasmid 5105 | virE2 | | [ABK01312.1](https://www.ncbi.nlm.nih.gov/protein/116585231) | | [type IV secretion system chaperone VirE1](https://www.ncbi.nlm.nih.gov/protein/116585231) | | | |  |  |
| D7BR (146) | 146 | | chromosome 1 | | OV14_RS00070 | | WP_025424548.1 | | | MULTISPECIES: riboflavin biosynthesis protein RibD [Ensifer] | | | |
|  |  | | chromosome 1 | | OV14_RS00130 | | WP_025424560.1 | | | MULTISPECIES: peptidylprolyl isomerase [Ensifer] | | | |
|  |  | | chromosome 1 | | OV14_RS00635 | | WP_025424655.1 | | | MULTISPECIES: hypothetical protein [Ensifer] | | | |
|  |  | | chromosome 1 | | OV14_RS00680 | | WP_025424664.1 | | | MULTISPECIES: site-specific tyrosine recombinase XerD [Ensifer] | | | |
|  |  | | chromosome 1 | | OV14_RS01155 | | WP_025424755.1 | | | MULTISPECIES: hypothetical protein [Ensifer] | | | |
|  |  | | chromosome 1 | | OV14_RS01365 | | WP_025424796.1 | | | MULTISPECIES: DUF2312 domain-containing protein [Ensifer] | | | |
|  |  | | chromosome 1 | | OV14_RS01690 | | WP_051509133.1 | | | MULTISPECIES: GlcNAc transferase [Ensifer] | | | |
|  |  | | chromosome 1 | | OV14_RS01980 | | WP_025424914.1 | | | MULTISPECIES: AraC family transcriptional regulator [Ensifer] | | | |
|  |  | | chromosome 1 | | OV14_RS02175 | | WP_025424951.1 | | | MULTISPECIES: 2-keto-4-pentenoate hydratase [Ensifer] | | | |
|  |  | | chromosome 1 | | OV14_RS03215 | | WP_025425144.1 | | | MULTISPECIES: ATPase AAA [Ensifer] | | | |
|  |  | | chromosome 1 | | OV14_RS03370 | | WP_025425173.1 | | | MULTISPECIES: ribosome maturation factor RimM [Ensifer] | | | |
|  |  | | chromosome 1 | | OV14_RS03395 | | WP_025425178.1 | | | MULTISPECIES: membrane protein [Ensifer] | | | |
|  |  | | chromosome 1 | | OV14_RS03530 | | WP_025425205.1 | | | MULTISPECIES: threonine transporter RhtB [Ensifer] | | | |
|  |  | | chromosome 1 | | OV14_RS03610 | | WP_025425220.1 | | | MULTISPECIES: beta-ketoacyl-ACP reductase [Ensifer] | | | |
|  |  | | chromosome 1 | | OV14_RS03785 | | WP_025425255.1 | | | MULTISPECIES: GMC family oxidoreductase [Ensifer] | | | |
|  |  | | chromosome 1 | | OV14_RS03955 | | WP_025425288.1 | | | MULTISPECIES: L-ectoine synthase [Ensifer] | | | |
|  |  | | chromosome 1 | | OV14_RS04390 | | WP_025425374.1 | | | MULTISPECIES: ABC transporter substrate-binding protein [Ensifer] | | | |
|  |  | | chromosome 1 | | OV14_RS04585 | | WP_025425407.1 | | | MULTISPECIES: penicillin-insensitive murein endopeptidase [Ensifer] | | | |
|  |  | | chromosome 1 | | OV14_RS04710 | | WP_025425432.1 | | | MULTISPECIES: penicillin-binding protein [Ensifer] | | | |
|  |  | | chromosome 1 | | OV14_RS04770 | | WP_025425444.1 | | | MULTISPECIES: TetR family transcriptional regulator [Ensifer] | | | |
|  |  | | chromosome 1 | | OV14_RS04960 | | WP_025425480.1 | | | MULTISPECIES: nucleoid-associated protein, YbaB/EbfC family [Ensifer] | | | |
|  |  | | chromosome 1 | | OV14_RS05100 | | WP_025425506.1 | | | MULTISPECIES: TIGR02300 family protein [Ensifer] | | | |
|  |  | | chromosome 1 | | OV14_RS05320 | | WP_038576631.1 | | | MULTISPECIES: translation initiation factor IF-3 [Ensifer] | | | |
|  |  | | chromosome 1 | | OV14_RS05485 | | WP_025425580.1 | | | MULTISPECIES: membrane protein [Ensifer] | | | |
|  |  | | chromosome 1 | | OV14_RS05495 | | WP_025425582.1 | | | MULTISPECIES: AsnC family transcriptional regulator [Ensifer] | | | |
|  |  | | chromosome 1 | | OV14_RS05505 | | WP_025425584.1 | | | MULTISPECIES: glutathione S-transferase [Ensifer] | | | |
|  |  | | chromosome 1 | | OV14_RS05640 | | WP_025425610.1 | | | MULTISPECIES: hypothetical protein [Ensifer] | | | |
|  |  | | chromosome 1 | | OV14_RS05950 | | WP_025425670.1 | | | MULTISPECIES: hypothetical protein [Ensifer] | | | |
|  |  | | chromosome 1 | | OV14_RS06025 | | WP_025425685.1 | | | MULTISPECIES: hypothetical protein [Ensifer] | | | |
|  |  | | chromosome 1 | | OV14_RS06245 | | WP_025425729.1 | | | MULTISPECIES: LysR family transcriptional regulator [Ensifer] | | | |
|  |  | | chromosome 1 | | OV14_RS06255 | | WP_025425731.1 | | | MULTISPECIES: QacE family quaternary ammonium compound efflux SMR transporter [Ensifer] | | | |
|  |  | | chromosome 1 | | OV14_RS06310 | | WP_025425742.1 | | | MULTISPECIES: acetylglutamate kinase [Ensifer] | | | |
|  |  | | chromosome 1 | | OV14_RS06455 | | WP_025425769.1 | | | MULTISPECIES: ROK family transcriptional regulator [Ensifer] | | | |
|  |  | | chromosome 1 | | OV14_RS06490 | | WP_025425776.1 | | | MULTISPECIES: carbohydrate kinase [Ensifer] | | | |
|  |  | | chromosome 1 | | OV14_RS06760 | | WP_038575860.1 | | | MULTISPECIES: riboflavin biosynthesis protein RibD [Ensifer] | | | |
|  |  | | chromosome 1 | | OV14_RS07170 | | WP_025425907.1 | | | MULTISPECIES: helix-turn-helix transcriptional regulator [Ensifer] | | | |
|  |  | | chromosome 1 | | OV14_RS07970 | | WP_025426061.1 | | | MULTISPECIES: hypothetical protein [Ensifer] | | | |
|  |  | | chromosome 1 | | OV14_RS08215 | | WP_025426110.1 | | | MULTISPECIES: hypothetical protein [Ensifer] | | | |
|  |  | | chromosome 1 | | OV14_RS08245 | | WP_025426116.1 | | | MULTISPECIES: hypothetical protein [Ensifer] | | | |
|  |  | | chromosome 1 | | OV14_RS08405 | | WP_025426148.1 | | | MULTISPECIES: hypothetical protein [Ensifer] | | | |
|  |  | | chromosome 1 | | OV14_RS08555 | | WP_025426177.1 | | | hypothetical protein [Ensifer adhaerens] | | | |
|  |  | | chromosome 1 | | OV14_RS08590 | | WP_025426184.1 | | | MULTISPECIES: peroxiredoxin [Ensifer] | | | |
|  |  | | chromosome 1 | | OV14_RS08675 | | WP_038575950.1 | | | MULTISPECIES: 2-haloalkanoic acid dehalogenase [Ensifer] | | | |
|  |  | | chromosome 1 | | OV14_RS08885 | | WP_025426240.1 | | | MULTISPECIES: hypothetical protein [Ensifer] | | | |
|  |  | | chromosome 1 | | OV14_RS08970 | | WP_025426257.1 | | | MULTISPECIES: organic hydroperoxide resistance protein [Ensifer] | | | |
|  |  | | chromosome 1 | | OV14_RS09200 | | WP_025426302.1 | | | MULTISPECIES: HxlR family transcriptional regulator [Ensifer] | | | |
|  |  | | chromosome 1 | | OV14_RS09235 | | WP_025426309.1 | | | MULTISPECIES: dimethylmenaquinone methyltransferase [Ensifer] | | | |
|  |  | | chromosome 1 | | OV14_RS09455 | | WP_025426353.1 | | | MULTISPECIES: aminopeptidase N [Ensifer] | | | |
|  |  | | chromosome 1 | | OV14_RS09495 | | WP_025426359.1 | | | MULTISPECIES: ABC transporter permease [Ensifer] | | | |
|  |  | | chromosome 1 | | OV14_RS09710 | | WP_025426401.1 | | | MULTISPECIES: phenylacetic acid degradation protein [Ensifer] | | | |
|  |  | | chromosome 1 | | OV14_RS09795 | | WP_025426418.1 | | | MULTISPECIES: TIGR02301 family protein [Ensifer] | | | |
|  |  | | chromosome 1 | | OV14_RS09900 | | WP_025426439.1 | | | MULTISPECIES: AraC family transcriptional regulator [Ensifer] | | | |
|  |  | | chromosome 1 | | OV14_RS09935 | | WP_025426446.1 | | | MULTISPECIES: 50S ribosomal protein L9 [Ensifer] | | | |
|  |  | | chromosome 1 | | OV14_RS10180 | | WP_038576849.1 | | | MULTISPECIES: type IV secretory pathway protein AcvB [Ensifer] | | | |
|  |  | | chromosome 1 | | OV14_RS10680 | | WP_025426585.1 | | | MULTISPECIES: NADH dehydrogenase [Ensifer] | | | |
|  |  | | chromosome 1 | | OV14_RS10685 | | WP_025426586.1 | | | MULTISPECIES: NADH-quinone oxidoreductase subunit F [Ensifer] | | | |
|  |  | | chromosome 1 | | OV14_RS10755 | | WP_025426600.1 | | | MULTISPECIES: proline--tRNA ligase [Ensifer] | | | |
|  |  | | chromosome 1 | | OV14_RS11165 | | WP_025426673.1 | | | MULTISPECIES: 30S ribosomal protein S17 [Ensifer] | | | |
|  |  | | chromosome 1 | | OV14_RS11185 | | WP_025426676.1 | | | MULTISPECIES: 30S ribosomal protein S14 [Ensifer] | | | |
|  |  | | chromosome 1 | | OV14_RS11480 | | WP_025426732.1 | | | MULTISPECIES: glycerol acyltransferase [Ensifer] | | | |
|  |  | | chromosome 1 | | OV14_RS11485 | | WP_025426733.1 | | | hypothetical protein [Ensifer adhaerens] | | | |
|  |  | | chromosome 1 | | OV14_RS11490 | | WP_025426734.1 | | | MULTISPECIES: nitroreductase [Ensifer] | | | |
|  |  | | chromosome 1 | | OV14_RS11755 | | WP_025426786.1 | | | MULTISPECIES: tRNA-specific adenosine deaminase [Ensifer] | | | |
|  |  | | chromosome 1 | | OV14_RS11820 | | WP_025426799.1 | | | MULTISPECIES: ABC transporter ATP-binding protein [Ensifer] | | | |
|  |  | | chromosome 1 | | OV14_RS11940 | | WP_025426822.1 | | | MULTISPECIES: di-trans,poly-cis-decaprenylcistransferase [Ensifer] | | | |
|  |  | | chromosome 1 | | OV14_RS11950 | | WP_025426824.1 | | | MULTISPECIES: zinc metalloprotease [Ensifer] | | | |
|  |  | | chromosome 1 | | OV14_RS12045 | | WP_025426843.1 | | | MULTISPECIES: exopolysaccharide production negative regulator [Ensifer] | | | |
|  |  | | chromosome 1 | | OV14_RS12165 | | WP_025426867.1 | | | MULTISPECIES: hypothetical protein [Ensifer] | | | |
|  |  | | chromosome 1 | | OV14_RS12195 | | WP_025426872.1 | | | MULTISPECIES: metal-dependent hydrolase [Ensifer] | | | |
|  |  | | chromosome 1 | | OV14_RS12990 | | WP_025427018.1 | | | MULTISPECIES: xanthine phosphoribosyltransferase [Ensifer] | | | |
|  |  | | chromosome 1 | | OV14_RS13005 | | WP_025427021.1 | | | MULTISPECIES: hypothetical protein [Ensifer] | | | |
|  |  | | chromosome 1 | | OV14_RS13010 | | WP_025427022.1 | | | MULTISPECIES: hypothetical protein [Ensifer] | | | |
|  |  | | chromosome 1 | | OV14_RS13080 | | WP_025427035.1 | | | MULTISPECIES: alanine dehydrogenase [Ensifer] | | | |
|  |  | | chromosome 1 | | OV14_RS13695 | | WP_025427150.1 | | | MULTISPECIES: hypothetical protein [Ensifer] | | | |
|  |  | | chromosome 1 | | OV14_RS13745 | | WP_025427160.1 | | | MULTISPECIES: monothiol glutaredoxin, Grx4 family [Ensifer] | | | |
|  |  | | chromosome 1 | | OV14_RS36385 | | WP_063963317.1 | | | MULTISPECIES: hypothetical protein [Ensifer] | | | |
|  |  | | chromosome 1 | | OV14_RS14590 | | WP_025427328.1 | | | MULTISPECIES: ABC transporter substrate-binding protein [Ensifer] | | | |
|  |  | | chromosome 1 | | OV14_RS14735 | | WP_025427356.1 | | | MULTISPECIES: NADPH:quinone oxidoreductase [Ensifer] | | | |
|  |  | | chromosome 1 | | OV14_RS15265 | | WP_038576197.1 | | | MULTISPECIES: cupin [Ensifer] | | | |
|  |  | | chromosome 1 | | OV14_RS15410 | | WP_025427492.1 | | | MULTISPECIES: hypothetical protein [Ensifer] | | | |
|  |  | | chromosome 1 | | OV14_RS15575 | | WP_038577166.1 | | | MULTISPECIES: nucleotidyltransferase [Ensifer] | | | |
|  |  | | chromosome 1 | | OV14_RS15720 | | WP_025427549.1 | | | MULTISPECIES: hypothetical protein [Ensifer] | | | |
|  |  | | chromosome 1 | | OV14_RS15840 | | WP_025427573.1 | | | MULTISPECIES: cold-shock protein [Ensifer] | | | |
|  |  | | chromosome 1 | | OV14_RS15950 | | WP_025427594.1 | | | MULTISPECIES: hypothetical protein [Ensifer] | | | |
|  |  | | chromosome 1 | | OV14_RS16385 | | WP_025427681.1 | | | MULTISPECIES: hypothetical protein [Ensifer] | | | |
|  |  | | chromosome 1 | | OV14_RS16440 | | WP_025427691.1 | | | MULTISPECIES: GNAT family acetyltransferase [Ensifer] | | | |
|  |  | | chromosome 1 | | OV14_RS16575 | | WP_025427716.1 | | | MULTISPECIES: outer membrane protein assembly factor BamD [Ensifer] | | | |
|  |  | | chromosome 1 | | OV14_RS16900 | | WP_025427780.1 | | | MULTISPECIES: erythrose-4-phosphate dehydrogenase [Ensifer] | | | |
|  |  | | chromosome 1 | | OV14_RS16955 | | WP_025427791.1 | | | MULTISPECIES: ABC transporter substrate-binding protein [Ensifer] | | | |
|  |  | | chromosome 1 | | OV14_RS17130 | | WP_025427825.1 | | | MULTISPECIES: aldehyde-activating protein [Ensifer] | | | |
|  |  | | chromosome 1 | | OV14_RS17365 | | WP_025427868.1 | | | MULTISPECIES: hypothetical protein [Ensifer] | | | |
|  |  | | chromosome 1 | | OV14_RS17940 | | WP_025427979.1 | | | MULTISPECIES: formate--tetrahydrofolate ligase [Ensifer] | | | |
|  |  | | chromosome 1 | | OV14_RS17950 | | WP_025427981.1 | | | MULTISPECIES: hypothetical protein [Ensifer] | | | |
|  |  | | chromosome 1 | | OV14_RS18065 | | WP_025428004.1 | | | MULTISPECIES: microcin B17 transporter [Ensifer] | | | |
|  |  | | chromosome 1 | | OV14_RS18720 | | WP_025428131.1 | | | MULTISPECIES: antibiotic biosynthesis monooxygenase [Ensifer] | | | |
|  |  | | chromosome 1 | | OV14_RS18750 | | WP_025428137.1 | | | MULTISPECIES: urease subunit beta [Ensifer] | | | |
|  |  | | chromosome 2 | | OV14_RS19360 | | WP_025428252.1 | | | MULTISPECIES: TetR family transcriptional regulator [Ensifer] | | | |
|  |  | | chromosome 2 | | OV14_RS20930 | | WP_025428551.1 | | | polysaccharide deacetylase [Ensifer adhaerens] | | | |
|  |  | | chromosome 2 | | OV14_RS21045 | | WP_025428574.1 | | | metal-binding protein [Ensifer adhaerens] | | | |
|  |  | | chromosome 2 | | OV14_RS21230 | | WP_025428611.1 | | | MULTISPECIES: transcriptional regulator [Ensifer] | | | |
|  |  | | chromosome 2 | | OV14_RS21575 | | WP_025428678.1 | | | MULTISPECIES: CoA transferase [Ensifer] | | | |
|  |  | | chromosome 2 | | OV14_RS21605 | | WP_025428684.1 | | | MULTISPECIES: glycosyl transferase [Ensifer] | | | |
|  |  | | chromosome 2 | | OV14_RS22345 | | WP_025428827.1 | | | MULTISPECIES: peptide ABC transporter [Ensifer] | | | |
|  |  | | chromosome 2 | | OV14_RS22535 | | WP_038578273.1 | | | MULTISPECIES: 1,3-1,4-beta-glycanase [Ensifer] | | | |
|  |  | | chromosome 2 | | OV14_RS22545 | | WP_025428867.1 | | | MULTISPECIES: succinoglycan biosynthesis protein exoa [Ensifer] | | | |
|  |  | | chromosome 2 | | OV14_RS22550 | | WP_025428868.1 | | | MULTISPECIES: glycosyl transferase family A [Ensifer] | | | |
|  |  | | chromosome 2 | | OV14_RS22740 | | WP_025428904.1 | | | MULTISPECIES: hypothetical protein [Ensifer] | | | |
|  |  | | chromosome 2 | | OV14_RS22745 | | WP_025428905.1 | | | MULTISPECIES: glycine oxidase ThiO [Ensifer] | | | |
|  |  | | chromosome 2 | | OV14_RS23330 | | WP_025429009.1 | | | TetR family transcriptional regulator [Ensifer adhaerens] | | | |
|  |  | | chromosome 2 | | OV14_RS24220 | | WP_038578389.1 | | | MULTISPECIES: acyl-CoA thioesterase [Ensifer] | | | |
|  |  | | chromosome 2 | | OV14_RS24455 | | WP_025429229.1 | | | MULTISPECIES: osmotically inducible protein C [Ensifer] | | | |
|  |  | | chromosome 2 | | OV14_RS25530 | | WP_025429433.1 | | | arylsulfatase [Ensifer adhaerens] | | | |
|  |  | | chromosome 2 | | OV14_RS25710 | | WP_025429468.1 | | | hypothetical protein [Ensifer adhaerens] | | | |
|  |  | | chromosome 2 | | OV14_RS25780 | | WP_025429479.1 | | | cytochrome C biogenesis protein DipZ [Ensifer adhaerens] | | | |
|  |  | | chromosome 2 | | OV14_RS25785 | | WP_025429480.1 | | | hydrolase [Ensifer adhaerens] | | | |
|  |  | | chromosome 2 | | OV14_RS26010 | | WP_025429524.1 | | | MULTISPECIES: cytochrome o ubiquinol oxidase subunit IV [Ensifer] | | | |
|  |  | | chromosome 2 | | OV14_RS26335 | | WP_025429589.1 | | | MULTISPECIES: GYD domain-containing protein [Ensifer] | | | |
|  |  | | chromosome 2 | | OV14_RS26595 | | WP_025429640.1 | | | MULTISPECIES: peptidase M20 [Ensifer] | | | |
|  |  | | plasmid b | | OV14_RS28890 | | WP_025430076.1 | | | MULTISPECIES: hypothetical protein [Ensifer] | | | |
|  |  | | plasmid b | | OV14_RS29570 | | WP_025430206.1 | | | coproporphyrinogen III oxidase [Ensifer adhaerens] | | | |
|  |  | | plasmid b | | OV14_RS29755 | | WP_025430241.1 | | | tRNA-specific adenosine deaminase [Ensifer adhaerens] | | | |
|  |  | | plasmid b | | OV14_RS30095 | | WP_025430309.1 | | | MULTISPECIES: hypothetical protein [Ensifer] | | | |
|  |  | | plasmid b | | OV14_RS31345 | | WP_025430552.1 | | | ABC transporter substrate-binding protein [Ensifer adhaerens] | | | |
|  |  | | plasmid b | | OV14_RS31435 | | WP_025430570.1 | | | tail-specific protease [Ensifer adhaerens] | | | |
|  |  | | plasmid b | | OV14_RS32130 | | Pseudogene | | | Pseudogene | | | |
|  |  | | plasmid b | | OV14_RS32455 | | WP_025430765.1 | | | MBL fold metallo-hydrolase [Ensifer adhaerens] | | | |
|  |  | | plasmid b | | OV14_RS32495 | | WP_025430773.1 | | | NUDIX hydrolase [Ensifer adhaerens] | | | |
|  |  | | plasmid b | | OV14_RS32745 | | WP_025430821.1 | | | D-tyrosyl-tRNA(Tyr) deacylase [Ensifer adhaerens] | | | |
|  |  | | plasmid b | | OV14_RS32760 | | WP_025430822.1 | | | hypothetical protein [Ensifer adhaerens] | | | |
|  |  | | plasmid b | | OV14_RS32825 | | WP_025430835.1 | | | hypothetical protein [Ensifer adhaerens] | | | |
|  |  | | plasmid b | | OV14_RS33190 | | WP_025430903.1 | | | short-chain dehydrogenase [Ensifer adhaerens] | | | |
|  |  | | plasmid b | | OV14_RS33360 | | WP_025430934.1 | | | ABC transporter substrate-binding protein [Ensifer adhaerens] | | | |
|  |  | | plasmid b | | OV14_RS33605 | | WP_025430983.1 | | | ABC transporter substrate-binding protein [Ensifer adhaerens] | | | |
|  |  | | plasmid b | | OV14_RS34550 | | WP_025431153.1 | | | hypothetical protein [Ensifer adhaerens] | | | |
|  |  | | plasmid b | | OV14_RS34780 | | WP_018325461.1 | | | MULTISPECIES: hypothetical protein [Rhizobiaceae] | | | |
|  |  | | plasmid b | | OV14_RS35545 | | WP_064742397.1 | | | hypothetical protein [Ensifer adhaerens] | | | |
|  |  | | plasmid b | | OV14_RS35590 | | WP_025431346.1 | | | LacI family transcriptional regulator [Ensifer adhaerens] | | | |
|  |  | | plasmid b | | OV14_RS36030 | | WP_025431429.1 | | | hypothetical protein [Ensifer adhaerens] | | | |
|  |  | | plasmid b | | OV14_RS36070 | | Pseudogene | | | Pseudogene | | | |
|  |  | | plasmid b | | OV14_RS36665 | | Pseudogene | | | Pseudogene | | | |
|  |  | | plasmid b | | OV14_RS36235 | | WP_025431466.1 | | | diaminopimelate decarboxylase | | | |
|  |  | | plasmid b | | OV14_RS36240 | | WP_018860235.1 | | | Pseudogene | | | |
|  |  | | plasmid 5105 | | virD1 | | [ABK01307.1](https://www.ncbi.nlm.nih.gov/protein/116585226) | | | Type IV secretion system T-DNA border endonuclease. Cleavage accessory protein | | | |
|  |  | | plasmid 5105 | | virB6 | | [ABK01298.1](https://www.ncbi.nlm.nih.gov/protein/116585217) | | | Type IV secretion system protein VirB6. T-DNA transfer | | | |
| D2(BR) D3(BR) | 2 | | chromosome 1 | OV14_RS17395 | | WP_025427873.1 | | | | WP_025427873.1 MULTISPECIES: hypothetical protein [Ensifer] | | | |
|  |  | | chromosome 2 | OV14_RS25020 | | WP_025429339.1 | | | | WP_025429339.1 hypothetical protein [Ensifer adhaerens] | | | |
| D2(BR) D5(BR) | 2 | | chromosome 1 | OV14_RS02525 | | WP_025425011.1 | | | | WP_025425011.1 MULTISPECIES: hypothetical protein [Ensifer] | | | |
|  |  | | chromosome 1 | OV14_RS14490 | | WP_025427308.1 | | | | WP_025427308.1 MULTISPECIES: molecular chaperone DnaJ [Ensifer] | | | |
| D2(BR) D7(BR) | 3 | | chromosome 1 | OV14_RS13910 | | WP_025427193.1 | | | | WP_025427193.1 MULTISPECIES: transcriptional regulator [Ensifer] | | | |
|  |  | | chromosome 1 | OV14_RS14965 | | WP_025427402.1 | | | | WP_025427402.1 MULTISPECIES: hypothetical protein [Ensifer] | | | |
|  |  | | chromosome 1 | OV14_RS14970 | | WP_025427403.1 | | | | WP_025427403.1 MULTISPECIES: hypothetical protein [Ensifer] | | | |
| D3(BR) D5(BR) | 19 | | chromosome 1 | OV14_RS03175 | | WP_025425136.1 | | | | WP_025425136.1 MULTISPECIES: hypothetical protein [Ensifer] | | | |
|  |  | | chromosome 1 | OV14_RS06860 | | WP_025425848.1 | | | | WP_025425848.1 MULTISPECIES: ubiquinol oxidase subunit II [Ensifer] | | | |
|  |  | | chromosome 1 | OV14_RS06865 | | WP_025425849.1 | | | | WP_025425849.1 MULTISPECIES: cytochrome ubiquinol oxidase subunit I [Ensifer] | | | |
|  |  | | chromosome 1 | OV14_RS09500 | | WP_025426360.1 | | | | WP_025426360.1 MULTISPECIES: membrane protein [Ensifer] | | | |
|  |  | | chromosome 1 | OV14_RS09510 | | WP_025426362.1 | | | | WP_025426362.1 MULTISPECIES: diacylglycerol kinase [Ensifer] | | | |
|  |  | | chromosome 1 | OV14_RS10065 | | WP_025426471.1 | | | | WP_025426471.1 MULTISPECIES: MarR family transcriptional regulator [Ensifer] | | | |
|  |  | | chromosome 1 | OV14_RS10765 | | WP_025426602.1 | | | | WP_025426602.1 MULTISPECIES: ABC transporter [Ensifer] | | | |
|  |  | | chromosome 1 | OV14_RS14555 | | WP_025427321.1 | | | | WP_025427321.1 MULTISPECIES: zinc ABC transporter substrate-binding protein [Ensifer] | | | |
|  |  | | chromosome 1 | OV14_RS14905 | | WP_025427390.1 | | | | WP_025427390.1 MULTISPECIES: hypothetical protein [Ensifer] | | | |
|  |  | | chromosome 1 | OV14_RS16060 | | WP_025427616.1 | | | | WP_025427616.1 MULTISPECIES: NADH dehydrogenase [Ensifer] | | | |
|  |  | | chromosome 1 | OV14_RS17525 | | WP_025427898.1 | | | | WP_025427898.1 MULTISPECIES: hypothetical protein [Ensifer] | | | |
|  |  | | chromosome 1 | OV14_RS18835 | | WP_025428154.1 | | | | WP_025428154.1 MULTISPECIES: response regulator [Ensifer] | | | |
|  |  | | chromosome 2 | OV14_RS19405 | | WP_025428259.1 | | | | WP_025428259.1 MULTISPECIES: hypothetical protein [Ensifer] | | | |
|  |  | | chromosome 2 | OV14_RS22870 | | WP_025428930.1 | | | | WP_025428930.1 hypothetical protein [Ensifer adhaerens] | | | |
|  |  | | chromosome 2 | OV14_RS22875 | | WP_025428931.1 | | | | WP_025428931.1 preprotein translocase subunit TatC [Ensifer adhaerens] | | | |
|  |  | | chromosome 2 | OV14_RS24520 | | WP_025429242.1 | | | | WP_025429242.1 hypothetical protein [Ensifer adhaerens] | | | |
|  |  | | chromosome 2 | OV14_RS26500 | | WP_025429621.1 | | | | WP_025429621.1 MULTISPECIES: hypothetical protein [Ensifer] | | | |
|  |  | | chromosome 2 | OV14_RS26795 | | WP_025429680.1 | | | | WP_025429680.1 MULTISPECIES: glycosyl transferase family 1 [Ensifer] | | | |
|  |  | | plasmid b | OV14_RS32635 | | WP_025430799.1 | | | | WP_025430799.1 NADP-dependent oxidoreductase [Ensifer adhaerens] | | | |
| D3(BR) D7(BR) | 6 | | chromosome 1 | OV14_RS03145 | | WP_025425131.1 | | | | WP_025425131.1 hypothetical protein [Ensifer adhaerens] | | | |
|  |  | | chromosome 1 | OV14_RS04150 | | WP_025425327.1 | | | | WP_025425327.1 MULTISPECIES: membrane protein [Ensifer] | | | |
|  |  | | chromosome 1 | OV14_RS11255 | | WP_025426689.1 | | | | WP_025426689.1 MULTISPECIES: mononuclear molybdenum enzyme YedY [Ensifer] | | | |
|  |  | | chromosome 1 | OV14_RS16315 | | WP_025427667.1 | | | | WP_025427667.1 MULTISPECIES: transcriptional regulator [Ensifer] | | | |
|  |  | | plasmid b | OV14_RS30945 | | WP_041693052.1 | | | | WP_041693052.1 periplasmic protein [Ensifer adhaerens] | | | |
|  |  | | plasmid b | OV14_RS31110 | | WP_025430504.1 | | | | WP_025430504.1 hypothetical protein [Ensifer adhaerens] | | | |
| D5(BR) D7(BR) | 137 | | chromosome 1 | OV14_RS00570 | | WP_025424643.1 | | | | MULTISPECIES: membrane protein [Ensifer] | | | |
|  |  | | chromosome 1 | OV14_RS00580 | | WP_038575656.1 | | | | MULTISPECIES: gamma-glutamylcyclotransferase [Ensifer] | | | |
|  |  | | chromosome 1 | OV14_RS00625 | | WP_025424653.1 | | | | MULTISPECIES: 50S ribosomal protein L28 [Ensifer] | | | |
|  |  | | chromosome 1 | OV14_RS00655 | | WP_025424659.1 | | | | MULTISPECIES: BolA family transcriptional regulator [Ensifer] | | | |
|  |  | | chromosome 1 | OV14_RS00685 | | WP_025424665.1 | | | | MULTISPECIES: acetyl-CoA carboxylase carboxyltransferase subunit alpha [Ensifer] | | | |
|  |  | | chromosome 1 | OV14_RS00735 | | WP_025424675.1 | | | | MULTISPECIES: hydroxyacid dehydrogenase [Ensifer] | | | |
|  |  | | chromosome 1 | OV14_RS01180 | | WP_025424760.1 | | | | phosphoglycerate kinase [Ensifer adhaerens] | | | |
|  |  | | chromosome 1 | OV14_RS01305 | | WP_025424784.1 | | | | MULTISPECIES: hypothetical protein [Ensifer] | | | |
|  |  | | chromosome 1 | OV14_RS01410 | | WP_025424805.1 | | | | MULTISPECIES: hypothetical protein [Ensifer] | | | |
|  |  | | chromosome 1 | OV14_RS01500 | | WP_025424823.1 | | | | MULTISPECIES: response regulator [Ensifer] | | | |
|  |  | | chromosome 1 | OV14_RS01715 | | WP_025424863.1 | | | | MULTISPECIES: hypothetical protein [Ensifer] | | | |
|  |  | | chromosome 1 | OV14_RS01885 | | WP_025424895.1 | | | | MULTISPECIES: hypothetical protein [Ensifer] | | | |
|  |  | | chromosome 1 | OV14_RS01975 | | WP_025424913.1 | | | | MULTISPECIES: TetR family transcriptional regulator [Ensifer] | | | |
|  |  | | chromosome 1 | OV14_RS02590 | | WP_025425024.1 | | | | MULTISPECIES: transcriptional regulator [Ensifer] | | | |
|  |  | | chromosome 1 | OV14_RS02830 | | WP_025425070.1 | | | | MULTISPECIES: fructose-6-phosphate aldolase [Ensifer] | | | |
|  |  | | chromosome 1 | OV14_RS03260 | | WP_025425152.1 | | | | MULTISPECIES: hypothetical protein [Ensifer] | | | |
|  |  | | chromosome 1 | OV14_RS03270 | | WP_025425154.1 | | | | 2-octaprenyl-6-methoxyphenyl hydroxylase [Ensifer adhaerens] | | | |
|  |  | | chromosome 1 | OV14_RS03375 | | WP_025425174.1 | | | | MULTISPECIES: 30S ribosomal protein S16 [Ensifer] | | | |
|  |  | | chromosome 1 | OV14_RS03380 | | WP_025425175.1 | | | | MULTISPECIES: chorismate mutase [Ensifer] | | | |
|  |  | | chromosome 1 | OV14_RS03455 | | WP_038576579.1 | | | | MULTISPECIES: hypothetical protein [Ensifer] | | | |
|  |  | | chromosome 1 | OV14_RS03750 | | WP_025425248.1 | | | | aspartate aminotransferase [Ensifer adhaerens] | | | |
|  |  | | chromosome 1 | OV14_RS03790 | | WP_025425256.1 | | | | MULTISPECIES: sugar ABC transporter ATPase [Ensifer] | | | |
|  |  | | chromosome 1 | OV14_RS03805 | | WP_025425259.1 | | | | MULTISPECIES: sugar ABC transporter substrate-binding protein [Ensifer] | | | |
|  |  | | chromosome 1 | OV14_RS03810 | | WP_025425260.1 | | | | MULTISPECIES: LacI family transcriptional regulator [Ensifer] | | | |
|  |  | | chromosome 1 | OV14_RS04220 | | WP_025425341.1 | | | | MULTISPECIES: adenosylhomocysteinase [Ensifer] | | | |
|  |  | | chromosome 1 | OV14_RS04375 | | WP_025425371.1 | | | | ATP-dependent helicase HrpB [Ensifer adhaerens] | | | |
|  |  | | chromosome 1 | OV14_RS04385 | | WP_051509141.1 | | | | MULTISPECIES: ABC transporter permease [Ensifer] | | | |
|  |  | | chromosome 1 | OV14_RS05455 | | WP_025425575.1 | | | | MULTISPECIES: aminopeptidase [Ensifer] | | | |
|  |  | | chromosome 1 | OV14_RS05490 | | WP_025425581.1 | | | | MULTISPECIES: aromatic amino acid aminotransferase [Ensifer] | | | |
|  |  | | chromosome 1 | OV14_RS05510 | | WP_009492215.1 | | | |  | | | |
|  |  | | chromosome 1 | OV14_RS05595 | | WP_025425601.1 | | | | MULTISPECIES: peptidase [Ensifer] | | | |
|  |  | | chromosome 1 | OV14_RS05825 | | WP_025425646.1 | | | | MULTISPECIES: class II glutamine amidotransferase [Ensifer] | | | |
|  |  | | chromosome 1 | OV14_RS05905 | | WP_025425661.1 | | | | MULTISPECIES: 30S ribosomal protein S20 [Ensifer] | | | |
|  |  | | chromosome 1 | OV14_RS36725 | | WP_025425763.1 | | | | MULTISPECIES: hypothetical protein [Ensifer] | | | |
|  |  | | chromosome 1 | OV14_RS06445 | | WP_025425767.1 | | | | MULTISPECIES: membrane protein [Ensifer] | | | |
|  |  | | chromosome 1 | OV14_RS06460 | | WP_025425770.1 | | | | MULTISPECIES: sugar ABC transporter [Ensifer] | | | |
|  |  | | chromosome 1 | OV14_RS06485 | | WP_025425775.1 | | | | MULTISPECIES: orotate phosphoribosyltransferase [Ensifer] | | | |
|  |  | | chromosome 1 | OV14_RS06625 | | WP_025425801.1 | | | | MULTISPECIES: GcrA cell cycle regulator [Ensifer] | | | |
|  |  | | chromosome 1 | OV14_RS06780 | | WP_025425832.1 | | | | MULTISPECIES: alkylhydroperoxidase [Ensifer] | | | |
|  |  | | chromosome 1 | OV14_RS06850 | | WP_025425846.1 | | | | MULTISPECIES: chorismate mutase [Ensifer] | | | |
|  |  | | chromosome 1 | OV14_RS06990 | | WP_025425873.1 | | | | MULTISPECIES: UDP-N-acetylglucosamine 1-carboxyvinyltransferase [Ensifer] | | | |
|  |  | | chromosome 1 | OV14_RS07680 | | WP_025426006.1 | | | | MULTISPECIES: hypothetical protein [Ensifer] | | | |
|  |  | | chromosome 1 | OV14_RS07865 | | WP_025426042.1 | | | | MULTISPECIES: glycolate oxidase [Ensifer] | | | |
|  |  | | chromosome 1 | OV14_RS08045 | | WP_025426076.1 | | | | MULTISPECIES: hypothetical protein [Ensifer] | | | |
|  |  | | chromosome 1 | OV14_RS08295 | | WP_025426126.1 | | | | MFS transporter [Ensifer adhaerens] | | | |
|  |  | | chromosome 1 | OV14_RS08335 | | WP_025426134.1 | | | | MULTISPECIES: hypothetical protein [Ensifer] | | | |
|  |  | | chromosome 1 | OV14_RS08400 | | WP_038575941.1 | | | | MULTISPECIES: 1-deoxy-D-xylulose-5-phosphate synthase [Ensifer] | | | |
|  |  | | chromosome 1 | OV14_RS08475 | | WP_025426162.1 | | | | MULTISPECIES: pyridoxamine 5'-phosphate oxidase [Ensifer] | | | |
|  |  | | chromosome 1 | OV14_RS08630 | | WP_025426192.1 | | | | MULTISPECIES: branched chain amino acid ABC transporter substrate-binding protein [Ensifer] | | | |
|  |  | | chromosome 1 | OV14_RS08725 | | WP_025426210.1 | | | | MULTISPECIES: hypothetical protein [Ensifer] | | | |
|  |  | | chromosome 1 | OV14_RS08910 | | WP_025426245.1 | | | | MULTISPECIES: glutathione S-transferase [Ensifer] | | | |
|  |  | | chromosome 1 | OV14_RS09075 | | WP_025426277.1 | | | | MULTISPECIES: superoxide dismutase [Ensifer] | | | |
|  |  | | chromosome 1 | OV14_RS09195 | | WP_025426301.1 | | | | MULTISPECIES: glutathione S-transferase [Ensifer] | | | |
|  |  | | chromosome 1 | OV14_RS09690 | | WP_025426397.1 | | | | MULTISPECIES: ATPase [Ensifer] | | | |
|  |  | | chromosome 1 | OV14_RS09695 | | WP_025426398.1 | | | | MULTISPECIES: hypothetical protein [Ensifer] | | | |
|  |  | | chromosome 1 | OV14_RS09880 | | WP_025426435.1 | | | | MULTISPECIES: amidophosphoribosyltransferase [Ensifer] | | | |
|  |  | | chromosome 1 | OV14_RS09905 | | WP_051509157.1 | | | | MULTISPECIES: branched-chain amino acid ABC transporter permease [Ensifer] | | | |
|  |  | | chromosome 1 | OV14_RS10275 | | WP_025426510.1 | | | | hypothetical protein [Ensifer adhaerens] | | | |
|  |  | | chromosome 1 | OV14_RS10310 | | WP_025426517.1 | | | | MULTISPECIES: hypothetical protein [Ensifer] | | | |
|  |  | | chromosome 1 | OV14_RS10485 | | WP_025426550.1 | | | | MULTISPECIES: heme A synthase [Ensifer] | | | |
|  |  | | chromosome 1 | OV14_RS10665 | | WP_025426582.1 | | | | MULTISPECIES: NADH-quinone oxidoreductase subunit C [Ensifer] | | | |
|  |  | | chromosome 1 | OV14_RS10935 | | WP_025426634.1 | | | | MULTISPECIES: glycosyl hydrolase family 5 [Ensifer] | | | |
|  |  | | chromosome 1 | OV14_RS11090 | | WP_025426661.1 | | | | MULTISPECIES: hypothetical protein [Ensifer] | | | |
|  |  | | chromosome 1 | OV14_RS11160 | | WP_025426672.1 | | | | MULTISPECIES: 50S ribosomal protein L29 [Ensifer] | | | |
|  |  | | chromosome 1 | OV14_RS11740 | | WP_003535434.1 | | | | MULTISPECIES: RNA-binding protein Hfq [Sinorhizobium/Ensifer group] | | | |
|  |  | | chromosome 1 | OV14_RS11915 | | WP_025426817.1 | | | | MULTISPECIES: ATP-binding protein [Ensifer] | | | |
|  |  | | chromosome 1 | OV14_RS12500 | | WP_025426929.1 | | | | hypothetical protein [Ensifer adhaerens] | | | |
|  |  | | chromosome 1 | OV14_RS13490 | | WP_025427109.1 | | | | MULTISPECIES: hypothetical protein [Ensifer] | | | |
|  |  | | chromosome 1 | OV14_RS13510 | | WP_025427113.1 | | | | MULTISPECIES: glycine dehydrogenase (aminomethyl-transferring) [Ensifer] | | | |
|  |  | | chromosome 1 | OV14_RS13770 | | WP_025427165.1 | | | | MULTISPECIES: glutaminase [Ensifer] | | | |
|  |  | | chromosome 1 | OV14_RS14740 | | WP_025427357.1 | | | | MULTISPECIES: beta-ketoacyl-ACP synthase II [Ensifer] | | | |
|  |  | | chromosome 1 | OV14_RS15580 | | WP_025427522.1 | | | | MULTISPECIES: cell division protein FtsZ [Ensifer] | | | |
|  |  | | chromosome 1 | OV14_RS15740 | | WP_025427553.1 | | | | MULTISPECIES: oxidoreductase [Ensifer] | | | |
|  |  | | chromosome 1 | OV14_RS15910 | | WP_025427586.1 | | | | MULTISPECIES: transcriptional regulator [Ensifer] | | | |
|  |  | | chromosome 1 | OV14_RS16320 | | WP_025427668.1 | | | | serine protease [Ensifer adhaerens] | | | |
|  |  | | chromosome 1 | OV14_RS16360 | | WP_025427676.1 | | | | MULTISPECIES: hypothetical protein [Ensifer] | | | |
|  |  | | chromosome 1 | OV14_RS16445 | | WP_025427692.1 | | | | MULTISPECIES: isochorismatase [Ensifer] | | | |
|  |  | | chromosome 1 | OV14_RS16520 | | WP_025427706.1 | | | | MULTISPECIES: membrane protein [Ensifer] | | | |
|  |  | | chromosome 1 | OV14_RS16525 | | WP_025427707.1 | | | | MULTISPECIES: branched-chain amino acid ABC transporter permease [Ensifer] | | | |
|  |  | | chromosome 1 | OV14_RS16875 | | WP_025427775.1 | | | | MULTISPECIES: riboflavin synthase subunit beta [Ensifer] | | | |
|  |  | | chromosome 1 | OV14_RS17535 | | WP_025427900.1 | | | | MULTISPECIES: marine proteobacterial sortase target protein [Ensifer] | | | |
|  |  | | chromosome 1 | OV14_RS17585 | | WP_025427909.1 | | | | MULTISPECIES: hypothetical protein [Ensifer] | | | |
|  |  | | chromosome 1 | OV14_RS17640 | | WP_025427920.1 | | | | MULTISPECIES: prolipoprotein diacylglyceryl transferase [Ensifer] | | | |
|  |  | | chromosome 1 | OV14_RS17885 | | WP_025427968.1 | | | | MULTISPECIES: ATP-dependent Clp protease proteolytic subunit [Ensifer] | | | |
|  |  | | chromosome 1 | OV14_RS18575 | | WP_025428102.1 | | | | MULTISPECIES: sorbitol dehydrogenase [Ensifer] | | | |
|  |  | | chromosome 1 | OV14_RS18580 | | WP_025428103.1 | | | | MULTISPECIES: glycerol-3-phosphate ABC transporter ATPase [Ensifer] | | | |
|  |  | | chromosome 1 | OV14_RS18585 | | WP_025428104.1 | | | | MULTISPECIES: mannitol ABC transporter permease [Ensifer] | | | |
|  |  | | chromosome 1 | OV14_RS18705 | | WP_025428128.1 | | | | MULTISPECIES: urease accessory protein UreG [Ensifer] | | | |
|  |  | | chromosome 2 | OV14_RS19235 | | WP_025428228.1 | | | | SDR family oxidoreductase [Ensifer adhaerens] | | | |
|  |  | | chromosome 2 | OV14_RS19240 | | WP_038578040.1 | | | | multidrug MFS transporter [Ensifer adhaerens] | | | |
|  |  | | chromosome 2 | OV14_RS19795 | | WP_025428336.1 | | | | MULTISPECIES: TetR family transcriptional regulator [Ensifer] | | | |
|  |  | | chromosome 2 | OV14_RS19800 | | WP_051509220.1 | | | | hemolysin secretion protein D [Ensifer adhaerens] | | | |
|  |  | | chromosome 2 | OV14_RS19805 | | WP_025428338.1 | | | | multidrug transporter AcrB [Ensifer adhaerens] | | | |
|  |  | | chromosome 2 | OV14_RS20365 | | WP_025428443.1 | | | | LacI family transcriptional regulator [Ensifer adhaerens] | | | |
|  |  | | chromosome 2 | OV14_RS20370 | | WP_063963325.1 | | | | MULTISPECIES: LacI family transcriptional regulator [Ensifer] | | | |
|  |  | | chromosome 2 | OV14_RS20390 | | WP_025428448.1 | | | | MULTISPECIES: tagatose 3-epimerase [Ensifer] | | | |
|  |  | | chromosome 2 | OV14_RS20395 | | WP_038578133.1 | | | | carbohydrate kinase [Ensifer adhaerens] | | | |
|  |  | | chromosome 2 | OV14_RS20400 | | WP_025428450.1 | | | | fuculose phosphate aldolase [Ensifer adhaerens] | | | |
|  |  | | chromosome 2 | OV14_RS20505 | | WP_025428469.1 | | | | MULTISPECIES: sulfate transporter subunit [Ensifer] | | | |
|  |  | | chromosome 2 | OV14_RS21120 | | WP_025428589.1 | | | | MULTISPECIES: ureidoglycolate hydrolase [Ensifer] | | | |
|  |  | | chromosome 2 | OV14_RS21420 | | WP_025428647.1 | | | | MULTISPECIES: ABC transporter substrate-binding protein [Ensifer] | | | |
|  |  | | chromosome 2 | OV14_RS21600 | | WP_025428683.1 | | | | MULTISPECIES: acyltransferase [Ensifer] | | | |
|  |  | | chromosome 2 | OV14_RS21610 | | WP_051509288.1 | | | | MULTISPECIES: glycosyl transferase [Ensifer] | | | |
|  |  | | chromosome 2 | OV14_RS21615 | | WP_025428686.1 | | | | MULTISPECIES: ABC transporter ATP-binding protein [Ensifer] | | | |
|  |  | | chromosome 2 | OV14_RS21665 | | WP_025428696.1 | | | | MULTISPECIES: esterase [Ensifer] | | | |
|  |  | | chromosome 2 | OV14_RS21930 | | WP_025428746.1 | | | | MULTISPECIES: 2-hydroxyhepta-2,4-diene-1,7-dioate isomerase [Ensifer] | | | |
|  |  | | chromosome 2 | OV14_RS22500 | | WP_025428858.1 | | | | MULTISPECIES: exopolysaccharide biosynthesis protein [Ensifer] | | | |
|  |  | | chromosome 2 | OV14_RS22515 | | WP_025428861.1 | | | | MULTISPECIES: succinoglycan biosynthesis protein exov [Ensifer] | | | |
|  |  | | chromosome 2 | OV14_RS22520 | | WP_025428862.1 | | | | MULTISPECIES: succinoglycan biosynthesis protein exow [Ensifer] | | | |
|  |  | | chromosome 2 | OV14_RS22525 | | WP_025428863.1 | | | | lipopolysaccharide biosynthesis protein [Ensifer adhaerens] | | | |
|  |  | | chromosome 2 | OV14_RS22560 | | WP_025428870.1 | | | | MULTISPECIES: UTP--glucose-1-phosphate uridylyltransferase [Ensifer] | | | |
|  |  | | chromosome 2 | OV14_RS22565 | | WP_025428871.1 | | | | MULTISPECIES: chain-length determining protein [Ensifer] | | | |
|  |  | | chromosome 2 | OV14_RS23375 | | WP_025429017.1 | | | | hypothetical protein [Ensifer adhaerens] | | | |
|  |  | | chromosome 2 | OV14_RS24925 | | WP_025429320.1 | | | | Bcr/CflA family drug resistance efflux transporter [Ensifer adhaerens] | | | |
|  |  | | chromosome 2 | OV14_RS25060 | | WP_025429347.1 | | | | membrane protein [Ensifer adhaerens] | | | |
|  |  | | chromosome 2 | OV14_RS25760 | | WP_025429475.1 | | | | peroxiredoxin [Ensifer adhaerens] | | | |
|  |  | | chromosome 2 | OV14_RS26395 | | WP_025429601.1 | | | | oxidoreductase [Ensifer adhaerens] | | | |
|  |  | | chromosome 2 | OV14_RS27405 | | WP_025429795.1 | | | | hypothetical protein [Ensifer adhaerens] | | | |
|  |  | | chromosome 2 | OV14_RS27425 | | WP_025429799.1 | | | | ABC transporter substrate-binding protein [Ensifer adhaerens] | | | |
|  |  | | chromosome 2 | OV14_RS27430 | | WP_025429800.1 | | | | MULTISPECIES: ABC transporter permease [Ensifer] | | | |
|  |  | | chromosome 2 | OV14_RS27435 | | WP_025429801.1 | | | | MULTISPECIES: ABC transporter permease [Ensifer] | | | |
|  |  | | chromosome 2 | OV14_RS27445 | | WP_025429803.1 | | | | MULTISPECIES: ABC transporter ATP-binding protein [Ensifer] | | | |
|  |  | | chromosome 2 | OV14_RS27450 | | WP_038578014.1 | | | | alpha-mannosidase [Ensifer adhaerens] | | | |
|  |  | | chromosome 2 | OV14_RS27455 | | WP_011428866.1 | | | |  | | | |
|  |  | | chromosome 2 | OV14_RS27595 | | WP_025429833.1 | | | | MULTISPECIES: aldo/keto reductase [Ensifer] | | | |
|  |  | | chromosome 2 | OV14_RS27765 | | WP_025429867.1 | | | | MULTISPECIES: ABC transporter ATP-binding protein [Ensifer] | | | |
|  |  | | plasmid b | OV14_RS29520 | | WP_025430196.1 | | | | MULTISPECIES: Crp/Fnr family transcriptional regulator [Ensifer] | | | |
|  |  | | plasmid b | OV14_RS36585 | | WP_025430310.1 | | | | MULTISPECIES: hypothetical protein [Ensifer] | | | |
|  |  | | plasmid b | OV14_RS31085 | | WP_025430500.1 | | | | dioxygenase [Ensifer adhaerens] | | | |
|  |  | | plasmid b | OV14_RS33540 | | WP_025430970.1 | | | | hypothetical protein [Ensifer adhaerens] | | | |
|  |  | | plasmid b | OV14_RS34330 | | WP_051509352.1 | | | | hypothetical protein [Ensifer adhaerens] | | | |
|  |  | | plasmid b | OV14_RS35725 | | WP_025431370.1 | | | | MULTISPECIES: DNA-binding protein [Ensifer] | | | |
|  |  | | plasmid b | OV14_RS36080 | | WP_025431437.1 | | | | hypothetical protein [Ensifer adhaerens] | | | |
|  |  | | plasmid c | OV14_RS28405 | | WP_038578862.1 | | | | DUF159 family protein [Ensifer adhaerens] | | | |
|  |  | | plasmid c | OV14_RS28505 | | WP_012648969.1 | | | | MULTISPECIES: DNA-binding protein [Rhizobiaceae] | | | |
|  |  | | plasmid c | OV14_RS28545 | | WP_025430015.1 | | | | conjugal transfer protein TraB [Ensifer adhaerens] | | | |
|  |  | | plasmid c | OV14_RS28805 | | WP_025430065.1 | | | | conjugal transfer protein TrbD [Ensifer adhaerens] | | | |
| D3(BR)  D5(BR)  D7(BR) | 65 | | chromosome 1 | OV14_RS00890 | | WP_025424704.1 | | | >WP_025424704.1 MULTISPECIES: acetyltransferase [Ensifer] | | | |  |
|  |  | | chromosome 1 | OV14_RS02785 | | WP_025425061.1 | | | >WP_025425061.1 MULTISPECIES: saccharopine dehydrogenase [Ensifer] | | | |  |
|  |  | | chromosome 1 | OV14_RS02965 | | WP_025425097.1 | | | >WP_025425097.1 MULTISPECIES: hypothetical protein [Ensifer] | | | |  |
|  |  | | chromosome 1 | OV14_RS03250 | | WP_025425150.1 | | | >WP_025425150.1 MULTISPECIES: co-chaperone YbbN [Ensifer] | | | |  |
|  |  | | chromosome 1 | OV14_RS03635 | | WP_025425225.1 | | | >WP_025425225.1 MULTISPECIES: 50S ribosomal protein L32 [Rhizobiaceae] | | | |  |
|  |  | | chromosome 1 | OV14_RS04365 | | WP_025425369.1 | | | >WP_025425369.1 MULTISPECIES: acid tolerance protein ActR [Ensifer] | | | |  |
|  |  | | chromosome 1 | OV14_RS04950 | | WP_025425478.1 | | | >WP_025425478.1 MULTISPECIES: HIT family protein [Ensifer] | | | |  |
|  |  | | chromosome 1 | OV14_RS06240 | | WP_025425728.1 | | | >WP_025425728.1 MULTISPECIES: esterase [Ensifer] | | | |  |
|  |  | | chromosome 1 | OV14_RS06280 | | WP_025425736.1 | | | >WP_025425736.1 MULTISPECIES: hypothetical protein [Ensifer] | | | |  |
|  |  | | chromosome 1 | OV14_RS06995 | | WP_025425874.1 | | | >WP_025425874.1 MULTISPECIES: hypothetical protein [Ensifer] | | | |  |
|  |  | | chromosome 1 | OV14_RS08195 | | WP_025426106.1 | | | >WP_025426106.1 MULTISPECIES: molybdenum cofactor biosynthesis protein [Ensifer] | | | |  |
|  |  | | chromosome 1 | OV14_RS08500 | | WP_025426167.1 | | | >WP_025426167.1 MULTISPECIES: hypothetical protein [Ensifer] | | | |  |
|  |  | | chromosome 1 | OV14_RS08520 | | WP_025426170.1 | | | >WP_025426170.1 MULTISPECIES: invasion protein B [Ensifer] | | | |  |
|  |  | | chromosome 1 | OV14_RS08770 | | WP_025426218.1 | | | >WP_025426218.1 MULTISPECIES: hypothetical protein [Ensifer] | | | |  |
|  |  | | chromosome 1 | OV14_RS08985 | | WP_025426260.1 | | | >WP_025426260.1 MULTISPECIES: hypothetical protein [Ensifer] | | | |  |
|  |  | | chromosome 1 | OV14_RS09070 | | WP_025426276.1 | | | >WP_025426276.1 MULTISPECIES: hypothetical protein [Ensifer] | | | |  |
|  |  | | chromosome 1 | OV14_RS09185 | | WP_025426299.1 | | | >WP_025426299.1 MULTISPECIES: tautomerase [Ensifer] | | | |  |
|  |  | | chromosome 1 | OV14_RS09225 | | WP_025426307.1 | | | >WP_025426307.1 MULTISPECIES: glyoxalase [Ensifer] | | | |  |
|  |  | | chromosome 1 | OV14_RS10240 | | WP_025426503.1 | | | >WP_025426503.1 MULTISPECIES: copper oxidase [Ensifer] | | | |  |
|  |  | | chromosome 1 | OV14_RS10305 | | WP_025426516.1 | | | >WP_025426516.1 MULTISPECIES: delta-aminolevulinic acid dehydratase [Ensifer] | | | |  |
|  |  | | chromosome 1 | OV14_RS11010 | | WP_025426648.1 | | | >WP_025426648.1 MULTISPECIES: FMN-dependent NADH-azoreductase [Ensifer] | | | |  |
|  |  | | chromosome 1 | OV14_RS14425 | | WP_025427295.1 | | | >WP_025427295.1 MULTISPECIES: hypothetical protein [Ensifer] | | | |  |
|  |  | | chromosome 1 | OV14_RS14720 | | WP_025427353.1 | | | >WP_025427353.1 MULTISPECIES: hypothetical protein [Ensifer] | | | |  |
|  |  | | chromosome 1 | OV14_RS14915 | | WP_025427392.1 | | | >WP_025427392.1 MULTISPECIES: hypothetical protein [Ensifer] | | | |  |
|  |  | | chromosome 1 | OV14_RS15845 | | WP_025427574.1 | | | >WP_025427574.1 MULTISPECIES: hypothetical protein [Ensifer] | | | |  |
|  |  | | chromosome 1 | OV14_RS16465 | | WP_025427696.1 | | | >WP_025427696.1 MULTISPECIES: hypothetical protein [Ensifer] | | | |  |
|  |  | | chromosome 1 | OV14_RS16495 | | WP_025427701.1 | | | >WP_025427701.1 MULTISPECIES: copper chaperone [Ensifer] | | | |  |
|  |  | | chromosome 1 | OV14_RS17825 | | WP_038576325.1 | | | >WP_038576325.1 MULTISPECIES: glutathione-dependent reductase [Ensifer] | | | |  |
|  |  | | chromosome 1 | OV14_RS17935 | | WP_025427978.1 | | | >WP_025427978.1 MULTISPECIES: hypothetical protein [Ensifer] | | | |  |
|  |  | | chromosome 1 | OV14_RS17970 | | WP_025427985.1 | | | >WP_025427985.1 MULTISPECIES: cytochrome c family protein [Ensifer] | | | |  |
|  |  | | chromosome 1 | OV14_RS18470 | | WP_018095707.1 | | |  | | Pseudogene | |  |
|  |  | | chromosome 1 | OV14_RS18560 | | WP_025428099.1 | | | >WP_025428099.1 ribulokinase [Ensifer adhaerens] | | | |  |
|  |  | | chromosome 1 | OV14_RS18565 | | WP_025428100.1 | | | >WP_025428100.1 MULTISPECIES: hydrolase [Ensifer] | | | |  |
|  |  | | chromosome 1 | OV14_RS18570 | | WP_025428101.1 | | | >WP_025428101.1 MULTISPECIES: mannitol dehydrogenase [Ensifer] | | | |  |
|  |  | | chromosome 1 | OV14_RS18590 | | WP_025428105.1 | | | >WP_025428105.1 MULTISPECIES: sugar ABC transporter permease [Ensifer] | | | |  |
|  |  | | chromosome 1 | OV14_RS18595 | | WP_025428106.1 | | | >WP_025428106.1 MULTISPECIES: sugar ABC transporter substrate-binding protein [Ensifer] | | | |  |
|  |  | | chromosome 1 | OV14_RS18865 | | WP_025428160.1 | | | >WP_025428160.1 ring-cleaving dioxygenase [Ensifer adhaerens] | | | |  |
|  |  | | chromosome 2 | OV14_RS36830 | | WP_067698903.1 | | | >WP_067698903.1 DUF1127 domain-containing protein [Ensifer adhaerens] | | | |  |
|  |  | | chromosome 2 | OV14_RS19460 | | WP_025428270.1 | | | >WP_025428270.1 membrane protein [Ensifer adhaerens] | | | |  |
|  |  | | chromosome 2 | OV14_RS19735 | | WP_025428324.1 | | | >WP_025428324.1 MULTISPECIES: FAD-dependent oxidoreductase [Ensifer] | | | |  |
|  |  | | chromosome 2 | OV14_RS20730 | | WP_025428511.1 | | | >WP_025428511.1 MULTISPECIES: hypothetical protein [Ensifer] | | | |  |
|  |  | | chromosome 2 | OV14_RS21080 | | WP_025428581.1 | | | >WP_025428581.1 MULTISPECIES: Cu(I)-responsive transcriptional regulator [Ensifer] | | | |  |
|  |  | | chromosome 2 | OV14_RS22510 | | WP_025428860.1 | | | >WP_025428860.1 glycosyl transferase family A [Ensifer adhaerens] | | | |  |
|  |  | | chromosome 2 | OV14_RS22840 | | WP_025428924.1 | | | >WP_025428924.1 hypothetical protein [Ensifer adhaerens] | | | |  |
|  |  | | chromosome 2 | OV14_RS25000 | | WP_025429335.1 | | | >WP_025429335.1 poly(3-hydroxybutyrate) depolymerase [Ensifer adhaerens] | | | |  |
|  |  | | chromosome 2 | OV14_RS36835 | | WP_067698903.1 | | |  | | Pseudogene | |  |
|  |  | | chromosome 2 | OV14_RS26005 | | WP_051509305.1 | | | >WP_051509305.1 hypothetical protein [Ensifer adhaerens] | | | |  |
|  |  | | chromosome 2 | OV14_RS26015 | | WP_025429525.1 | | | >WP_025429525.1 MULTISPECIES: cytochrome o ubiquinol oxidase subunit III [Ensifer] | | | |  |
|  |  | | chromosome 2 | OV14_RS26020 | | WP_025429526.1 | | | >WP_025429526.1 MULTISPECIES: cytochrome ubiquinol oxidase subunit I [Ensifer] | | | |  |
|  |  | | chromosome 2 | OV14_RS26025 | | WP_025429527.1 | | | >WP_025429527.1 ubiquinol oxidase subunit II [Ensifer adhaerens] | | | |  |
|  |  | | chromosome 2 | OV14_RS27410 | | WP_025429796.1 | | | >WP_025429796.1 DUF5054 domain-containing protein [Ensifer adhaerens] | | | |  |
|  |  | | chromosome 2 | OV14_RS27415 | | WP_025429797.1 | | | >WP_025429797.1 mannose-6-phosphate isomerase [Ensifer adhaerens] | | | |  |
|  |  | | chromosome 2 | OV14_RS27420 | | WP_025429798.1 | | | >WP_025429798.1 MULTISPECIES: LacI family transcriptional regulator [Ensifer] | | | |  |
|  |  | | chromosome 2 | OV14_RS27605 | | WP_025429835.1 | | | >WP_025429835.1 MULTISPECIES: mannonate dehydratase [Ensifer] | | | |  |
|  |  | | chromosome 2 | OV14_RS27770 | | WP_025429868.1 | | | >WP_025429868.1 MULTISPECIES: ABC transporter permease [Ensifer] | | | |  |
|  |  | | chromosome 2 | OV14_RS27910 | | WP_025429895.1 | | | >WP_025429895.1 hypothetical protein [Ensifer adhaerens] | | | |  |
|  |  | | chromosome 2 | OV14_RS27915 | | WP_025429896.1 | | | >WP_025429896.1 MULTISPECIES: hypothetical protein [Ensifer] | | | |  |
|  |  | | plasmid b | OV14_RS30105 | | WP_025430311.1 | | | >WP_025430311.1 MULTISPECIES: FMN reductase [Ensifer] | | | |  |
|  |  | | plasmid b | OV14_RS32325 | | WP_025430740.1 | | | >WP_025430740.1 hypothetical protein [Ensifer adhaerens] | | | |  |
|  |  | | plasmid b | OV14_RS32450 | | WP_025430764.1 | | | >WP_025430764.1 hypothetical protein [Ensifer adhaerens] | | | |  |
|  |  | | plasmid b | OV14_RS32605 | | WP_025430794.1 | | | >WP_025430794.1 hypothetical protein [Ensifer adhaerens] | | | |  |
|  |  | | plasmid b | OV14_RS33040 | | WP_025430876.1 | | | >WP_025430876.1 cold-shock protein [Ensifer adhaerens] | | | |  |
|  |  | | plasmid b | OV14_RS35270 | | WP_025431283.1 | | | >WP_025431283.1 cold-shock protein [Ensifer adhaerens] | | | |  |
|  |  | | plasmid b | OV14_RS35595 | | WP_025431347.1 | | | >WP_025431347.1 sugar ABC transporter substrate-binding protein [Ensifer adhaerens] | | | |  |
|  |  | | plasmid b | OV14_RS35660 | | WP_025431359.1 | | | >WP_025431359.1 phosphatase [Ensifer adhaerens] | | | |  |
| D2(BR) D5(BR) D7(BR) | 1 | | chromosome 2 | OV14_RS25900 | | WP_025429502.1 | | | >WP_025429502.1 MULTISPECIES: hypothetical protein [Ensifer] | | | |  |
| D2(BR) D3(BR) D7(BR) | 1 | | chromosome 2 | OV14_RS24585 | | WP_025429255.1 | | | >WP_025429255.1 C4-dicarboxylate transporter [Ensifer adhaerens] | | | |  |
| D2(BR) D3(BR) D5(BR) | 3 | | chromosome 1 | OV14_RS02855 | | WP_025425075.1 | | | >WP_025425075.1 MULTISPECIES: hypothetical protein [Ensifer] | | | |  |
|  |  | | chromosome 2 | OV14_RS22435 | | WP_025428845.1 | | | >WP_025428845.1 MULTISPECIES: GntR family transcriptional regulator [Ensifer] | | | |  |
|  |  | | chromosome 2 | OV14_RS25015 | | WP_025429338.1 | | | >WP_025429338.1 CoA transferase [Ensifer adhaerens] | | | |  |
| D1(BR) D5(BR) D7(BR) | 1 | | plasmid b | OV14_RS31985 | | WP_025430676.1 | | | >WP_025430676.1 NmrA family transcriptional regulator [Ensifer adhaerens] | | | |  |
| D1(BR) D2(BR) D5(BR) | 1 | | plasmid b | OV14_RS31990 | | WP_025430677.1 | | | >WP_025430677.1 MBL fold metallo-hydrolase [Ensifer adhaerens] | | | |  |
| D2(BR) D3(BR) D5(BR) D7(BR) | 28 | | chromosome 1 | OV14_RS02530 | | WP_025425012.1 | | | >WP_025425012.1 MULTISPECIES: glutathione ABC transporter ATP-binding protein [Ensifer] | | | |  |
|  |  | | chromosome 1 | OV14_RS02535 | | WP_025425013.1 | | | >WP_025425013.1 MULTISPECIES: glyoxalase [Ensifer] | | | |  |
|  |  | | chromosome 1 | OV14_RS04360 | | WP_025425368.1 | | | >WP_025425368.1 MULTISPECIES: hypothetical protein [Ensifer] | | | |  |
|  |  | | chromosome 1 | OV14_RS04935 | | WP_025425475.1 | | | >WP_025425475.1 MULTISPECIES: glyoxalase [Ensifer] | | | |  |
|  |  | | chromosome 1 | OV14_RS06230 | | WP_025425726.1 | | | >WP_025425726.1 MULTISPECIES: multidrug efflux RND transporter permease subunit [Ensifer] | | | |  |
|  |  | | chromosome 1 | OV14_RS06235 | | WP_025425727.1 | | | >WP_025425727.1 MULTISPECIES: MexE family multidrug efflux RND transporter periplasmic adaptor subunit [Ensifer] | | | |  |
|  |  | | chromosome 1 | OV14_RS09345 | | WP_025426331.1 | | | >WP_025426331.1 MULTISPECIES: porin family protein [Ensifer] | | | |  |
|  |  | | chromosome 1 | OV14_RS10055 | | WP_025426469.1 | | | >WP_025426469.1 MULTISPECIES: hypothetical protein [Ensifer] | | | |  |
|  |  | | chromosome 1 | OV14_RS10060 | | WP_025426470.1 | | | >WP_025426470.1 MULTISPECIES: hypothetical protein [Ensifer] | | | |  |
|  |  | | chromosome 1 | OV14_RS10135 | | WP_025426485.1 | | | >WP_025426485.1 MULTISPECIES: membrane protein [Ensifer] | | | |  |
|  |  | | chromosome 1 | OV14_RS14430 | | WP_025427296.1 | | | >WP_025427296.1 MULTISPECIES: TIGR02594 family protein [Ensifer] | | | |  |
|  |  | | chromosome 1 | OV14_RS15210 | | WP_038577107.1 | | | >WP_038577107.1 MULTISPECIES: hydrolase [Ensifer] | | | |  |
|  |  | | chromosome 1 | OV14_RS17390 | | WP_025427872.1 | | | >WP_025427872.1 SIMPL domain-containing protein [Ensifer adhaerens] | | | |  |
|  |  | | chromosome 1 | OV14_RS18190 | | WP_025428029.1 | | | >WP_025428029.1 MULTISPECIES: hypothetical protein [Ensifer] | | | |  |
|  |  | | chromosome 1 | OV14_RS18195 | | WP_003592881.1 | | | Pseudo | | | |  |
|  |  | | chromosome 2 | OV14_RS19920 | | WP_025428361.1 | | | >WP_025428361.1 MULTISPECIES: hypothetical protein [Ensifer] | | | |  |
|  |  | | chromosome 2 | OV14_RS21085 | | WP_051509284.1 | | | >WP_051509284.1 copper-translocating P-type ATPase [Ensifer adhaerens] | | | |  |
|  |  | | chromosome 2 | OV14_RS21090 | | WP_025428583.1 | | | >WP_025428583.1 MULTISPECIES: membrane protein [Ensifer] | | | |  |
|  |  | | chromosome 2 | OV14_RS21095 | | WP_025428584.1 | | | >WP_025428584.1 hypothetical protein [Ensifer adhaerens] | | | |  |
|  |  | | chromosome 2 | OV14_RS22020 | | WP_025428764.1 | | | >WP_025428764.1 MULTISPECIES: hypothetical protein [Ensifer] | | | |  |
|  |  | | chromosome 2 | OV14_RS22440 | | WP_025428846.1 | | | >WP_025428846.1 MULTISPECIES: membrane protein [Ensifer] | | | |  |
|  |  | | chromosome 2 | OV14_RS22445 | | WP_025428847.1 | | | >WP_025428847.1 alkylhydroperoxidase domain protein [Ensifer adhaerens] | | | |  |
|  |  | | chromosome 2 | OV14_RS24525 | | WP_025429243.1 | | | >WP_025429243.1 glutathione S-transferase [Ensifer adhaerens] | | | |  |
|  |  | | chromosome 2 | OV14_RS25005 | | WP_025429336.1 | | | >WP_025429336.1 decarboxylase [Ensifer adhaerens] | | | |  |
|  |  | | chromosome 2 | OV14_RS25010 | | WP_025429337.1 | | | >WP_025429337.1 membrane protein [Ensifer adhaerens] | | | |  |
|  |  | | chromosome 2 | OV14_RS26895 | | WP_025429700.1 | | | >WP_025429700.1 MULTISPECIES: hypothetical protein [Ensifer] | | | |  |
|  |  | | chromosome 2 | OV14_RS27755 | | WP_025429865.1 | | | >WP_025429865.1 MULTISPECIES: hypothetical protein [Ensifer] | | | |  |
|  |  | | chromosome 2 | OV14_RS27775 | | WP_025429869.1 | | | >WP_025429869.1 MULTISPECIES: 3-oxoacyl-ACP reductase [Ensifer] | | | |  |
| D1(BR) D2(BR) D3(BR) D5(BR) D7(BR) | 5 | | chromosome 1 | OV14_RS16135 | | WP_025427631.1 | | | >WP_025427631.1 MULTISPECIES: nucleoside-diphosphate sugar epimerase [Ensifer] | | | |  |
|  |  | | chromosome 2 | OV14_RS21660 | | WP_025428695.1 | | | >WP_025428695.1 MULTISPECIES: dioxygenase [Ensifer] | | | |  |
|  |  | | chromosome 2 | OV14_RS24990 | | WP_025429333.1 | | | >WP_025429333.1 hypothetical protein [Ensifer adhaerens] | | | |  |
|  |  | | chromosome 2 | OV14_RS24995 | | WP_025429334.1 | | | >WP_025429334.1 hypothetical protein [Ensifer adhaerens] | | | |  |
|  |  | | plasmid b | OV14_RS30440 | | WP_025430375.1 | | | >WP_025430375.1 adenylate cyclase [Ensifer adhaerens] | | | |  |

**Down-regulated genes**

| **Day(s)** | **No. genes** | **Location** | **Gene ID** | **Protein ID** | **NCBI annotated function** |
| --- | --- | --- | --- | --- | --- |
| D1(BR) | 47 | chromosome 1 | OV14_RS03685 | WP_025425235.1 | >WP_025425235.1 MULTISPECIES: amino acid ABC transporter substrate-binding protein [Ensifer] |
|  |  | chromosome 1 | OV14_RS06325 | WP_025425745.1 | >WP_025425745.1 MULTISPECIES: fasciclin [Ensifer] |
|  |  | chromosome 1 | OV14_RS06775 | WP_025425831.1 | >WP_025425831.1 MULTISPECIES: Rrf2 family transcriptional regulator [Ensifer] |
|  |  | chromosome 1 | OV14_RS08970 | WP_025426257.1 | >WP_025426257.1 MULTISPECIES: organic hydroperoxide resistance protein [Ensifer] |
|  |  | chromosome 1 | OV14_RS14400 | WP_025427290.1 | >WP_025427290.1 MULTISPECIES: ABC transporter substrate-binding protein [Ensifer] |
|  |  | chromosome 1 | OV14_RS14555 | WP_025427321.1 | >WP_025427321.1 MULTISPECIES: zinc ABC transporter substrate-binding protein [Ensifer] |
|  |  | chromosome 1 | OV14_RS15180 | WP_025427446.1 | >WP_025427446.1 MULTISPECIES: hypothetical protein [Ensifer] |
|  |  | chromosome 1 | OV14_RS18485 | WP_025428084.1 | >WP_025428084.1 MULTISPECIES: hypothetical protein [Ensifer] |
|  |  | chromosome 1 | OV14_RS18490 | WP_025428085.1 | >WP_025428085.1 MULTISPECIES: hypothetical protein [Ensifer] |
|  |  | chromosome 1 | OV14_RS18500 | WP_025428087.1 | >WP_025428087.1 MULTISPECIES: hypothetical protein [Ensifer] |
|  |  | chromosome 2 | OV14_RS19235 | WP_025428228.1 | >WP_025428228.1 SDR family oxidoreductase [Ensifer adhaerens] |
|  |  | chromosome 2 | OV14_RS19240 | WP_038578040.1 | >WP_038578040.1 multidrug MFS transporter [Ensifer adhaerens] |
|  |  | chromosome 2 | OV14_RS20760 | WP_025428517.1 | >WP_025428517.1 hypothetical protein [Ensifer adhaerens] |
|  |  | chromosome 2 | OV14_RS21850 | WP_025428730.1 | >WP_025428730.1 pseudoazurin [Ensifer adhaerens] |
|  |  | chromosome 2 | OV14_RS22800 | WP_051509292.1 | >WP_051509292.1 calcium-binding protein [Ensifer adhaerens] |
|  |  | chromosome 2 | OV14_RS22815 | WP_038578296.1 | >WP_038578296.1 cytochrome P450 [Ensifer adhaerens] |
|  |  | chromosome 2 | OV14_RS22820 | WP_051509235.1 | >WP_051509235.1 hypothetical protein [Ensifer adhaerens] |
|  |  | chromosome 2 | OV14_RS22825 | WP_025428921.1 | >WP_025428921.1 NorE accessory protein for nitric oxide reductase [Ensifer adhaerens] |
|  |  | chromosome 2 | OV14_RS24945 | WP_025429324.1 | >WP_025429324.1 MULTISPECIES: membrane protein [Ensifer] |
|  |  | chromosome 2 | OV14_RS26440 | WP_003540211.1 | Pseudogene |
|  |  | plasmid b | OV14_RS29525 | WP_025430197.1 | >WP_025430197.1 nitrate reductase [Ensifer adhaerens] |
|  |  | plasmid b | OV14_RS29530 | WP_025430198.1 | >WP_025430198.1 nitrite reductase, copper-containing [Ensifer adhaerens] |
|  |  | plasmid b | OV14_RS29535 | WP_025430199.1 | >WP_025430199.1 short-chain dehydrogenase [Ensifer adhaerens] |
|  |  | plasmid b | OV14_RS29565 | WP_025430205.1 | >WP_025430205.1 MULTISPECIES: aminotransferase [Ensifer] |
|  |  | plasmid b | OV14_RS29570 | WP_025430206.1 | >WP_025430206.1 coproporphyrinogen III oxidase [Ensifer adhaerens] |
|  |  | plasmid b | OV14_RS29580 | WP_025430208.1 | >WP_025430208.1 MULTISPECIES: CbbQ/NirQ/NorQ/GpvN family protein [Ensifer] |
|  |  | plasmid b | OV14_RS29585 | WP_025430209.1 | >WP_025430209.1 MULTISPECIES: nitric-oxide reductase large subunit [Ensifer] |
|  |  | plasmid b | OV14_RS29590 | WP_025430210.1 | >WP_025430210.1 MULTISPECIES: cytochrome c [Ensifer] |
|  |  | plasmid b | OV14_RS29600 | WP_025430212.1 | >WP_025430212.1 MULTISPECIES: NorE accessory protein for nitric oxide reductase [Ensifer] |
|  |  | plasmid b | OV14_RS29605 | WP_064742394.1 | >WP_064742394.1 hypothetical protein [Ensifer adhaerens] |
|  |  | plasmid b | OV14_RS29630 | WP_025430218.1 | >WP_025430218.1 protease [Ensifer adhaerens] |
|  |  | plasmid b | OV14_RS29640 | WP_025430220.1 | >WP_025430220.1 MULTISPECIES: hypothetical protein [Ensifer] |
|  |  | plasmid b | OV14_RS29685 | WP_025430229.1 | >WP_025430229.1 ferrichrome ABC transporter substrate-binding protein [Ensifer adhaerens] |
|  |  | plasmid b | OV14_RS30655 | WP_025430417.1 | >WP_025430417.1 hypothetical protein [Ensifer adhaerens] |
|  |  | plasmid b | OV14_RS30660 | WP_025430418.1 | >WP_025430418.1 MULTISPECIES: hypothetical protein [Ensifer] |
|  |  | plasmid b | OV14_RS30665 | WP_025430419.1 | >WP_025430419.1 FMN-binding protein [Ensifer adhaerens] |
|  |  | plasmid b | OV14_RS36595 | WP_025430426.1 | >WP_025430426.1 hypothetical protein [Ensifer adhaerens] |
|  |  | plasmid b | OV14_RS30705 | WP_025430427.1 | >WP_025430427.1 MULTISPECIES: hypothetical protein [Ensifer] |
|  |  | plasmid b | OV14_RS31400 | WP_025430563.1 | >WP_025430563.1 acyl-[ACP]--phospholipid O-acyltransferase [Ensifer adhaerens] |
|  |  | plasmid b | OV14_RS31465 | WP_025430576.1 | >WP_025430576.1 peroxidase [Ensifer adhaerens] |
|  |  | plasmid b | OV14_RS31665 | WP_025430613.1 | Pseudogene |
|  |  | plasmid b | OV14_RS32545 | WP_025430782.1 | >WP_025430782.1 MULTISPECIES: hypothetical protein [Ensifer] |
|  |  | plasmid b | OV14_RS34175 | WP_025431086.1 | >WP_025431086.1 2-polyprenyl-6-methoxyphenol hydroxylase [Ensifer adhaerens] |
|  |  | plasmid b | OV14_RS34185 | WP_025431087.1 | >WP_025431087.1 DSBA oxidoreductase [Ensifer adhaerens] |
|  |  | plasmid b | OV14_RS35325 | WP_025431294.1 | >WP_025431294.1 alpha-glucosidase/alpha-galactosidase [Ensifer adhaerens] |
|  |  | plasmid b | OV14_RS35330 | WP_025431295.1 | >WP_025431295.1 periplasmic alpha-galactoside-binding protein [Ensifer adhaerens] |
|  |  | plasmid b | OV14_RS35345 | WP_025431298.1 | >WP_025431298.1 ABC transporter ATP-binding protein [Ensifer adhaerens] |
| D2(BR) | 19 | chromosome 1 | OV14_RS01620 | WP_025424845.1 | >WP_025424845.1 MULTISPECIES: VWA domain-containing protein [Ensifer] |
|  |  | chromosome 1 | OV14_RS06070 | WP_025425694.1 | >WP_025425694.1 MULTISPECIES: hybrid sensor histidine kinase/response regulator [Ensifer] |
|  |  | chromosome 1 | OV14_RS07160 | WP_025425905.1 | >WP_025425905.1 MULTISPECIES: flagellar M-ring protein FliF [Ensifer] |
|  |  | chromosome 1 | OV14_RS07185 | WP_025425910.1 | >WP_025425910.1 MULTISPECIES: flagellar motor switch protein FliG [Ensifer] |
|  |  | chromosome 1 | OV14_RS07195 | WP_025425912.1 | >WP_025425912.1 MULTISPECIES: flagellar motor switch protein FliM [Ensifer] |
|  |  | chromosome 1 | flgF | WP_025425915.1 | >WP_025425915.1 MULTISPECIES: flagellar basal body rod protein FlgF [Ensifer] |
|  |  | chromosome 1 | OV14_RS07230 | WP_025425919.1 | >WP_025425919.1 MULTISPECIES: flagellar basal body rod protein FlgC [Ensifer] |
|  |  | chromosome 1 | OV14_RS07275 | WP_051509153.1 | >WP_051509153.1 MULTISPECIES: glycosyl transferase [Ensifer] |
|  |  | chromosome 1 | OV14_RS08375 | WP_025426142.1 | >WP_025426142.1 chemotaxis protein [Ensifer adhaerens] |
|  |  | chromosome 1 | OV14_RS08380 | WP_025426143.1 | >WP_025426143.1 chemotaxis protein [Ensifer adhaerens] |
|  |  | chromosome 1 | OV14_RS13485 | WP_025427108.1 | >WP_025427108.1 MULTISPECIES: hypothetical protein [Ensifer] |
|  |  | chromosome 1 | OV14_RS15920 | WP_025427588.1 | >WP_025427588.1 MULTISPECIES: ketol-acid reductoisomerase [Ensifer] |
|  |  | chromosome 1 | OV14_RS16035 | WP_025427611.1 | >WP_025427611.1 MULTISPECIES: rhodanese [Ensifer] |
|  |  | chromosome 1 | OV14_RS16605 | WP_025427722.1 | >WP_025427722.1 MULTISPECIES: aquaporin [Ensifer] |
|  |  | chromosome 2 | OV14_RS20445 | WP_025428459.1 | >WP_025428459.1 MULTISPECIES: methylcrotonoyl-CoA carboxylase [Ensifer] |
|  |  | chromosome 2 | OV14_RS20450 | WP_025428460.1 | >WP_025428460.1 endoribonuclease L-PSP [Ensifer adhaerens] |
|  |  | chromosome 2 | OV14_RS22695 | WP_038577891.1 | >WP_038577891.1 MULTISPECIES: hypothetical protein [Ensifer] |
|  |  | chromosome 2 | OV14_RS23295 | WP_025429002.1 | >WP_025429002.1 xylose isomerase [Ensifer adhaerens] |
|  |  | chromosome 2 | OV14_RS23590 | WP_025429058.1 | >WP_025429058.1 acetyl-CoA carboxylase [Ensifer adhaerens] |
| D3(BR) | 46 | chromosome 1 | OV14_RS01295 | WP_025424783.1 | MULTISPECIES: transcriptional regulator [Ensifer] |
|  |  | chromosome 1 | OV14_RS01720 | WP_025424864.1 | MULTISPECIES: hypothetical protein [Ensifer] |
|  |  | chromosome 1 | OV14_RS03120 | WP_051509187.1 | MULTISPECIES: nicotinate-nicotinamide nucleotide adenylyltransferase [Ensifer] |
|  |  | chromosome 1 | OV14_RS03515 | WP_025425202.1 | MULTISPECIES: ABC transporter permease [Ensifer] |
|  |  | chromosome 1 | OV14_RS04010 | WP_025425299.1 | MULTISPECIES: tRNA modification GTPase [Ensifer] |
|  |  | chromosome 1 | OV14_RS05815 | WP_025425644.1 | MULTISPECIES: RpiR family transcriptional regulator [Ensifer] |
|  |  | chromosome 1 | OV14_RS07205 | WP_025425914.1 | MULTISPECIES: flagellar basal body rod protein FlgF [Ensifer] |
|  |  | chromosome 1 | fliI | WP_025425916.1 | MULTISPECIES: flagellum-specific ATP synthase FliI [Ensifer] |
|  |  | chromosome 1 | OV14_RS07290 | WP_025425931.1 | flagellin [Ensifer adhaerens] |
|  |  | chromosome 1 | OV14_RS07305 | WP_025425932.1 | MULTISPECIES: flagellin [Ensifer] |
|  |  | chromosome 1 | OV14_RS07440 | WP_025425959.1 | MULTISPECIES: transcriptional regulator [Ensifer] |
|  |  | chromosome 1 | OV14_RS07465 | WP_025425964.1 | MULTISPECIES: ABC transporter ATP-binding protein [Ensifer] |
|  |  | chromosome 1 | OV14_RS07485 | WP_025425968.1 | MULTISPECIES: 6-phosphogluconolactonase [Ensifer] |
|  |  | chromosome 1 | OV14_RS07490 | WP_025425969.1 | MULTISPECIES: glucose-6-phosphate dehydrogenase [Ensifer] |
|  |  | chromosome 1 | OV14_RS07695 | WP_025426009.1 | MULTISPECIES: hypothetical protein [Ensifer] |
|  |  | chromosome 1 | OV14_RS09585 | WP_025426376.1 | MULTISPECIES: porin [Ensifer] |
|  |  | chromosome 1 | OV14_RS09755 | WP_025426410.1 | MULTISPECIES: nicotinate-nucleotide diphosphorylase (carboxylating) [Ensifer] |
|  |  | chromosome 1 | OV14_RS10455 | WP_025426544.1 | MULTISPECIES: glycosyl transferase [Ensifer] |
|  |  | chromosome 1 | OV14_RS12370 | WP_025426904.1 | phage recombination protein Bet [Ensifer adhaerens] |
|  |  | chromosome 1 | OV14_RS16250 | WP_025427654.1 | MULTISPECIES: acetolactate synthase 3 large subunit [Ensifer] |
|  |  | chromosome 1 | OV14_RS16280 | WP_025427660.1 | MULTISPECIES: hypothetical protein [Ensifer] |
|  |  | chromosome 1 | OV14_RS17335 | WP_025427862.1 | MULTISPECIES: cobalt transporter [Ensifer] |
|  |  | chromosome 1 | OV14_RS18230 | WP_025428035.1 | MULTISPECIES: hypothetical protein [Ensifer] |
|  |  | chromosome 1 | OV14_RS18325 | WP_025428054.1 | MULTISPECIES: ABC transporter ATP-binding protein [Ensifer] |
|  |  | chromosome 1 | OV14_RS18435 | WP_025428075.1 | peptidase [Ensifer adhaerens] |
|  |  | chromosome 2 | OV14_RS19975 | WP_038578107.1 | MULTISPECIES: hypothetical protein [Ensifer] |
|  |  | chromosome 2 | OV14_RS19985 | WP_025428370.1 | MULTISPECIES: penicillin-binding protein [Ensifer] |
|  |  | chromosome 2 | OV14_RS21780 | WP_025428716.1 | lipoprotein [Ensifer adhaerens] |
|  |  | chromosome 2 | OV14_RS22080 | WP_025428776.1 | oxidoreductase [Ensifer adhaerens] |
|  |  | chromosome 2 | OV14_RS23455 | WP_025429031.1 | multidrug DMT transporter permease [Ensifer adhaerens] |
|  |  | chromosome 2 | OV14_RS24610 | WP_025429260.1 | amidohydrolase [Ensifer adhaerens] |
|  |  | chromosome 2 | OV14_RS25050 | WP_025429345.1 | MULTISPECIES: hypothetical protein [Ensifer] |
|  |  | chromosome 2 | OV14_RS25250 | WP_025429382.1 | MULTISPECIES: ABC transporter permease [Ensifer] |
|  |  | chromosome 2 | OV14_RS25515 | WP_038577971.1 | hypothetical protein [Ensifer adhaerens] |
|  |  | chromosome 2 | OV14_RS26160 | WP_025429554.1 | GntR family transcriptional regulator [Ensifer adhaerens] |
|  |  | chromosome 2 | OV14_RS36840 | WP_038577999.1 | outer membrane autotransporter barrel domain-containing protein [Ensifer adhaerens] |
|  |  | chromosome 2 | OV14_RS28070 | WP_025429927.1 |  |
|  |  | chromosome 2 | OV14_RS28275 | WP_025429968.1 | MULTISPECIES: transcriptional regulator [Ensifer] |
|  |  | chromosome 2 | OV14_RS36840 | WP_038577999.1 | membrane protein [Ensifer adhaerens] |
|  |  | plasmid b | OV14_RS29985 | WP_025430287.1 | MULTISPECIES: ABC transporter permease [Ensifer] |
|  |  | plasmid b | OV14_RS31280 | WP_041692930.1 | alpha/beta hydrolase [Ensifer adhaerens] |
|  |  | plasmid b | OV14_RS32105 | WP_025430700.1 | hypothetical protein [Ensifer adhaerens] |
|  |  | plasmid b | OV14_RS33680 | WP_025430998.1 | hypothetical protein [Ensifer adhaerens] |
|  |  | plasmid b | OV14_RS34905 | WP_025431221.1 | aminotransferase [Ensifer adhaerens] |
|  |  | plasmid b | OV14_RS35380 | WP_025431305.1 | sugar ABC transporter ATP-binding protein [Ensifer adhaerens] |
|  |  | plasmid b | OV14_RS35885 | WP_025431400.1 | FAD-dependent oxidoreductase [Ensifer adhaerens] |
|  |  | plasmid 5105 | virE1 | [ABK01312.1](https://www.ncbi.nlm.nih.gov/protein/116585231) | type IV secretion system chaperone VirE1 |
| D5(BR) | 62 | chromosome 1 | OV14_RS00510 | WP_025424631.1 | hypothetical protein [Ensifer adhaerens] |
|  |  | chromosome 1 | OV14_RS00850 | WP_025424698.1 | MULTISPECIES: hypothetical protein [Ensifer] |
|  |  | chromosome 1 | OV14_RS02445 | WP_025424996.1 | MULTISPECIES: 30S ribosomal protein S21 [Ensifer] |
|  |  | chromosome 1 | OV14_RS04500 | WP_025425390.1 | MULTISPECIES: peptidase [Ensifer] |
|  |  | chromosome 1 | OV14_RS05265 | WP_025425538.1 | MULTISPECIES: methyltransferase [Ensifer] |
|  |  | chromosome 1 | OV14_RS07525 | WP_025425976.1 | MULTISPECIES: LuxR family transcriptional regulator [Ensifer] |
|  |  | chromosome 1 | OV14_RS07615 | WP_025425993.1 | MULTISPECIES: LysR family transcriptional regulator [Ensifer] |
|  |  | chromosome 1 | OV14_RS08015 | WP_025426070.1 | MULTISPECIES: inositol monophosphatase [Ensifer] |
|  |  | chromosome 1 | OV14_RS09220 | WP_051509155.1 | MULTISPECIES: inverse autotransporter beta-barrel domain-containing protein [Ensifer] |
|  |  | chromosome 1 | OV14_RS10450 | WP_025426543.1 | undecaprenyl-phosphate glucose phosphotransferase [Ensifer adhaerens] |
|  |  | chromosome 1 | OV14_RS10500 | WP_025426553.1 | MULTISPECIES: agmatinase [Ensifer] |
|  |  | chromosome 1 | OV14_RS11505 | WP_025426737.1 | hypothetical protein [Ensifer adhaerens] |
|  |  | chromosome 1 | OV14_RS12055 | WP_025426845.1 | MULTISPECIES: cardiolipin synthetase [Ensifer] |
|  |  | chromosome 1 | OV14_RS13960 | WP_025427202.1 | MULTISPECIES: FMN reductase [Ensifer] |
|  |  | chromosome 1 | OV14_RS13985 | WP_025427207.1 | MULTISPECIES: transporter [Ensifer] |
|  |  | chromosome 1 | OV14_RS14125 | WP_025427235.1 | MULTISPECIES: hypothetical protein [Ensifer] |
|  |  | chromosome 1 | OV14_RS14260 | WP_025427262.1 | MULTISPECIES: aspartate-semialdehyde dehydrogenase [Ensifer] |
|  |  | chromosome 1 | OV14_RS14415 | WP_025427293.1 | RDD family protein [Ensifer adhaerens] |
|  |  | chromosome 1 | OV14_RS15320 | WP_025427474.1 | MULTISPECIES: hypothetical protein [Ensifer] |
|  |  | chromosome 1 | OV14_RS15875 | WP_025427580.1 | MULTISPECIES: guanylyl cyclase [Ensifer] |
|  |  | chromosome 1 | OV14_RS15880 | | Pseudogene |
|  |  | chromosome 1 | OV14_RS17490 | WP_025427891.1 | MULTISPECIES: glutamyl-tRNA amidotransferase [Ensifer] |
|  |  | chromosome 1 | OV14_RS18160 | WP_025428023.1 | MULTISPECIES: photosystem reaction center subunit H [Ensifer] |
|  |  | chromosome 1 | OV14_RS18165 | WP_025428024.1 | MULTISPECIES: D-amino-acid oxidase [Ensifer] |
|  |  | chromosome 1 | OV14_RS18225 | WP_025428034.1 | MULTISPECIES: hypothetical protein [Ensifer] |
|  |  | chromosome 1 | OV14_RS18365 | WP_038577375.1 | MULTISPECIES: adenylate cyclase [Ensifer] |
|  |  | chromosome 1 | OV14_RS18540 | WP_025428095.1 | MULTISPECIES: histidine kinase [Ensifer] |
|  |  | chromosome 2 | OV14_RS19195 | WP_025428220.1 | hypothetical protein [Ensifer adhaerens] |
|  |  | chromosome 2 | OV14_RS22225 | WP_051509290.1 | cyclase [Ensifer adhaerens] |
|  |  | chromosome 2 | OV14_RS22865 | WP_025428929.1 | MULTISPECIES: hypothetical protein [Ensifer] |
|  |  | chromosome 2 | OV14_RS23075 | WP_025428964.1 | hypothetical protein [Ensifer adhaerens] |
|  |  | chromosome 2 | OV14_RS23095 | WP_025428968.1 | hypothetical protein [Ensifer adhaerens] |
|  |  | chromosome 2 | OV14_RS23540 | WP_025429048.1 | death-on-curing family protein [Ensifer adhaerens] |
|  |  | chromosome 2 | OV14_RS24255 | WP_025429191.1 | MULTISPECIES: membrane protein [Ensifer] |
|  |  | chromosome 2 | OV14_RS25130 | WP_051509252.1 | MULTISPECIES: hypothetical protein [Ensifer] |
|  |  | chromosome 2 | OV14_RS25310 | WP_025429394.1 | MULTISPECIES: antibiotic biosynthesis monooxygenase [Ensifer] |
|  |  | chromosome 2 | OV14_RS26205 | WP_025429563.1 | 3-oxoadipate enol-lactonase [Ensifer adhaerens] |
|  |  | chromosome 2 | OV14_RS36530 | WP_051509260.1 | hypothetical protein [Ensifer adhaerens] |
|  |  | chromosome 2 | OV14_RS27065 | WP_025429732.1 | MULTISPECIES: hypothetical protein [Ensifer] |
|  |  | chromosome 2 | OV14_RS28040 | WP_025429921.1 | 5-dehydro-4-deoxyglucarate dehydratase [Ensifer adhaerens] |
|  |  | plasmid b | OV14_RS30245 | WP_025430037.1 | hypothetical protein [Ensifer adhaerens] |
|  |  | plasmid b | OV14_RS30875 | WP_025430460.1 | deaminase/reductase [Ensifer adhaerens] |
|  |  | plasmid b | OV14_RS30970 | WP_025430477.1 | Octopine catabolism/uptake operon regulatory protein occR [Ensifer adhaerens] |
|  |  | plasmid b | OV14_RS32135 | WP_025430705.1 | hypothetical protein [Ensifer adhaerens] |
|  |  | plasmid b | OV14_RS32225 | WP_025430722.1 | RNA polymerase sigma factor [Ensifer adhaerens] |
|  |  | plasmid b | OV14_RS32230 | WP_025430723.1 | hypothetical protein [Ensifer adhaerens] |
|  |  | plasmid b | OV14_RS32250 | WP_025430727.1 | hypothetical protein [Ensifer adhaerens] |
|  |  | plasmid b | OV14_RS32255 | WP_025430728.1 | hypothetical protein [Ensifer adhaerens] |
|  |  | plasmid b | OV14_RS32260 | WP_025430729.1 | hypothetical protein [Ensifer adhaerens] |
|  |  | plasmid b | OV14_RS32810 | WP_025430832.1 | hypothetical protein [Ensifer adhaerens] |
|  |  | plasmid b | OV14_RS32830 | WP_025430836.1 | hypothetical protein [Ensifer adhaerens] |
|  |  | plasmid b | OV14_RS32860 | WP_025430842.1 | hypothetical protein [Ensifer adhaerens] |
|  |  | plasmid b | OV14_RS33615 | WP_025430985.1 | ABC transporter permease [Ensifer adhaerens] |
|  |  | plasmid b | OV14_RS34055 | WP_025431063.1 | MULTISPECIES: hypothetical protein [Ensifer] |
|  |  | plasmid b | OV14_RS34210 | WP_025431092.1 | aldehyde dehydrogenase [Ensifer adhaerens] |
|  |  | plasmid b | OV14_RS34575 | WP_041693144.1 | hypothetical protein [Ensifer adhaerens] |
|  |  | plasmid b | OV14_RS35615 | WP_025431351.1 | oxidoreductase [Ensifer adhaerens] |
|  |  | plasmid b | OV14_RS36100 | WP_025431440.1 | MULTISPECIES: histidine kinase [Ensifer] |
|  |  | plasmid b | OV14_RS36210 | WP_025431461.1 | penicillin-binding protein [Ensifer adhaerens] |
|  |  | plasmid c | OV14_RS28605 | WP_081789025.1 | hypothetical protein |
|  |  | plasmid c | OV14_RS28660 | WP_025430337.1 | MULTISPECIES: ABC transporter ATP-binding protein [Ensifer] |
| D7(BR) | 316 | chromosome 1 | OV14_RS00030 | WP_025424541.1 | MULTISPECIES: ABC transporter [Ensifer] |
|  |  | chromosome 1 | OV14_RS00060 | WP_025424546.1 | MULTISPECIES: SAM-dependent methyltransferase [Ensifer] |
|  |  | chromosome 1 | OV14_RS00140 | WP_038575631.1 | MULTISPECIES: multi antimicrobial extrusion protein MatE [Ensifer] |
|  |  | chromosome 1 | OV14_RS00195 | WP_038575638.1 | hypothetical protein [Ensifer adhaerens] |
|  |  | chromosome 1 | OV14_RS00255 | WP_025424583.1 | hypothetical protein [Ensifer adhaerens] |
|  |  | chromosome 1 | OV14_RS00300 | WP_025424592.1 | terminase [Ensifer adhaerens] |
|  |  | chromosome 1 | OV14_RS00480 | WP_025424626.1 | MULTISPECIES: membrane protein [Ensifer] |
|  |  | chromosome 1 | OV14_RS00485 | WP_025424627.1 | MULTISPECIES: cob(I)yrinic acid a,c-diamide adenosyltransferase [Ensifer] |
|  |  | chromosome 1 | OV14_RS00995 | WP_038576464.1 | MULTISPECIES: tRNA(Ile)-lysidine synthetase [Ensifer] |
|  |  | chromosome 1 | OV14_RS01135 | WP_025424752.1 | MULTISPECIES: 5-formyltetrahydrofolate cyclo-ligase [Ensifer] |
|  |  | chromosome 1 | OV14_RS01145 | WP_025424754.1 | hypothetical protein [Ensifer adhaerens] |
|  |  | chromosome 1 | OV14_RS01225 | WP_025424769.1 | MULTISPECIES: adenylate cyclase [Ensifer] |
|  |  | chromosome 1 | OV14_RS01520 | WP_025424826.1 | MULTISPECIES: peroxiredoxin [Ensifer] |
|  |  | chromosome 1 | OV14_RS01555 | WP_025424833.1 | MULTISPECIES: hypothetical protein [Ensifer] |
|  |  | chromosome 1 | OV14_RS01570 | WP_025424836.1 | MULTISPECIES: MarR family transcriptional regulator [Ensifer] |
|  |  | chromosome 1 | OV14_RS01590 | WP_063963233.1 | MULTISPECIES: hypothetical protein [Ensifer] |
|  |  | chromosome 1 | OV14_RS01615 | WP_025424844.1 | MULTISPECIES: transglutaminase [Ensifer] |
|  |  | chromosome 1 | OV14_RS01790 | WP_025424877.1 | MULTISPECIES: metal transporter [Ensifer] |
|  |  | chromosome 1 | OV14_RS02215 | WP_025424959.1 | MULTISPECIES: N-acetylglucosaminyltransferase [Ensifer] |
|  |  | chromosome 1 | OV14_RS02365 | WP_025424983.1 | MULTISPECIES: choline dehydrogenase [Ensifer] |
|  |  | chromosome 1 | OV14_RS02510 | WP_025425008.1 | MULTISPECIES: dihydroorotase [Ensifer] |
|  |  | chromosome 1 | OV14_RS02640 | WP_025425034.1 | MULTISPECIES: formate dehydrogenase subunit alpha [Ensifer] |
|  |  | chromosome 1 | OV14_RS02645 | WP_025425035.1 | MULTISPECIES: formate dehydrogenase [Ensifer] |
|  |  | chromosome 1 | OV14_RS02730 | WP_025425051.1 | MULTISPECIES: membrane protein [Ensifer] |
|  |  | chromosome 1 | OV14_RS02760 | | Pseudogene |
|  |  | chromosome 1 | OV14_RS03015 | WP_038575751.1 | MULTISPECIES: membrane protein [Ensifer] |
|  |  | chromosome 1 | OV14_RS03180 | WP_025425137.1 | MULTISPECIES: Zn-dependent protease [Ensifer] |
|  |  | chromosome 1 | OV14_RS03460 | WP_025425191.1 | MULTISPECIES: GCN5 family acetyltransferase [Ensifer] |
|  |  | chromosome 1 | OV14_RS03570 | WP_025425212.1 | MULTISPECIES: thiamine/thiamine pyrophosphate ABC transporter permease ThiP [Ensifer] |
|  |  | chromosome 1 | OV14_RS03760 | WP_025425250.1 | MULTISPECIES: aspartate aminotransferase family protein [Ensifer] |
|  |  | chromosome 1 | OV14_RS04345 | WP_025425366.1 | MULTISPECIES: malate synthase G [Ensifer] |
|  |  | chromosome 1 | OV14_RS04425 | WP_025425375.1 | MULTISPECIES: ATPase [Ensifer] |
|  |  | chromosome 1 | OV14_RS04430 | WP_025425376.1 | MULTISPECIES: heme ABC transporter ATP-binding protein [Ensifer] |
|  |  | chromosome 1 | OV14_RS04440 | WP_025425378.1 | ABC transporter permease [Ensifer adhaerens] |
|  |  | chromosome 1 | OV14_RS04645 | WP_025425419.1 | MULTISPECIES: hypothetical protein [Ensifer] |
|  |  | chromosome 1 | OV14_RS04695 | WP_025425429.1 | MULTISPECIES: metal-binding protein [Ensifer] |
|  |  | chromosome 1 | OV14_RS04810 | WP_025425452.1 | MULTISPECIES: N-acetylmuramic acid 6-phosphate etherase [Ensifer] |
|  |  | chromosome 1 | OV14_RS04815 | WP_025425453.1 | MULTISPECIES: N-acetylglucosamine kinase [Ensifer] |
|  |  | chromosome 1 | OV14_RS04905 | WP_064742367.1 | chloramphenicol efflux MFS transporter [Ensifer adhaerens] |
|  |  | chromosome 1 | OV14_RS04990 | WP_025425486.1 | MULTISPECIES: ribosome maturation factor [Ensifer] |
|  |  | chromosome 1 | OV14_RS05370 | WP_025425558.1 | MULTISPECIES: transcriptional regulator [Ensifer] |
|  |  | chromosome 1 | OV14_RS05750 | WP_025425631.1 | MULTISPECIES: AsnC family transcriptional regulator [Ensifer] |
|  |  | chromosome 1 | OV14_RS05760 | WP_025425633.1 | MULTISPECIES: peptide chain release factor 3 [Ensifer] |
|  |  | chromosome 1 | OV14_RS05845 | WP_025425650.1 | MULTISPECIES: ABC transporter [Ensifer] |
|  |  | chromosome 1 | OV14_RS05915 | WP_038575838.1 | MULTISPECIES: transcriptional regulator [Ensifer] |
|  |  | chromosome 1 | OV14_RS06115 | WP_025425703.1 | MULTISPECIES: iron transporter [Ensifer] |
|  |  | chromosome 1 | OV14_RS06120 | WP_025425704.1 | MULTISPECIES: tRNA (adenosine(37)-N6)-threonylcarbamoyltransferase complex dimerization subunit type 1 TsaB [Ensifer] |
|  |  | chromosome 1 | OV14_RS06130 | WP_025425706.1 | MULTISPECIES: 1-acyl-sn-glycerol-3-phosphate acyltransferase [Ensifer] |
|  |  | chromosome 1 | OV14_RS06170 | WP_025425714.1 | MULTISPECIES: tRNA (guanine(46)-N(7))-methyltransferase [Ensifer] |
|  |  | chromosome 1 | OV14_RS06210 | WP_025425722.1 | MULTISPECIES: methionyl-tRNA formyltransferase [Ensifer] |
|  |  | chromosome 1 | OV14_RS06355 | WP_025425751.1 | MULTISPECIES: chemotaxis protein [Ensifer] |
|  |  | chromosome 1 | OV14_RS06390 | WP_025425757.1 | MULTISPECIES: calpastatin [Ensifer] |
|  |  | chromosome 1 | OV14_RS06500 | WP_025425778.1 | MULTISPECIES: hypothetical protein [Ensifer] |
|  |  | chromosome 1 | OV14_RS06565 | WP_025425790.1 | MULTISPECIES: hypothetical protein [Ensifer] |
|  |  | chromosome 1 | OV14_RS06690 | WP_025425813.1 | MULTISPECIES: DNA-binding protein [Ensifer] |
|  |  | chromosome 1 | OV14_RS07070 | WP_025425887.1 | MULTISPECIES: DeoR family transcriptional regulator [Ensifer] |
|  |  | chromosome 1 | OV14_RS07535 | WP_025425978.1 | MULTISPECIES: isocitrate lyase [Ensifer] |
|  |  | chromosome 1 | OV14_RS07540 | WP_025425979.1 | MULTISPECIES: Cro/Cl family transcriptional regulator [Ensifer] |
|  |  | chromosome 1 | rirA | WP_025425998.1 | MULTISPECIES: iron-responsive transcriptional regulator [Ensifer] |
|  |  | chromosome 1 | OV14_RS07665 | WP_025426003.1 | MULTISPECIES: peptide transporter [Ensifer] |
|  |  | chromosome 1 | OV14_RS07805 | WP_025426030.1 | MULTISPECIES: zinc-dependent alcohol dehydrogenase [Ensifer] |
|  |  | chromosome 1 | OV14_RS08035 | WP_038575925.1 | MULTISPECIES: ArsR family transcriptional regulator [Ensifer] |
|  |  | chromosome 1 | OV14_RS08040 | WP_025426075.1 | MULTISPECIES: tetraacyldisaccharide 4'-kinase [Ensifer] |
|  |  | chromosome 1 | OV14_RS08050 | WP_025426077.1 | MULTISPECIES: DNA mismatch repair protein MutL [Ensifer] |
|  |  | chromosome 1 | OV14_RS08060 | WP_038576714.1 | GGDEF-domain containing protein [Ensifer adhaerens] |
|  |  | chromosome 1 | OV14_RS08070 | WP_025426081.1 | MULTISPECIES: trimethylamine methyltransferase [Ensifer] |
|  |  | chromosome 1 | OV14_RS08085 | WP_025426084.1 | MULTISPECIES: hypothetical protein [Ensifer] |
|  |  | chromosome 1 | OV14_RS08855 | WP_025426234.1 | MULTISPECIES: hypothetical protein [Ensifer] |
|  |  | chromosome 1 | OV14_RS08895 | WP_025426242.1 | MULTISPECIES: DeoR family transcriptional regulator [Ensifer] |
|  |  | chromosome 1 | OV14_RS08965 | WP_025426256.1 | MULTISPECIES: transcriptional regulator [Ensifer] |
|  |  | chromosome 1 | OV14_RS09340 | WP_025426330.1 | MULTISPECIES: hypothetical protein [Ensifer] |
|  |  | chromosome 1 | OV14_RS09465 | WP_025426355.1 | MULTISPECIES: MFS transporter [Ensifer] |
|  |  | chromosome 1 | OV14_RS09525 | WP_025426365.1 | MULTISPECIES: membrane protein [Ensifer] |
|  |  | chromosome 1 | OV14_RS09715 | WP_025426402.1 | membrane protein [Ensifer adhaerens] |
|  |  | chromosome 1 | OV14_RS09810 | WP_025426421.1 | MULTISPECIES: cysteine--tRNA ligase [Ensifer] |
|  |  | chromosome 1 | OV14_RS09855 | WP_025426430.1 | MULTISPECIES: peptidoglycan-binding protein LysM [Ensifer] |
|  |  | chromosome 1 | OV14_RS10095 | WP_038575989.1 | MULTISPECIES: hypothetical protein [Ensifer] |
|  |  | chromosome 1 | OV14_RS10100 | WP_025426478.1 | MULTISPECIES: hypothetical protein [Ensifer] |
|  |  | chromosome 1 | OV14_RS10105 | WP_025426479.1 | MULTISPECIES: branched-chain amino acid ABC transporter permease [Ensifer] |
|  |  | chromosome 1 | OV14_RS10750 | WP_025426599.1 | MULTISPECIES: membrane protein [Ensifer] |
|  |  | chromosome 1 | OV14_RS11500 | WP_025426736.1 | MULTISPECIES: hypothetical protein [Ensifer] |
|  |  | chromosome 1 | OV14_RS11555 | WP_051509203.1 | MULTISPECIES: polyisoprenoid-binding protein [Ensifer] |
|  |  | chromosome 1 | OV14_RS11560 | WP_025426748.1 | MULTISPECIES: cytochrome b [Ensifer] |
|  |  | chromosome 1 | OV14_RS11570 | WP_051509161.1 | MULTISPECIES: hypothetical protein [Ensifer] |
|  |  | chromosome 1 | OV14_RS12420 | WP_025426914.1 | hypothetical protein [Ensifer adhaerens] |
|  |  | chromosome 1 | OV14_RS12585 | WP_051509164.1 | hypothetical protein [Ensifer adhaerens] |
|  |  | chromosome 1 | OV14_RS13025 | WP_025427024.1 | MULTISPECIES: hypothetical protein [Ensifer] |
|  |  | chromosome 1 | OV14_RS13245 | WP_025427063.1 | MULTISPECIES: phosphoribosylaminoimidazolesuccinocarboxamide synthase [Ensifer] |
|  |  | chromosome 1 | OV14_RS13255 | | Pseudogene |
|  |  | chromosome 1 | OV14_RS13335 | WP_038576103.1 | MULTISPECIES: hypothetical protein [Ensifer] |
|  |  | chromosome 1 | OV14_RS13385 | WP_025427089.1 | MULTISPECIES: transcriptional regulator [Ensifer] |
|  |  | chromosome 1 | OV14_RS13465 | WP_025427105.1 | MULTISPECIES: hypothetical protein [Ensifer] |
|  |  | chromosome 1 | OV14_RS13555 | WP_025427122.1 | MULTISPECIES: cysteine desulfurase [Ensifer] |
|  |  | chromosome 1 | OV14_RS13675 | WP_025427146.1 | MULTISPECIES: ATP-binding protein [Ensifer] |
|  |  | chromosome 1 | OV14_RS13945 | WP_025427199.1 | MULTISPECIES: multidrug ABC transporter ATP-binding protein [Ensifer] |
|  |  | chromosome 1 | OV14_RS13955 | WP_025427201.1 | tRNA methyltransferase [Ensifer adhaerens] |
|  |  | chromosome 1 | OV14_RS13965 | WP_025427203.1 | MULTISPECIES: redox-sensitive transcriptional activator SoxR [Ensifer] |
|  |  | chromosome 1 | OV14_RS14190 | WP_025427248.1 | MULTISPECIES: hydrolase [Ensifer] |
|  |  | chromosome 1 | OV14_RS14220 | WP_025427254.1 | MULTISPECIES: membrane protein [Ensifer] |
|  |  | chromosome 1 | OV14_RS14325 | WP_025427275.1 | MULTISPECIES: adenosylcobinamide-GDP ribazoletransferase [Ensifer] |
|  |  | chromosome 1 | OV14_RS14565 | WP_025427323.1 | MULTISPECIES: membrane protein [Ensifer] |
|  |  | chromosome 1 | OV14_RS14675 | WP_025427344.1 | sodium:alanine symporter [Ensifer adhaerens] |
|  |  | chromosome 1 | OV14_RS14755 | WP_025427360.1 | MULTISPECIES: acyl carrier protein [Ensifer] |
|  |  | chromosome 1 | OV14_RS14760 | WP_025427361.1 | MULTISPECIES: coproporphyrinogen III oxidase [Ensifer] |
|  |  | chromosome 1 | OV14_RS15025 | WP_025427414.1 | MULTISPECIES: transcriptional regulator [Ensifer] |
|  |  | chromosome 1 | OV14_RS15090 | WP_025427428.1 | MULTISPECIES: adenylate cyclase [Ensifer] |
|  |  | chromosome 1 | OV14_RS15095 | WP_025427429.1 | MULTISPECIES: hypothetical protein [Ensifer] |
|  |  | chromosome 1 | OV14_RS15185 | WP_025427447.1 | MULTISPECIES: transcriptional regulator [Ensifer] |
|  |  | chromosome 1 | OV14_RS15190 | WP_025427448.1 | MULTISPECIES: short-chain dehydrogenase/reductase [Ensifer] |
|  |  | chromosome 1 | OV14_RS15220 | WP_025427454.1 | MULTISPECIES: hypothetical protein [Ensifer] |
|  |  | chromosome 1 | OV14_RS15225 | WP_025427455.1 | MULTISPECIES: LysR family transcriptional regulator [Ensifer] |
|  |  | chromosome 1 | OV14_RS15335 | WP_038577126.1 | MULTISPECIES: hydrolase [Ensifer] |
|  |  | chromosome 1 | OV14_RS15420 | WP_025427494.1 | hypothetical protein [Ensifer adhaerens] |
|  |  | chromosome 1 | OV14_RS15505 | WP_038576209.1 | MULTISPECIES: TetR family transcriptional regulator [Ensifer] |
|  |  | chromosome 1 | OV14_RS15510 | WP_025427510.1 | MULTISPECIES: hypothetical protein [Ensifer] |
|  |  | chromosome 1 | OV14_RS15515 | WP_025427511.1 | MULTISPECIES: hypothetical protein [Ensifer] |
|  |  | chromosome 1 | OV14_RS15675 | WP_025427540.1 | tetracycline resistance MFS efflux pump [Ensifer adhaerens] |
|  |  | chromosome 1 | OV14_RS15750 | WP_038577186.1 | MULTISPECIES: MFS transporter [Ensifer] |
|  |  | chromosome 1 | OV14_RS15775 | WP_025427560.1 | GntR family transcriptional regulator [Ensifer adhaerens] |
|  |  | chromosome 1 | OV14_RS15870 | WP_025427579.1 | guanylyl cyclase [Ensifer adhaerens] |
|  |  | chromosome 1 | OV14_RS15980 | WP_025427600.1 | MULTISPECIES: hypothetical protein [Ensifer] |
|  |  | chromosome 1 | OV14_RS15985 | WP_038577218.1 | hypothetical protein [Ensifer adhaerens] |
|  |  | chromosome 1 | OV14_RS16030 | WP_051509172.1 | MULTISPECIES: transcriptional regulator FtrA [Ensifer] |
|  |  | chromosome 1 | OV14_RS16265 | WP_025427657.1 | MULTISPECIES: hypothetical protein [Ensifer] |
|  |  | chromosome 1 | OV14_RS16275 | WP_025427659.1 | MULTISPECIES: ATPase [Ensifer] |
|  |  | chromosome 1 | OV14_RS16290 | WP_025427662.1 | MULTISPECIES: Ion channel [Ensifer] |
|  |  | chromosome 1 | OV14_RS16400 | | Pseudogene |
|  |  | chromosome 1 | OV14_RS16450 | WP_025427693.1 | MULTISPECIES: phosphoribosyltransferase [Ensifer] |
|  |  | chromosome 1 | OV14_RS16535 | WP_025427708.1 | MULTISPECIES: 3-methyl-2-oxobutanoate hydroxymethyltransferase [Ensifer] |
|  |  | chromosome 1 | OV14_RS16775 | WP_025427755.1 | MULTISPECIES: membrane protein [Ensifer] |
|  |  | chromosome 1 | OV14_RS16780 | WP_025427756.1 | MULTISPECIES: thiamine pyrophosphate-binding protein [Ensifer] |
|  |  | chromosome 1 | OV14_RS16965 | WP_025427793.1 | MULTISPECIES: hypothetical protein [Ensifer] |
|  |  | chromosome 1 | OV14_RS17000 | WP_025427800.1 | MULTISPECIES: SAM-dependent methyltransferase [Ensifer] |
|  |  | chromosome 1 | OV14_RS17045 | WP_025427809.1 | MULTISPECIES: NADPH dependent quinone reductase [Ensifer] |
|  |  | chromosome 1 | OV14_RS17050 | WP_025427810.1 | MULTISPECIES: carnitine 3-dehydrogenase [Ensifer] |
|  |  | chromosome 1 | OV14_RS17055 | WP_025427811.1 | MULTISPECIES: acyl-CoA dehydrogenase [Ensifer] |
|  |  | chromosome 1 | OV14_RS17275 | WP_025427853.1 | MULTISPECIES: methionine gamma-lyase [Ensifer] |
|  |  | chromosome 1 | OV14_RS17305 | WP_025427856.1 | MULTISPECIES: transcriptional regulator [Ensifer] |
|  |  | chromosome 1 | OV14_RS17420 | WP_025427878.1 | MULTISPECIES: membrane protein [Ensifer] |
|  |  | chromosome 1 | OV14_RS17710 | WP_025427934.1 | MULTISPECIES: ABC transporter substrate-binding protein [Ensifer] |
|  |  | chromosome 1 | OV14_RS17720 | WP_025427936.1 | acetyltransferase [Ensifer adhaerens] |
|  |  | chromosome 1 | OV14_RS17735 | WP_025427939.1 | MULTISPECIES: hypothetical protein [Ensifer] |
|  |  | chromosome 1 | OV14_RS17755 | WP_025427943.1 | MULTISPECIES: adenine phosphoribosyltransferase [Ensifer] |
|  |  | chromosome 1 | OV14_RS17870 | WP_025427965.1 | MULTISPECIES: hypothetical protein [Ensifer] |
|  |  | chromosome 1 | OV14_RS17880 | WP_025427967.1 | MULTISPECIES: hypothetical protein [Ensifer] |
|  |  | chromosome 1 | OV14_RS18215 | WP_025428032.1 | MULTISPECIES: LysR family transcriptional regulator [Ensifer] |
|  |  | chromosome 1 | OV14_RS18460 | WP_025428080.1 | MULTISPECIES: transporter [Ensifer] |
|  |  | chromosome 1 | OV14_RS18645 | WP_038577385.1 | MULTISPECIES: Zn-dependent hydrolase [Ensifer] |
|  |  | chromosome 1 | OV14_RS18650 | WP_025428117.1 | MULTISPECIES: dihydropyrimidinase [Ensifer] |
|  |  | chromosome 1 | OV14_RS18780 | WP_025428143.1 | MULTISPECIES: hypothetical protein [Ensifer] |
|  |  | chromosome 1 | OV14_RS18900 | WP_025428167.1 | MULTISPECIES: ABC transporter permease [Ensifer] |
|  |  | chromosome 1 | OV14_RS18915 | WP_025428170.1 | MULTISPECIES: LysR family transcriptional regulator [Ensifer] |
|  |  | chromosome 1 | OV14_RS19005 | WP_025428188.1 | MULTISPECIES: hypothetical protein [Ensifer] |
|  |  | chromosome 1 | OV14_RS19015 | WP_025428190.1 | MULTISPECIES: hypothetical protein [Ensifer] |
|  |  | chromosome 1 | OV14_RS19045 | WP_025428196.1 | MULTISPECIES: cytochrome-c oxidase [Ensifer] |
|  |  | chromosome 1 | OV14_RS19110 | WP_025428209.1 | MULTISPECIES: LuxR family transcriptional regulator [Ensifer] |
|  |  | chromosome 2 | OV14_RS19230 | WP_025428227.1 | hypothetical protein [Ensifer adhaerens] |
|  |  | chromosome 2 | OV14_RS19265 | WP_064742376.1 | calcium-binding protein [Ensifer adhaerens] |
|  |  | chromosome 2 | OV14_RS19295 | WP_025428239.1 | hypothetical protein [Ensifer adhaerens] |
|  |  | chromosome 2 | OV14_RS19310 | WP_025428242.1 | MULTISPECIES: sugar ABC transporter permease [Ensifer] |
|  |  | chromosome 2 | OV14_RS19430 | WP_025428264.1 | MULTISPECIES: glycosyl hydrolase [Ensifer] |
|  |  | chromosome 2 | OV14_RS19605 | WP_025428299.1 | glycosyl hydrolase [Ensifer adhaerens] |
|  |  | chromosome 2 | OV14_RS20065 | WP_025428386.1 | MULTISPECIES: multidrug ABC transporter ATPase [Ensifer] |
|  |  | chromosome 2 | OV14_RS20300 | WP_025428430.1 | MULTISPECIES: inositol monophosphatase [Ensifer] |
|  |  | chromosome 2 | OV14_RS20305 | WP_025428431.1 | MULTISPECIES: ABC transporter ATP-binding protein [Ensifer] |
|  |  | chromosome 2 | OV14_RS20570 | WP_025428481.1 | LysR family transcriptional regulator [Ensifer adhaerens] |
|  |  | chromosome 2 | OV14_RS20705 | WP_025428506.1 | MULTISPECIES: peptide ABC transporter [Ensifer] |
|  |  | chromosome 2 | OV14_RS20865 | WP_025428538.1 | MULTISPECIES: cyclic nucleotide-binding protein [Ensifer] |
|  |  | chromosome 2 | OV14_RS20880 | WP_025428541.1 | MULTISPECIES: asparagine synthetase B [Ensifer] |
|  |  | chromosome 2 | OV14_RS21255 | WP_051509285.1 | MULTISPECIES: nickel ABC transporter permease [Ensifer] |
|  |  | chromosome 2 | OV14_RS21280 | WP_025428620.1 | MULTISPECIES: 2-deoxyribose-5-phosphate aldolase [Ensifer] |
|  |  | chromosome 2 | OV14_RS21500 | WP_025428663.1 | MULTISPECIES: MFS transporter [Ensifer] |
|  |  | chromosome 2 | OV14_RS21515 | WP_025428666.1 | MULTISPECIES: histidine ammonia-lyase [Ensifer] |
|  |  | chromosome 2 | OV14_RS21520 | WP_025428667.1 | imidazolonepropionase [Ensifer adhaerens] |
|  |  | chromosome 2 | OV14_RS21590 | WP_025428681.1 | aspartate aminotransferase family protein [Ensifer adhaerens] |
|  |  | chromosome 2 | OV14_RS21880 | WP_025428736.1 | MULTISPECIES: IclR family transcriptional regulator [Ensifer] |
|  |  | chromosome 2 | OV14_RS22035 | WP_025428767.1 | hypothetical protein [Ensifer adhaerens] |
|  |  | chromosome 2 | OV14_RS22040 | WP_025428768.1 | BON domain-containing protein [Ensifer adhaerens] |
|  |  | chromosome 2 | OV14_RS22305 | WP_025428819.1 | MULTISPECIES: transcriptional regulator [Ensifer] |
|  |  | chromosome 2 | OV14_RS22425 | WP_025428843.1 | nucleoside 2-deoxyribosyltransferase [Ensifer adhaerens] |
|  |  | chromosome 2 | OV14_RS22660 | WP_025428889.1 | hypothetical protein [Ensifer adhaerens] |
|  |  | chromosome 2 | OV14_RS22785 | WP_025428913.1 | MULTISPECIES: glycerol-3-phosphate transporter permease [Ensifer] |
|  |  | chromosome 2 | OV14_RS23180 | WP_025428980.1 | hypothetical protein [Ensifer adhaerens] |
|  |  | chromosome 2 | OV14_RS23315 | WP_025429006.1 | adenylate cyclase [Ensifer adhaerens] |
|  |  | chromosome 2 | OV14_RS23550 | WP_025429050.1 | LysR family transcriptional regulator [Ensifer adhaerens] |
|  |  | chromosome 2 | OV14_RS23620 | WP_025429064.1 | hypothetical protein [Ensifer adhaerens] |
|  |  | chromosome 2 | OV14_RS23940 | WP_025429127.1 | superoxide dismutase [Ensifer adhaerens] |
|  |  | chromosome 2 | OV14_RS24080 | WP_025429155.1 | MULTISPECIES: ABC transporter permease [Ensifer] |
|  |  | chromosome 2 | OV14_RS24085 | WP_025429156.1 | sugar ABC transporter permease [Ensifer adhaerens] |
|  |  | chromosome 2 | OV14_RS24090 | WP_025429157.1 | ABC transporter substrate-binding protein [Ensifer adhaerens] |
|  |  | chromosome 2 | OV14_RS24200 | WP_025429179.1 | MULTISPECIES: GntR family transcriptional regulator [Ensifer] |
|  |  | chromosome 2 | OV14_RS24210 | WP_025429181.1 | aspartate aminotransferase family protein [Ensifer adhaerens] |
|  |  | chromosome 2 | OV14_RS36510 | WP_025429182.1 | hypothetical protein [Ensifer adhaerens] |
|  |  | chromosome 2 | OV14_RS24535 | WP_025429245.1 | MULTISPECIES: hypothetical protein [Ensifer] |
|  |  | chromosome 2 | OV14_RS24640 | WP_025429266.1 | glycerate kinase [Ensifer adhaerens] |
|  |  | chromosome 2 | OV14_RS24675 | WP_025429273.1 | MULTISPECIES: sugar ABC transporter ATP-binding protein [Ensifer] |
|  |  | chromosome 2 | OV14_RS24685 | WP_025429275.1 | MULTISPECIES: membrane protein [Ensifer] |
|  |  | chromosome 2 | OV14_RS24755 | WP_025429288.1 | MULTISPECIES: ABC transporter permease [Ensifer] |
|  |  | chromosome 2 | OV14_RS24810 | WP_025429299.1 | MULTISPECIES: ABC transporter permease [Ensifer] |
|  |  | chromosome 2 | OV14_RS24815 | WP_025429300.1 | D-ribose transporter ATP-binding protein [Ensifer adhaerens] |
|  |  | chromosome 2 | OV14_RS24820 | WP_025429301.1 | MULTISPECIES: transporter [Ensifer] |
|  |  | chromosome 2 | OV14_RS24880 | WP_051509251.1 | hypothetical protein [Ensifer adhaerens] |
|  |  | chromosome 2 | OV14_RS25230 | WP_025429378.1 | Rrf2 family transcriptional regulator [Ensifer adhaerens] |
|  |  | chromosome 2 | OV14_RS25255 | WP_025429383.1 | L-dehydroascorbate transporter large permease subunit [Ensifer adhaerens] |
|  |  | chromosome 2 | OV14_RS25320 | WP_025429396.1 | MULTISPECIES: TonB-dependent receptor [Ensifer] |
|  |  | chromosome 2 | OV14_RS25830 | WP_025429489.1 | ABC transporter substrate-binding protein [Ensifer adhaerens] |
|  |  | chromosome 2 | OV14_RS25835 | WP_025429490.1 | MULTISPECIES: LysR family transcriptional regulator [Ensifer] |
|  |  | chromosome 2 | OV14_RS26045 | WP_025429530.1 | MULTISPECIES: MFS transporter [Ensifer] |
|  |  | chromosome 2 | OV14_RS26060 | | Pseudogene |
|  |  | chromosome 2 | OV14_RS26210 | WP_025429564.1 | MFS transporter [Ensifer adhaerens] |
|  |  | chromosome 2 | OV14_RS26245 | WP_051509306.1 | muconate cycloisomerase [Ensifer adhaerens] |
|  |  | chromosome 2 | OV14_RS26255 | WP_051509307.1 | ABC transporter ATP-binding protein [Ensifer adhaerens] |
|  |  | chromosome 2 | OV14_RS26270 | WP_038578537.1 | hypothetical protein [Ensifer adhaerens] |
|  |  | chromosome 2 | OV14_RS26375 | WP_025429597.1 | MFS transporter [Ensifer adhaerens] |
|  |  | chromosome 2 | OV14_RS26380 | WP_025429598.1 | MULTISPECIES: LysR family transcriptional regulator [Ensifer] |
|  |  | chromosome 2 | OV14_RS26575 | WP_025429636.1 | replication initiation protein RepC [Ensifer adhaerens] |
|  |  | chromosome 2 | OV14_RS26585 | WP_025429638.1 | MULTISPECIES: hypothetical protein [Ensifer] |
|  |  | chromosome 2 | OV14_RS26645 | WP_025429650.1 | MULTISPECIES: LamB/YcsF family protein [Ensifer] |
|  |  | chromosome 2 | OV14_RS26780 | WP_025429677.1 | MULTISPECIES: ABC transporter ATP-binding protein [Ensifer] |
|  |  | chromosome 2 | OV14_RS26810 | WP_025429683.1 | MULTISPECIES: LysR family transcriptional regulator [Ensifer] |
|  |  | chromosome 2 | OV14_RS26815 | WP_025429684.1 | hypothetical protein [Ensifer adhaerens] |
|  |  | chromosome 2 | OV14_RS26885 | WP_025429698.1 | hypothetical protein [Ensifer adhaerens] |
|  |  | chromosome 2 | OV14_RS27015 | WP_025429723.1 | selenide, water dikinase [Ensifer adhaerens] |
|  |  | chromosome 2 | OV14_RS27575 | WP_025429829.1 | AsnC family transcriptional regulator [Ensifer adhaerens] |
|  |  | chromosome 2 | OV14_RS27580 | WP_025429830.1 | hypothetical protein [Ensifer adhaerens] |
|  |  | chromosome 2 | OV14_RS27800 | WP_025429875.1 | MULTISPECIES: hydrolase [Ensifer] |
|  |  | chromosome 2 | OV14_RS27815 | WP_025429878.1 | hypothetical protein [Ensifer adhaerens] |
|  |  | chromosome 2 | OV14_RS28010 | WP_064742385.1 | hypothetical protein [Ensifer adhaerens] |
|  |  | chromosome 2 | OV14_RS28015 | WP_051509315.1 | MULTISPECIES: C4-dicarboxylate ABC transporter permease [Ensifer] |
|  |  | chromosome 2 | OV14_RS28100 | WP_025429933.1 | MULTISPECIES: sugar ABC transporter ATP-binding protein [Ensifer] |
|  |  | chromosome 2 | OV14_RS28230 | WP_025429959.1 | MULTISPECIES: alpha-ketoglutarate-dependent dioxygenase AlkB [Ensifer] |
|  |  | chromosome 2 | OV14_RS28250 | WP_025429963.1 | hypothetical protein [Ensifer adhaerens] |
|  |  | plasmid b | OV14_RS28995 | WP_025430094.1 | MULTISPECIES: guanylyl cyclase [Ensifer] |
|  |  | plasmid b | OV14_RS29070 | WP_025430109.1 | MULTISPECIES: sugar ABC transporter permease [Ensifer] |
|  |  | plasmid b | OV14_RS29150 | WP_025430125.1 | membrane protein [Ensifer adhaerens] |
|  |  | plasmid b | OV14_RS29160 | WP_025430127.1 | MULTISPECIES: zinc-binding dehydrogenase [Ensifer] |
|  |  | plasmid b | OV14_RS29165 | WP_025430128.1 | FAD-dependent oxidoreductase [Ensifer adhaerens] |
|  |  | plasmid b | OV14_RS29260 | WP_041692912.1 | MULTISPECIES: polyphosphate kinase 2 [Ensifer] |
|  |  | plasmid b | OV14_RS29425 | WP_041692914.1 | ABC transporter substrate-binding protein [Ensifer adhaerens] |
|  |  | plasmid b | OV14_RS29430 | WP_025430178.1 | MULTISPECIES: chemotaxis protein CheY [Ensifer] |
|  |  | plasmid b | OV14_RS29840 | WP_025430258.1 | MULTISPECIES: GntR family transcriptional regulator [Ensifer] |
|  |  | plasmid b | OV14_RS30080 | WP_041692918.1 | MULTISPECIES: hypothetical protein [Ensifer] |
|  |  | plasmid b | OV14_RS30175 | WP_025430323.1 | MULTISPECIES: 3-oxoacyl-ACP reductase [Ensifer] |
|  |  | plasmid b | OV14_RS30225 | WP_051509370.1 | MULTISPECIES: thiamine permease [Ensifer] |
|  |  | plasmid b | OV14_RS30240 | WP_025430336.1 | MULTISPECIES: LysR family transcriptional regulator [Ensifer] |
|  |  | plasmid b | OV14_RS30345 | WP_063963255.1 | MULTISPECIES: LacI family transcriptional regulator [Ensifer] |
|  |  | plasmid b | OV14_RS30400 | WP_025430368.1 | LysR family transcriptional regulator [Ensifer adhaerens] |
|  |  | plasmid b | OV14_RS30420 | WP_041693041.1 | ABC transporter permease [Ensifer adhaerens] |
|  |  | plasmid b | OV14_RS30455 | WP_041692920.1 | alcohol dehydrogenase [Ensifer adhaerens] |
|  |  | plasmid b | OV14_RS30470 | WP_025430381.1 | hypothetical protein [Ensifer adhaerens] |
|  |  | plasmid b | OV14_RS30475 | WP_025430382.1 | peptide ABC transporter substrate-binding protein [Ensifer adhaerens] |
|  |  | plasmid b | OV14_RS30480 | WP_025430383.1 | ABC transporter permease [Ensifer adhaerens] |
|  |  | plasmid b | OV14_RS30605 | WP_025430408.1 | GntR family transcriptional regulator [Ensifer adhaerens] |
|  |  | plasmid b | OV14_RS30735 | WP_025430433.1 | DNA-binding response regulator [Ensifer adhaerens] |
|  |  | plasmid b | OV14_RS30780 | WP_025430441.1 | sodium-independent anion transporter [Ensifer adhaerens] |
|  |  | plasmid b | OV14_RS31070 | WP_025430497.1 | MULTISPECIES: TetR family transcriptional regulator [Ensifer] |
|  |  | plasmid b | OV14_RS31080 | WP_025430499.1 | MULTISPECIES: hypothetical protein [Ensifer] |
|  |  | plasmid b | OV14_RS31115 | WP_025430505.1 | MULTISPECIES: MFS transporter [Ensifer] |
|  |  | plasmid b | OV14_RS31205 | WP_041692928.1 | hypothetical protein [Ensifer adhaerens] |
|  |  | plasmid b | OV14_RS31260 | WP_051509333.1 | ABC transporter ATP-binding protein [Ensifer adhaerens] |
|  |  | plasmid b | nhaA | WP_025430575.1 | Na+/H+ antiporter NhaA [Ensifer adhaerens] |
|  |  | plasmid b | OV14_RS31470 | WP_025430577.1 | LysR family transcriptional regulator [Ensifer adhaerens] |
|  |  | plasmid b | OV14_RS31590 | WP_025430599.1 | hypothetical protein [Ensifer adhaerens] |
|  |  | plasmid b | OV14_RS31675 | WP_025430615.1 | DNA-binding protein [Ensifer adhaerens] |
|  |  | plasmid b | OV14_RS31680 | WP_025430616.1 | hypothetical protein [Ensifer adhaerens] |
|  |  | plasmid b | OV14_RS31775 | WP_025430634.1 | pyridoxal 4-dehydrogenase [Ensifer adhaerens] |
|  |  | plasmid b | OV14_RS31810 | WP_025430641.1 | dehydrogenase [Ensifer adhaerens] |
|  |  | plasmid b | OV14_RS31880 | WP_025430655.1 | GntR family transcriptional regulator [Ensifer adhaerens] |
|  |  | plasmid b | OV14_RS31940 | WP_025430667.1 | transposase [Ensifer adhaerens] |
|  |  | plasmid b | OV14_RS31960 | WP_025430671.1 | transcriptional regulator [Ensifer adhaerens] |
|  |  | plasmid b | OV14_RS32085 | WP_025430696.1 | sugar ABC transporter substrate-binding protein [Ensifer adhaerens] |
|  |  | plasmid b | OV14_RS32140 | | Pseudogene |
|  |  | plasmid b | OV14_RS32160 | WP_025430709.1 | MULTISPECIES: peptide ABC transporter permease [Ensifer] |
|  |  | plasmid b | OV14_RS32300 | WP_025430735.1 | sorbosone dehydrogenase [Ensifer adhaerens] |
|  |  | plasmid b | OV14_RS32320 | WP_041692940.1 | ATP-dependent DNA ligase [Ensifer adhaerens] |
|  |  | plasmid b | OV14_RS32370 | | Pseudogene |
|  |  | plasmid b | OV14_RS32380 | | hypothetical protein |
|  |  | plasmid b | OV14_RS32425 | WP_025430758.1 | hypothetical protein [Ensifer adhaerens] |
|  |  | plasmid b | OV14_RS32520 | WP_025430778.1 | universal stress protein [Ensifer adhaerens] |
|  |  | plasmid b | OV14_RS32570 | WP_025430787.1 | MULTISPECIES: transcriptional regulator [Ensifer] |
|  |  | plasmid b | OV14_RS32805 | WP_041692950.1 | hypothetical protein [Ensifer adhaerens] |
|  |  | plasmid b | OV14_RS32910 | WP_025430852.1 | ribose ABC transporter substrate-binding protein [Ensifer adhaerens] |
|  |  | plasmid b | OV14_RS32915 | WP_025430853.1 | gluconolactonase [Ensifer adhaerens] |
|  |  | plasmid b | OV14_RS32960 | WP_025430861.1 | ABC transporter substrate-binding protein [Ensifer adhaerens] |
|  |  | plasmid b | OV14_RS32965 | WP_025430862.1 | LacI family transcriptional regulator [Ensifer adhaerens] |
|  |  | plasmid b | OV14_RS32970 | WP_025430863.1 | hypothetical protein [Ensifer adhaerens] |
|  |  | plasmid b | OV14_RS32975 | WP_025430864.1 | AraC family transcriptional regulator [Ensifer adhaerens] |
|  |  | plasmid b | OV14_RS32995 | WP_025430867.1 | hypothetical protein [Ensifer adhaerens] |
|  |  | plasmid b | OV14_RS33010 | WP_025430870.1 | hypothetical protein [Ensifer adhaerens] |
|  |  | plasmid b | OV14_RS33165 | WP_025430899.1 | hypothetical protein [Ensifer adhaerens] |
|  |  | plasmid b | OV14_RS33470 | WP_025430956.1 | transcriptional regulator [Ensifer adhaerens] |
|  |  | plasmid b | OV14_RS33485 | WP_041692956.1 | hypothetical protein [Ensifer adhaerens] |
|  |  | plasmid b | OV14_RS33530 | WP_025430968.1 | ABC transporter permease [Ensifer adhaerens] |
|  |  | plasmid b | OV14_RS33630 | WP_025430988.1 | GntR family transcriptional regulator [Ensifer adhaerens] |
|  |  | plasmid b | OV14_RS34220 | WP_051509349.1 | cytochrome c [Ensifer adhaerens] |
|  |  | plasmid b | OV14_RS34290 | WP_051509350.1 | LacI family transcriptional regulator [Ensifer adhaerens] |
|  |  | plasmid b | OV14_RS34430 | WP_041692967.1 | hypothetical protein [Ensifer adhaerens] |
|  |  | plasmid b | OV14_RS34865 | WP_025431213.1 | hypothetical protein [Ensifer adhaerens] |
|  |  | plasmid b | OV14_RS35020 | WP_025431242.1 | hypothetical protein [Ensifer adhaerens] |
|  |  | plasmid b | OV14_RS35040 | WP_025431246.1 | hypothetical protein [Ensifer adhaerens] |
|  |  | plasmid b | OV14_RS35300 | WP_025431289.1 | replication initiation protein RepC [Ensifer adhaerens] |
|  |  | plasmid b | OV14_RS35580 | WP_025431344.1 | hypothetical protein [Ensifer adhaerens] |
|  |  | plasmid b | OV14_RS35625 | WP_025431353.1 | hypothetical protein [Ensifer adhaerens] |
|  |  | plasmid b | OV14_RS35775 | WP_051509363.1 | GntR family transcriptional regulator [Ensifer adhaerens] |
|  |  | plasmid b | OV14_RS35780 | WP_025431380.1 | MULTISPECIES: ectoine/hydroxyectoine ABC transporter ATP-binding protein EhuA [Ensifer] |
|  |  | plasmid b | OV14_RS35785 | WP_025431381.1 | ectoine/hydroxyectoine ABC transporter substrate-binding protein EhuB [Ensifer adhaerens] |
|  |  | plasmid b | OV14_RS35790 | WP_025431382.1 | ectoine/hydroxyectoine ABC transporter permease subunit EhuC [Ensifer adhaerens] |
|  |  | plasmid b | OV14_RS35875 | WP_025431398.1 | MULTISPECIES: alkylhydroperoxidase [Ensifer] |
|  |  | plasmid b | OV14_RS36095 | WP_025431439.1 | NADH-quinone oxidoreductase subunit I [Ensifer adhaerens] |
|  |  | plasmid b | OV14_RS36115 | WP_025431443.1 | NADH dehydrogenase (quinone) subunit G [Ensifer adhaerens] |
|  |  | plasmid b | OV14_RS36120 | WP_025431444.1 | NADH oxidoreductase (quinone) subunit F [Ensifer adhaerens] |
|  |  | plasmid b | OV14_RS36140 | WP_025431448.1 | MULTISPECIES: NADH dehydrogenase [Ensifer] |
|  |  | plasmid b | OV14_RS36145 | WP_025431449.1 | MULTISPECIES: NADH-quinone oxidoreductase subunit A [Ensifer] |
|  |  | plasmid c | OV14_RS28330 | WP_025429975.1 | hypothetical protein [Ensifer adhaerens] |
|  |  | plasmid c | OV14_RS28440 | WP_038578847.1 | hypothetical protein [Ensifer adhaerens] |
|  |  | plasmid 5105 | virK | ABK01290.1 | VirK protein; pfam06903 |
| D5(BR) D7(BR) | 112 | chromosome 1 | OV14_RS00240 | WP_025424581.1 | hypothetical protein [Ensifer adhaerens] |
|  |  | chromosome 1 | OV14_RS01340 | WP_025424791.1 | MULTISPECIES: hypothetical protein [Ensifer] |
|  |  | chromosome 1 | OV14_RS01525 | WP_025424827.1 | MULTISPECIES: adenylate cyclase [Ensifer] |
|  |  | chromosome 1 | OV14_RS01695 | WP_025424859.1 | MULTISPECIES: hypothetical protein [Ensifer] |
|  |  | chromosome 1 | OV14_RS02285 | WP_025424973.1 | MULTISPECIES: iron ABC transporter ATP-binding protein [Ensifer] |
|  |  | chromosome 1 | OV14_RS02435 | WP_025424994.1 | MULTISPECIES: hypothetical protein [Ensifer] |
|  |  | chromosome 1 | OV14_RS02440 | WP_025424995.1 | MULTISPECIES: hypothetical protein [Ensifer] |
|  |  | chromosome 1 | OV14_RS02450 | WP_025424997.1 | MULTISPECIES: hypothetical protein [Ensifer] |
|  |  | chromosome 1 | OV14_RS02635 | WP_025425033.1 | MULTISPECIES: formate dehydrogenase accessory protein FdhD [Ensifer] |
|  |  | chromosome 1 | OV14_RS04250 | WP_025425347.1 | MULTISPECIES: phosphoenolpyruvate carboxykinase (ATP) [Ensifer] |
|  |  | chromosome 1 | OV14_RS04480 | WP_025425386.1 | MULTISPECIES: pilus assembly protein [Ensifer] |
|  |  | chromosome 1 | OV14_RS05050 | WP_025425498.1 | MULTISPECIES: hypothetical protein [Ensifer] |
|  |  | chromosome 1 | OV14_RS05270 | WP_025425539.1 | MULTISPECIES: MFS transporter [Ensifer] |
|  |  | chromosome 1 | OV14_RS05305 | WP_025425545.1 | MULTISPECIES: membrane protein [Ensifer] |
|  |  | chromosome 1 | OV14_RS05875 | WP_025425655.1 | MULTISPECIES: hypothetical protein [Ensifer] |
|  |  | chromosome 1 | OV14_RS06125 | WP_025425705.1 | MULTISPECIES: alanine acetyltransferase [Ensifer] |
|  |  | chromosome 1 | OV14_RS06400 | WP_025425759.1 | MULTISPECIES: hypothetical protein [Ensifer] |
|  |  | chromosome 1 | OV14_RS07180 | WP_025425909.1 | MULTISPECIES: flagellar biosynthesis protein FlhB [Ensifer] |
|  |  | chromosome 1 | OV14_RS07530 | WP_025425977.1 | MULTISPECIES: hypothetical protein [Ensifer] |
|  |  | chromosome 1 | OV14_RS07590 | WP_025425989.1 | MULTISPECIES: phasin [Ensifer] |
|  |  | chromosome 1 | OV14_RS07685 | WP_025426007.1 | MULTISPECIES: AI-2E family transporter [Ensifer] |
|  |  | chromosome 1 | OV14_RS36345 | WP_025426205.1 | MULTISPECIES: hypothetical protein [Ensifer] |
|  |  | chromosome 1 | OV14_RS08790 | WP_025426221.1 | MULTISPECIES: sodium transporter [Ensifer] |
|  |  | chromosome 1 | OV14_RS08925 | WP_025426248.1 | MULTISPECIES: dihydrofolate reductase [Ensifer] |
|  |  | chromosome 1 | OV14_RS09380 | WP_025426338.1 | MULTISPECIES: hypothetical protein [Ensifer] |
|  |  | chromosome 1 | OV14_RS10535 | WP_025426560.1 | MULTISPECIES: membrane protein [Ensifer] |
|  |  | chromosome 1 | OV14_RS11605 | WP_025426757.1 | MULTISPECIES: CDP-diacylglycerol--glycerol-3-phosphate 3-phosphatidyltransferase [Ensifer] |
|  |  | chromosome 1 | OV14_RS11825 | WP_025426800.1 | MULTISPECIES: amino acid ABC transporter permease [Ensifer] |
|  |  | chromosome 1 | OV14_RS36815 | WP_065781539.1 | MULTISPECIES: DUF1127 domain-containing protein [Ensifer] |
|  |  | chromosome 1 | OV14_RS12455 | WP_025426921.1 | hypothetical protein [Ensifer adhaerens] |
|  |  | chromosome 1 | OV14_RS12545 | WP_025426937.1 | hypothetical protein [Ensifer adhaerens] |
|  |  | chromosome 1 | OV14_RS12560 | WP_025426940.1 | hypothetical protein [Ensifer adhaerens] |
|  |  | chromosome 1 | OV14_RS12570 | WP_025426942.1 | hypothetical protein [Ensifer adhaerens] |
|  |  | chromosome 1 | OV14_RS12580 | WP_025426944.1 | hypothetical protein [Ensifer adhaerens] |
|  |  | chromosome 1 | OV14_RS12605 | WP_025426949.1 | hypothetical protein [Ensifer adhaerens] |
|  |  | chromosome 1 | OV14_RS12695 | WP_025426966.1 | hypothetical protein [Ensifer adhaerens] |
|  |  | chromosome 1 | OV14_RS12750 | WP_025426975.1 | hypothetical protein [Ensifer adhaerens] |
|  |  | chromosome 1 | OV14_RS13170 | WP_025427051.1 | MULTISPECIES: hypothetical protein [Ensifer] |
|  |  | chromosome 1 | OV14_RS13195 | WP_025427055.1 | MULTISPECIES: membrane protein [Ensifer] |
|  |  | chromosome 1 | OV14_RS13340 | WP_025427080.1 | MULTISPECIES: MFS transporter [Ensifer] |
|  |  | chromosome 1 | OV14_RS13720 | WP_038576122.1 | MULTISPECIES: hypothetical protein [Ensifer] |
|  |  | chromosome 1 | OV14_RS13970 | WP_025427204.1 | MULTISPECIES: hypothetical protein [Ensifer] |
|  |  | chromosome 1 | OV14_RS14215 | WP_025427253.1 | MULTISPECIES: chemotaxis protein [Ensifer] |
|  |  | chromosome 1 | OV14_RS14770 | WP_025427363.1 | MULTISPECIES: transpeptidase [Ensifer] |
|  |  | chromosome 1 | OV14_RS14795 | WP_025427368.1 | MULTISPECIES: cobalamin biosynthesis protein CobE [Ensifer] |
|  |  | chromosome 1 | OV14_RS15435 | WP_025427497.1 | MULTISPECIES: hypothetical protein [Ensifer] |
|  |  | chromosome 1 | OV14_RS16285 | WP_025427661.1 | calcium/proton exchanger [Ensifer adhaerens] |
|  |  | chromosome 1 | OV14_RS36435 | WP_025427680.1 | MULTISPECIES: GGDEF domain-containing protein [Ensifer] |
|  |  | chromosome 1 | OV14_RS16455 | WP_025427694.1 | MULTISPECIES: GGDEF domain-containing protein [Ensifer] |
|  |  | chromosome 1 | OV14_RS17265 | WP_025427851.1 | mechanosensitive ion channel protein [Ensifer adhaerens] |
|  |  | chromosome 1 | OV14_RS17430 | WP_025427880.1 | MULTISPECIES: hypothetical protein [Ensifer] |
|  |  | chromosome 1 | OV14_RS17435 | WP_025427881.1 | MULTISPECIES: GYD family protein [Ensifer] |
|  |  | chromosome 1 | OV14_RS17580 | WP_025427908.1 | MULTISPECIES: hypothetical protein [Ensifer] |
|  |  | chromosome 1 | OV14_RS17860 | WP_025427963.1 | MULTISPECIES: cold-shock protein [Rhizobiaceae] |
|  |  | chromosome 1 | OV14_RS18360 | WP_025428061.1 | MULTISPECIES: short-chain dehydrogenase/reductase [Ensifer] |
|  |  | chromosome 1 | OV14_RS18530 | WP_025428093.1 | MULTISPECIES: hypothetical protein [Ensifer] |
|  |  | chromosome 1 | OV14_RS18605 | WP_025428108.1 | dihydropyrimidine dehydrogenase [Ensifer adhaerens] |
|  |  | chromosome 1 | OV14_RS18640 | WP_025428115.1 | MULTISPECIES: TetR family transcriptional regulator [Ensifer] |
|  |  | chromosome 1 | OV14_RS18775 | WP_025428142.1 | MULTISPECIES: branched chain amino acid ABC transporter substrate-binding protein [Ensifer] |
|  |  | chromosome 1 | OV14_RS18795 | WP_025428146.1 | MULTISPECIES: branched-chain amino acid ABC transporter permease [Ensifer] |
|  |  | chromosome 1 | OV14_RS18905 | WP_025428168.1 | MULTISPECIES: amino acid ABC transporter substrate-binding protein [Ensifer] |
|  |  | chromosome 1 | OV14_RS18910 | WP_025428169.1 | MULTISPECIES: agmatinase [Ensifer] |
|  |  | chromosome 2 | OV14_RS19385 | | Hypothetical protein |
|  |  | chromosome 2 | OV14_RS19515 | WP_025428281.1 | glutamine amidotransferase [Ensifer adhaerens] |
|  |  | chromosome 2 | OV14_RS19520 | WP_025428282.1 | enterochelin esterase [Ensifer adhaerens] |
|  |  | chromosome 2 | OV14_RS19815 | WP_025428340.1 | MULTISPECIES: LuxR family transcriptional regulator [Ensifer] |
|  |  | chromosome 2 | OV14_RS19820 | WP_051509272.1 | MULTISPECIES: Crp/Fnr family transcriptional regulator [Ensifer] |
|  |  | chromosome 2 | OV14_RS19900 | WP_025428357.1 | MULTISPECIES: LPS biosynthesis protein [Ensifer] |
|  |  | chromosome 2 | OV14_RS20030 | WP_025428379.1 | phosphoglycerate mutase [Ensifer adhaerens] |
|  |  | chromosome 2 | OV14_RS20190 | WP_025428408.1 | MULTISPECIES: transcriptional regulator [Ensifer] |
|  |  | chromosome 2 | OV14_RS20295 | WP_025428429.1 | MULTISPECIES: membrane protein [Ensifer] |
|  |  | chromosome 2 | OV14_RS21505 | WP_025428664.1 | MULTISPECIES: urocanate hydratase [Ensifer] |
|  |  | chromosome 2 | OV14_RS22410 | WP_025428840.1 | amino acid ABC transporter substrate-binding protein [Ensifer adhaerens] |
|  |  | chromosome 2 | OV14_RS22595 | WP_025428877.1 | C4-dicarboxylate ABC transporter [Ensifer adhaerens] |
|  |  | chromosome 2 | OV14_RS23035 | | Pseudogene |
|  |  | chromosome 2 | OV14_RS24095 | WP_025429158.1 | adenylate/guanylate cyclase domain-containing protein [Ensifer adhaerens] |
|  |  | chromosome 2 | OV14_RS24100 | WP_025429159.1 | MULTISPECIES: branched chain amino acid ABC transporter substrate-binding protein [Ensifer] |
|  |  | chromosome 2 | OV14_RS24710 | WP_025429280.1 | MULTISPECIES: LysR family transcriptional regulator [Ensifer] |
|  |  | chromosome 2 | OV14_RS25450 | WP_051509301.1 | MULTISPECIES: MFS transporter [Ensifer] |
|  |  | chromosome 2 | OV14_RS25790 | WP_025429481.1 | hypothetical protein [Ensifer adhaerens] |
|  |  | chromosome 2 | OV14_RS26075 | WP_025429538.1 | aminotransferase class V [Ensifer adhaerens] |
|  |  | chromosome 2 | OV14_RS27060 | WP_025429731.1 | histidine kinase [Ensifer adhaerens] |
|  |  | chromosome 2 | OV14_RS27370 | WP_025429790.1 | MULTISPECIES: ABC transporter ATP-binding protein [Ensifer] |
|  |  | chromosome 2 | OV14_RS27380 | WP_025429792.1 | MULTISPECIES: hypothetical protein [Ensifer] |
|  |  | chromosome 2 | OV14_RS27610 | WP_025429836.1 | MULTISPECIES: hypothetical protein [Ensifer] |
|  |  | chromosome 2 | OV14_RS28155 | WP_038578733.1 | ABC transporter substrate-binding protein [Ensifer adhaerens] |
|  |  | plasmid b | OV14_RS28975 | WP_025430090.1 | MULTISPECIES: hypothetical protein [Ensifer] |
|  |  | plasmid b | OV14_RS29145 | WP_025430124.1 | MULTISPECIES: hypothetical protein [Ensifer] |
|  |  | plasmid b | OV14_RS29495 | WP_025430191.1 | short-chain dehydrogenase [Ensifer adhaerens] |
|  |  | plasmid b | OV14_RS30615 | WP_025430410.1 | MULTISPECIES: peptide ABC transporter permease [Ensifer] |
|  |  | plasmid b | OV14_RS30775 | WP_041692922.1 | hypothetical protein [Ensifer adhaerens] |
|  |  | plasmid b | OV14_RS30815 | WP_025430448.1 | hypothetical protein [Ensifer adhaerens] |
|  |  | plasmid b | OV14_RS31210 | WP_025430524.1 | hypothetical protein [Ensifer adhaerens] |
|  |  | plasmid b | OV14_RS31430 | WP_025430569.1 | addiction module antitoxin [Ensifer adhaerens] |
|  |  | plasmid b | OV14_RS32185 | WP_025430714.1 | amino acid dehydrogenase [Ensifer adhaerens] |
|  |  | plasmid b | OV14_RS32205 | WP_025430718.1 | hypothetical protein [Ensifer adhaerens] |
|  |  | plasmid b | OV14_RS32245 | WP_025430726.1 | hypothetical protein [Ensifer adhaerens] |
|  |  | plasmid b | OV14_RS32345 | WP_025430744.1 | hypothetical protein [Ensifer adhaerens] |
|  |  | plasmid b | OV14_RS32550 | WP_025430783.1 | hypothetical protein [Ensifer adhaerens] |
|  |  | plasmid b | OV14_RS32790 | WP_025430828.1 | hypothetical protein [Ensifer adhaerens] |
|  |  | plasmid b | OV14_RS32835 | WP_025430837.1 | molecular chaperone GroEL [Ensifer adhaerens] |
|  |  | plasmid b | OV14_RS33080 | WP_025430884.1 | MULTISPECIES: spermidine/putrescine ABC transporter permease [Ensifer] |
|  |  | plasmid b | OV14_RS33170 | | Pseudogene |
|  |  | plasmid b | OV14_RS33730 | WP_025431004.1 | MULTISPECIES: tautomerase [Ensifer] |
|  |  | plasmid b | OV14_RS33925 | WP_025431041.1 | hypothetical protein [Ensifer adhaerens] |
|  |  | plasmid b | OV14_RS34650 | WP_025431171.1 | hypothetical protein [Ensifer adhaerens] |
|  |  | plasmid b | OV14_RS35115 | WP_025431259.1 | hypothetical protein [Ensifer adhaerens] |
|  |  | plasmid b | OV14_RS35160 | | Pseudogene |
|  |  | plasmid b | OV14_RS36060 | WP_064742398.1 | hypothetical protein [Ensifer adhaerens] |
|  |  | plasmid b | OV14_RS36110 | WP_025431442.1 | MULTISPECIES: IS110 family transposase [Ensifer] |
|  |  | plasmid b | OV14_RS36125 | WP_025431445.1 | NADH-quinone oxidoreductase subunit E [Ensifer adhaerens] |
|  |  | plasmid b | OV14_RS36130 | WP_025431446.1 | NADH dehydrogenase (quinone) subunit D [Ensifer adhaerens] |
| D3(BR) D7(BR) | 15 | chromosome 1 | OV14_RS00305 | WP_025424593.1 | WP_025424593.1 hypothetical protein [Ensifer adhaerens] |
|  |  | chromosome 1 | OV14_RS00885 | WP_003527546.1 | WP_003527546.1 MULTISPECIES: hypothetical protein [Sinorhizobium/Ensifer group] |
|  |  | chromosome 1 | OV14_RS07120 | WP_025425897.1 | WP_025425897.1 MULTISPECIES: Fis family transcriptional regulator [Sinorhizobium/Ensifer group] |
|  |  | chromosome 1 | OV14_RS08160 | WP_025426099.1 | WP_025426099.1 MULTISPECIES: F0F1 ATP synthase subunit A [Ensifer] |
|  |  | chromosome 1 | OV14_RS08935 | WP_025426250.1 | WP_025426250.1 MULTISPECIES: LysR family transcriptional regulator [Ensifer] |
|  |  | chromosome 1 | OV14_RS11830 | WP_025426801.1 | WP_025426801.1 MULTISPECIES: amino acid ABC transporter permease [Ensifer] |
|  |  | chromosome 1 | OV14_RS12770 | WP_025426979.1 | WP_025426979.1 hypothetical protein [Ensifer adhaerens] |
|  |  | chromosome 1 | OV14_RS15645 | WP_025427534.1 | WP_025427534.1 MULTISPECIES: endoribonuclease [Ensifer] |
|  |  | chromosome 1 | OV14_RS15805 | WP_038577200.1 | WP_038577200.1 MULTISPECIES: carnitine operon oxidoreductase [Ensifer] |
|  |  | chromosome 1 | OV14_RS18465 | WP_025428081.1 | WP_025428081.1 MULTISPECIES: TetR family transcriptional regulator [Ensifer] |
|  |  | chromosome 2 | OV14_RS27135 | WP_063978400.1 | WP_063978400.1 MULTISPECIES: hypothetical protein [Ensifer] |
|  |  | plasmid b | OV14_RS31265 | WP_025430535.1 | WP_025430535.1 UDP-glucose 4-epimerase [Ensifer adhaerens] |
|  |  | plasmid b | OV14_RS32290 | WP_025430733.1 | WP_025430733.1 hypothetical protein [Ensifer adhaerens] |
|  |  | plasmid b | OV14_RS33140 | WP_025430894.1 | WP_025430894.1 histidinol dehydrogenase [Ensifer adhaerens] |
|  |  | plasmid b | OV14_RS35905 | WP_025431404.1 | WP_025431404.1 spermidine/putrescine ABC transporter substrate-binding protein [Ensifer adhaerens] |
| D3(BR) D5(BR) | 26 | chromosome 1 | OV14_RS01320 | WP_025424787.1 | WP_025424787.1 MULTISPECIES: 50S ribosomal protein L36 [Ensifer] |
|  |  | chromosome 1 | OV14_RS02030 | WP_025424924.1 | WP_025424924.1 MULTISPECIES: hypothetical protein [Ensifer] |
|  |  | chromosome 1 | OV14_RS02200 | WP_025424956.1 | WP_025424956.1 MULTISPECIES: 4-hydroxyphenylpyruvate dioxygenase [Ensifer] |
|  |  | chromosome 1 | OV14_RS07710 | WP_025426012.1 | WP_025426012.1 MULTISPECIES: CsbD family protein [Ensifer] |
|  |  | chromosome 1 | OV14_RS08165 | WP_025426100.1 | WP_025426100.1 MULTISPECIES: ATP F0F1 synthase subunit C [Sinorhizobium/Ensifer group] |
|  |  | chromosome 1 | OV14_RS10270 | WP_025426509.1 | WP_025426509.1 MULTISPECIES: cytochrome P450 [Ensifer] |
|  |  | chromosome 1 | OV14_RS12340 | WP_025426898.1 | WP_025426898.1 integrase [Ensifer adhaerens] |
|  |  | chromosome 1 | OV14_RS12365 | WP_025426903.1 | WP_025426903.1 hypothetical protein [Ensifer adhaerens] |
|  |  | chromosome 1 | OV14_RS12645 | WP_025426957.1 | WP_025426957.1 hypothetical protein [Ensifer adhaerens] |
|  |  | chromosome 1 | OV14_RS17620 | WP_025427916.1 | WP_025427916.1 MULTISPECIES: tRNA-binding protein [Ensifer] |
|  |  | chromosome 2 | OV14_RS36460 | WP_063963266.1 | WP_063963266.1 MULTISPECIES: hypothetical protein [Ensifer] |
|  |  | chromosome 2 | OV14_RS21715 | WP_025428706.1 | WP_025428706.1 MULTISPECIES: hypothetical protein [Ensifer] |
|  |  | chromosome 2 | OV14_RS22675 | WP_025428892.1 | WP_025428892.1 hypothetical protein [Ensifer adhaerens] |
|  |  | chromosome 2 | OV14_RS23200 | WP_025428983.1 | WP_025428983.1 transposase [Ensifer adhaerens] |
|  |  | chromosome 2 | OV14_RS24040 | WP_025429147.1 | WP_025429147.1 X-Pro dipeptidase [Ensifer adhaerens] |
|  |  | chromosome 2 | OV14_RS25905 | WP_025429503.1 | WP_025429503.1 hypothetical protein [Ensifer adhaerens] |
|  |  | chromosome 2 | OV14_RS25940 | WP_025429510.1 | WP_025429510.1 methyltransferase [Ensifer adhaerens] |
|  |  | chromosome 2 | OV14_RS27035 | WP_051509310.1 | WP_051509310.1 MULTISPECIES: hypothetical protein [Ensifer] |
|  |  | chromosome 2 | OV14_RS27040 | WP_025429728.1 | WP_025429728.1 MULTISPECIES: N-formylglutamate amidohydrolase [Ensifer] |
|  |  | plasmid b | OV14_RS30820 | WP_025430449.1 | WP_025430449.1 exodeoxyribonuclease III [Ensifer adhaerens] |
|  |  | plasmid b | OV14_RS32195 | WP_025430716.1 | WP_025430716.1 hypothetical protein [Ensifer adhaerens] |
|  |  | plasmid b | OV14_RS32200 | WP_025430717.1 | WP_025430717.1 hypothetical protein [Ensifer adhaerens] |
|  |  | plasmid b | OV14_RS32270 | WP_025430730.1 | WP_025430730.1 hypothetical protein [Ensifer adhaerens] |
|  |  | plasmid b | OV14_RS32845 | WP_025430839.1 | WP_025430839.1 epimerase [Ensifer adhaerens] |
|  |  | plasmid b | OV14_RS33175 | WP_025430900.1 | WP_025430900.1 hypothetical protein [Ensifer adhaerens] |
|  |  | plasmid c | OV14_RS28855 | WP_025430075.1 | WP_025430075.1 hypothetical protein [Ensifer adhaerens] |
| D2(BR) D7(BR) | 1 | chromosome 2 | OV14_RS24680 | WP_025429274.1 | WP_025429274.1 MULTISPECIES: sugar ABC transporter substrate-binding protein [Ensifer] |
| D2(BR) D3(BR) | 34 | chromosome 1 | OV14_RS01375 | WP_025424798.1 | WP_025424798.1sigma-54-dependent Fis family transcriptional regulator [Ensifer adhaerens] |
|  |  | chromosome 1 | OV14_RS02140 | WP_025424944.1 | WP_025424944.1 MULTISPECIES: 3-methyl-2-oxobutanoate dehydrogenase (2-methylpropanoyl-transferring) subunit alpha [Ensifer] |
|  |  | chromosome 1 | OV14_RS02145 | WP_025424945.1 | WP_025424945.1 MULTISPECIES: 2-oxoisovalerate dehydrogenase [Ensifer] |
|  |  | chromosome 1 | OV14_RS02155 | WP_025424947.1 | WP_025424947.1 MULTISPECIES: dihydrolipoyl dehydrogenase [Ensifer] |
|  |  | chromosome 1 | OV14_RS02340 | WP_025424978.1 | WP_025424978.1 MULTISPECIES: ABC transporter permease [Ensifer] |
|  |  | chromosome 1 | OV14_RS02350 | WP_025424980.1 | WP_025424980.1 MULTISPECIES: ABC transporter ATP-binding protein [Ensifer] |
|  |  | chromosome 1 | OV14_RS07110 | WP_025425895.1 | WP_025425895.1 MULTISPECIES: chemotaxis protein [Ensifer] |
|  |  | chromosome 1 | OV14_RS07125 | WP_025425898.1 | WP_025425898.1 MULTISPECIES: chemotaxis protein CheA [Ensifer] |
|  |  | chromosome 1 | OV14_RS07190 | WP_025425911.1 | WP_025425911.1 MULTISPECIES: flagellar motor switch protein FliN [Ensifer] |
|  |  | chromosome 1 | OV14_RS07240 | WP_025425921.1 | WP_025425921.1 MULTISPECIES: flagellar basal body rod protein FlgG [Ensifer] |
|  |  | chromosome 1 | OV14_RS07300 | 26221974 | Pseudogene |
|  |  | chromosome 1 | motB | WP_025425934.1 | WP_025425934.1 MULTISPECIES: flagellar motor protein MotB [Ensifer] |
|  |  | chromosome 1 | OV14_RS07335 | WP_025425938.1 | WP_025425938.1 MULTISPECIES: transcriptional regulator [Ensifer] |
|  |  | chromosome 1 | OV14_RS07340 | WP_025425939.1 | WP_025425939.1 MULTISPECIES: flagellar hook protein FlgE [Ensifer] |
|  |  | chromosome 1 | OV14_RS07350 | WP_025425941.1 | WP_025425941.1 MULTISPECIES: flagellar hook protein FlgL [Ensifer] |
|  |  | chromosome 1 | OV14_RS07390 | WP_025425949.1 | WP_025425949.1 MULTISPECIES: rod-binding protein [Ensifer] |
|  |  | chromosome 1 | OV14_RS07620 | WP_025425994.1 | WP_025425994.1 MULTISPECIES: methylmalonate-semialdehyde dehydrogenase (acylating) [Ensifer] |
|  |  | chromosome 1 | OV14_RS09140 | WP_025426290.1 | WP_025426290.1 MULTISPECIES: glucose dehydrogenase [Ensifer] |
|  |  | chromosome 1 | OV14_RS11415 | WP_025426719.1 | WP_025426719.1 MULTISPECIES: hypothetical protein [Ensifer] |
|  |  | chromosome 1 | OV14_RS17460 | WP_025427886.1 | WP_025427886.1 glycine C-acetyltransferase [Ensifer adhaerens] |
|  |  | chromosome 1 | OV14_RS18450 | WP_025428078.1 | WP_025428078.1 oxidoreductase [Ensifer adhaerens] |
|  |  | chromosome 2 | OV14_RS20210 | WP_025428412.1 | WP_025428412.1 glutamine synthetase [Ensifer adhaerens] |
|  |  | chromosome 2 | OV14_RS21185 | WP_025428602.1 | WP_025428602.1 MULTISPECIES: glyoxylate carboligase [Ensifer] |
|  |  | chromosome 2 | OV14_RS21190 | WP_025428603.1 | WP_025428603.1 hydroxypyruvate isomerase [Ensifer adhaerens] |
|  |  | chromosome 2 | OV14_RS21195 | WP_025428604.1 | WP_025428604.1 MULTISPECIES: 2-hydroxy-3-oxopropionate reductase [Ensifer] |
|  |  | chromosome 2 | OV14_RS21200 | WP_025428605.1 | WP_025428605.1 MULTISPECIES: hydroxypyruvate reductase [Ensifer] |
|  |  | chromosome 2 | OV14_RS23480 | WP_064742371.1 | WP_064742371.1 NAD(P)-dependent oxidoreductase [Ensifer adhaerens] |
|  |  | chromosome 2 | OV14_RS23505 | WP_025429041.1 | WP_025429041.1 hypothetical protein [Ensifer adhaerens] |
|  |  | chromosome 2 | OV14_RS25235 | WP_025429379.1 | WP_025429379.1 transketolase [Ensifer adhaerens] |
|  |  | chromosome 2 | OV14_RS27920 | WP_025429897.1 | WP_025429897.1 MULTISPECIES: hypothetical protein [Ensifer] |
|  |  | chromosome 2 | OV14_RS27925 | WP_025429898.1 | WP_025429898.1 MULTISPECIES: membrane protein [Ensifer] |
|  |  | chromosome 2 | OV14_RS27930 | WP_025429899.1 | WP_025429899.1 MULTISPECIES: hypothetical protein [Ensifer] |
|  |  | plasmid b | OV14_RS29990 | WP_025430288.1 | WP_025430288.1 ABC transporter substrate-binding protein [Ensifer adhaerens] |
|  |  | plasmid b | OV14_RS35715 | WP_025431368.1 | WP_025431368.1 MULTISPECIES: hypothetical protein [Ensifer] |
| D1(BR) D7(BR) | 4 | chromosome 1 | OV14_RS15040 | WP_025427417.1 | WP_025427417.1 MULTISPECIES: hypothetical protein [Ensifer] |
|  |  | chromosome 1 | OV14_RS15215 | WP_025427453.1 | WP_025427453.1 MULTISPECIES: transcriptional regulator [Ensifer] |
|  |  | chromosome 2 | OV14_RS23945 | WP_025429128.1 | WP_025429128.1 flavin oxidoreductase [Ensifer adhaerens] |
|  |  | plasmid b | OV14_RS34190 | WP_051509348.1 | WP_051509348.1 5,10-methylenetetrahydromethanopterin reductase [Ensifer adhaerens] |
| D1(BR) D3(BR) | 1 | chromosome 1 | OV14_RS10505 | WP_025426554.1 | WP_025426554.1 MULTISPECIES: phenylalanine 4-monooxygenase [Ensifer] |
| D3(BR) D5(BR) D7(BR) | 75 | chromosome 1 | OV14_RS00190 | WP_025424572.1 | WP_025424572.1 secretion activator protein [Ensifer adhaerens] |
|  |  | chromosome 1 | OV14_RS00205 | WP_025424575.1 | WP_025424575.1 hypothetical protein [Ensifer adhaerens] |
|  |  | chromosome 1 | OV14_RS36285 | WP_051509123.1 | WP_051509123.1 phage tail tape measure protein [Ensifer adhaerens] |
|  |  | chromosome 1 | OV14_RS00235 | WP_038576395.1 | WP_038576395.1 hypothetical protein, partial [Ensifer adhaerens] |
|  |  | chromosome 1 | OV14_RS00245 | WP_038575641.1 | WP_038575641.1 histidine kinase [Ensifer adhaerens] |
|  |  | chromosome 1 | OV14_RS36290 | WP_025424585.1 | WP_025424585.1 hypothetical protein [Ensifer adhaerens] |
|  |  | chromosome 1 | OV14_RS00270 | WP_025424586.1 | WP_025424586.1 hypothetical protein [Ensifer adhaerens] |
|  |  | chromosome 1 | OV14_RS00275 | WP_025424587.1 | WP_025424587.1 hypothetical protein [Ensifer adhaerens] |
|  |  | chromosome 1 | OV14_RS00285 | WP_025424589.1 | WP_025424589.1 peptidase [Ensifer adhaerens] |
|  |  | chromosome 1 | OV14_RS00290 | WP_025424590.1 | WP_025424590.1 phage portal protein [Ensifer adhaerens] |
|  |  | chromosome 1 | OV14_RS01030 | WP_025424732.1 | WP_025424732.1 MULTISPECIES: hypothetical protein [Ensifer] |
|  |  | chromosome 1 | OV14_RS02300 | WP_025424976.1 | WP_025424976.1 MULTISPECIES: TetR family transcriptional regulator [Ensifer] |
|  |  | chromosome 1 | OV14_RS02455 | WP_025424998.1 | WP_025424998.1 MULTISPECIES: cold-shock protein [Ensifer] |
|  |  | chromosome 1 | OV14_RS02490 | WP_038576546.1 | WP_038576546.1 MULTISPECIES: hypothetical protein [Ensifer] |
|  |  | chromosome 1 | OV14_RS02625 | WP_025425031.1 | WP_025425031.1 MULTISPECIES: chemotaxis protein [Ensifer] |
|  |  | chromosome 1 | OV14_RS03730 | WP_025425244.1 | WP_025425244.1 MULTISPECIES: LysR family transcriptional regulator [Ensifer] |
|  |  | chromosome 1 | OV14_RS04910 | WP_025425471.1 | WP_025425471.1 MULTISPECIES: transporter [Ensifer] |
|  |  | chromosome 1 | OV14_RS07740 | WP_025426018.1 | WP_025426018.1 MULTISPECIES: hypothetical protein [Ensifer] |
|  |  | chromosome 1 | OV14_RS07775 | WP_025426024.1 | WP_025426024.1 MULTISPECIES: 23S rRNA methyltransferase [Ensifer] |
|  |  | chromosome 1 | OV14_RS08075 | WP_025426082.1 | WP_025426082.1 MULTISPECIES: N-methylproline demethylase [Ensifer] |
|  |  | chromosome 1 | OV14_RS08080 | WP_025426083.1 | WP_025426083.1 MULTISPECIES: photosystem reaction center subunit H [Ensifer] |
|  |  | chromosome 1 | OV14_RS08155 | WP_025426098.1 | WP_025426098.1 MULTISPECIES: ATP synthase subunit I [Ensifer] |
|  |  | chromosome 1 | OV14_RS08355 | WP_038575938.1 | WP_038575938.1 MULTISPECIES: hypothetical protein [Ensifer] |
|  |  | chromosome 1 | OV14_RS08360 | WP_025426139.1 | WP_025426139.1 MULTISPECIES: transporter [Ensifer] |
|  |  | chromosome 1 | OV14_RS08900 | WP_025426243.1 | WP_025426243.1 MULTISPECIES: hypothetical protein [Ensifer] |
|  |  | chromosome 1 | OV14_RS09590 | WP_025426377.1 | WP_025426377.1 MULTISPECIES: hydrolase [Ensifer] |
|  |  | chromosome 1 | OV14_RS36810 | WP_067698885.1 | WP_067698885.1 DUF3563 domain-containing protein [Ensifer adhaerens] |
|  |  | chromosome 1 | OV14_RS09670 | WP_025426393.1 | WP_025426393.1 MULTISPECIES: hypothetical protein [Ensifer] |
|  |  | chromosome 1 | OV14_RS11580 | WP_025426752.1 | WP_025426752.1 MULTISPECIES: acetyltransferase [Ensifer] |
|  |  | chromosome 1 | OV14_RS11640 | WP_025426764.1 | WP_025426764.1 MULTISPECIES: cell division protein [Ensifer] |
|  |  | chromosome 1 | OV14_RS12335 | WP_025426897.1 | WP_025426897.1 MULTISPECIES: hypothetical protein [Ensifer] |
|  |  | chromosome 1 | OV14_RS12565 | WP_025426941.1 | WP_025426941.1 hypothetical protein [Ensifer adhaerens] |
|  |  | chromosome 1 | OV14_RS12685 | WP_025426964.1 | WP_025426964.1 hypothetical protein [Ensifer adhaerens] |
|  |  | chromosome 1 | OV14_RS15425 | WP_025427495.1 | WP_025427495.1 MULTISPECIES: hypothetical protein [Ensifer] |
|  |  | chromosome 1 | OV14_RS16230 | WP_025427650.1 | WP_025427650.1 MULTISPECIES: lysine transporter LysE [Ensifer] |
|  |  | chromosome 1 | OV14_RS16270 | WP_025427658.1 | WP_025427658.1 arylsulfatase [Ensifer adhaerens] |
|  |  | chromosome 1 | OV14_RS17750 | WP_025427942.1 | WP_025427942.1 MULTISPECIES: hypothetical protein [Ensifer] |
|  |  | chromosome 1 | OV14_RS18800 | WP_025428147.1 | WP_025428147.1 branched-chain amino acid ABC transporter permease [Ensifer adhaerens] |
|  |  | chromosome 2 | OV14_RS20660 | WP_025428497.1 | WP_025428497.1 hypothetical protein [Ensifer adhaerens] |
|  |  | chromosome 2 | OV14_RS21375 | WP_025428638.1 | WP_025428638.1 MULTISPECIES: amidinotransferase [Ensifer] |
|  |  | chromosome 2 | OV14_RS23990 | WP_025429137.1 | WP_025429137.1 4-hydroxybenzoate 3-monooxygenase [Ensifer adhaerens] |
|  |  | chromosome 2 | OV14_RS24045 | WP_038577932.1 | WP_038577932.1 MULTISPECIES: hypothetical protein [Ensifer] |
|  |  | chromosome 2 | OV14_RS24690 | WP_025429276.1 | WP_025429276.1 MULTISPECIES: hypothetical protein [Ensifer] |
|  |  | chromosome 2 | OV14_RS24705 | WP_025429279.1 | WP_025429279.1 glyoxalase [Ensifer adhaerens] |
|  |  | chromosome 2 | OV14_RS25365 | WP_025429405.1 | WP_025429405.1 MULTISPECIES: general stress protein [Ensifer] |
|  |  | chromosome 2 | OV14_RS25370 | WP_025429406.1 | WP_025429406.1 hypothetical protein [Ensifer adhaerens] |
|  |  | chromosome 2 | OV14_RS25910 | WP_025429504.1 | WP_025429504.1 hypothetical protein [Ensifer adhaerens] |
|  |  | chromosome 2 | OV14_RS25925 | WP_025429507.1 | WP_025429507.1 MULTISPECIES: CoA transferase [Ensifer] |
|  |  | chromosome 2 | OV14_RS26545 | WP_025429630.1 | WP_025429630.1 MULTISPECIES: iron permease [Ensifer] |
|  |  | chromosome 2 | OV14_RS27050 | WP_025429729.1 | WP_025429729.1 DNA topoisomerase [Ensifer adhaerens] |
|  |  | chromosome 2 | OV14_RS27875 | WP_025429889.1 | WP_025429889.1 MULTISPECIES: sulfatase [Ensifer] |
|  |  | chromosome 2 | OV14_RS28025 | WP_025429918.1 | WP_025429918.1 C4-dicarboxylate ABC transporter [Ensifer adhaerens] |
|  |  | chromosome 2 | OV14_RS28035 | WP_025429920.1 | WP_025429920.1 mandelate racemase [Ensifer adhaerens] |
|  |  | chromosome 2 | OV14_RS28140 | WP_025429941.1 | WP_025429941.1 ABC transporter permease [Ensifer adhaerens] |
|  |  | plasmid b | OV14_RS29375 | WP_025430167.1 | WP_025430167.1 MULTISPECIES: ribonuclease [Ensifer] |
|  |  | plasmid b | OV14_RS29700 | WP_025430231.1 | WP_025430231.1 ferrichrome ABC transporter permease [Ensifer adhaerens] |
|  |  | plasmid b | OV14_RS29825 | WP_025430255.1 | WP_025430255.1 arylsulfatase [Ensifer adhaerens] |
|  |  | plasmid b | OV14_RS29915 | WP_041693023.1 | WP_041693023.1 hypothetical protein [Ensifer adhaerens] |
|  |  | plasmid b | OV14_RS29920 | WP_025430274.1 | WP_025430274.1 hypothetical protein [Ensifer adhaerens] |
|  |  | plasmid b | OV14_RS29925 | WP_025430275.1 | WP_025430275.1 hypothetical protein [Ensifer adhaerens] |
|  |  | plasmid b | OV14_RS29930 | WP_025430276.1 | WP_025430276.1 hypothetical protein [Ensifer adhaerens] |
|  |  | plasmid b | OV14_RS30140 | WP_025430317.1 | WP_025430317.1 MULTISPECIES: hypothetical protein [Ensifer] |
|  |  | plasmid b | OV14_RS30825 | WP_025430450.1 | WP_025430450.1 Ku protein [Ensifer adhaerens] |
|  |  | plasmid b | OV14_RS30830 | WP_025430451.1 | WP_025430451.1 Ku protein [Ensifer adhaerens] |
|  |  | plasmid b | OV14_RS31240 | WP_051509374.1 | WP_051509374.1 Ku protein [Ensifer adhaerens] |
|  |  | plasmid b | OV14_RS31410 | WP_025430565.1 | WP_025430565.1 hypothetical protein [Ensifer adhaerens] |
|  |  | plasmid b | OV14_RS32190 | WP_025430715.1 | WP_025430715.1 ribonuclease [Ensifer adhaerens] |
|  |  | plasmid b | OV14_RS32210 | WP_025430719.1 | WP_025430719.1 hypothetical protein [Ensifer adhaerens] |
|  |  | plasmid b | OV14_RS32235 | WP_025430724.1 | WP_025430724.1 inosine-5-monophosphate dehydrogenase [Ensifer adhaerens] |
|  |  | plasmid b | OV14_RS32285 | WP_025430732.1 | WP_025430732.1 hypothetical protein [Ensifer adhaerens] |
|  |  | plasmid b | OV14_RS32340 | WP_025430743.1 | WP_025430743.1 hypothetical protein [Ensifer adhaerens] |
|  |  | plasmid b | OV14_RS32360 | WP_025430747.1 | WP_025430747.1 hypothetical protein [Ensifer adhaerens] |
|  |  | plasmid b | OV14_RS32365 | WP_025430748.1 | WP_025430748.1 hypothetical protein [Ensifer adhaerens] |
|  |  | plasmid b | OV14_RS32800 | WP_025430830.1 | WP_025430830.1 hypothetical protein [Ensifer adhaerens] |
| D2(BR) D5(BR) D7(BR) | 1 | chromosome 1 | OV14_RS11550 | WP_025426746.1 | WP_025426746.1 MULTISPECIES: aspartate aminotransferase family protein [Ensifer] |
| D2(BR) D3(BR) D7(BR) | 7 | chromosome 1 | OV14_RS02150 | WP_025424946.1 | WP_025424946.1 MULTISPECIES: branched-chain alpha-keto acid dehydrogenase subunit E2 [Ensifer] |
|  |  | chromosome 1 | OV14_RS07345 | WP_025425940.1 | WP_025425940.1 MULTISPECIES: flagellar hook protein FlgK [Ensifer] |
|  |  | chromosome 1 | OV14_RS07460 | WP_025425963.1 | WP_025425963.1 MULTISPECIES: alpha-glucosidase [Ensifer] |
|  |  | chromosome 2 | OV14_RS20440 | WP_025428458.1 | WP_025428458.1 MULTISPECIES: isovaleryl-CoA dehydrogenase [Ensifer] |
|  |  | chromosome 2 | OV14_RS22090 | WP_038578230.1 | WP_038578230.1 MULTISPECIES: amino acid dehydrogenase [Ensifer] |
|  |  | chromosome 2 | OV14_RS23580 | WP_025429056.1 | WP_025429056.1 MULTISPECIES: methylmalonyl-CoA carboxyltransferase [Ensifer] |
|  |  | plasmid b | OV14_RS32280 | WP_025430731.1 | WP_025430731.1 LpqC, poly [Ensifer adhaerens] |
| D2(BR) D3(BR) D5(BR) | 13 | chromosome 1 | ssrS | RF00013 | Pseudogene |
|  |  | chromosome 1 | OV14_RS04355 | WP_025425367.1 | WP_025425367.1 MULTISPECIES: hypothetical protein [Ensifer] |
|  |  | chromosome 1 | OV14_RS04495 | WP_025425389.1 | WP_025425389.1 MULTISPECIES: pilus assembly protein [Ensifer] |
|  |  | chromosome 1 | OV14_RS05800 | WP_025425641.1 | WP_025425641.1 MULTISPECIES: myo-inosose-2 dehydratase [Ensifer] |
|  |  | chromosome 1 | OV14_RS10815 | WP_007626161.1 | WP_007626161.1 MULTISPECIES: 50S ribosomal protein L33 [Rhizobiales] |
|  |  | chromosome 1 | OV14_RS12130 | WP_025426860.1 | WP_025426860.1 MULTISPECIES: hypothetical protein [Ensifer] |
|  |  | chromosome 1 | OV14_RS15280 | WP_025427466.1 | WP_025427466.1 MULTISPECIES: hypothetical protein [Ensifer] |
|  |  | chromosome 1 | ssrA | RF01849 | Pseudogene |
|  |  | chromosome 1 | OV14_RS16530 | WP_038576252.1 | WP_025430452.1 ATP-dependent DNA ligase [Ensifer adhaerens] |
|  |  | chromosome 1 | OV14_RS18220 | WP_051509215.1 | WP_051509215.1 MULTISPECIES: hypothetical protein [Ensifer] |
|  |  | chromosome 1 | OV14_RS18550 | WP_025428097.1 | WP_025428097.1 MULTISPECIES: hypothetical protein [Ensifer] |
|  |  | plasmid b | OV14_RS36590 | WP_051509329.1 | WP_051509329.1 MULTISPECIES: hypothetical protein [Ensifer] |
|  |  | plasmid b | OV14_RS30835 | WP_025430452.1 | WP_038576252.1 hypothetical protein [Ensifer adhaerens] |
| D1(BR) D5(BR) D7(BR) | 2 | chromosome 2 | OV14_RS20290 | WP_025428428.1 | WP_025428428.1 MULTISPECIES: nodulation protein NfeD [Ensifer] |
|  |  | chromosome 2 | OV14_RS24505 | WP_025429239.1 | WP_025429239.1 MULTISPECIES: arabinose ABC transporter substrate-binding protein [Ensifer] |
| D1(BR) D2(BR) D3(BR) | 1 | chromosome 2 | OV14_RS19500 | WP_025428278.1 | WP_025428278.1 Pyoverdin chromophore biosynthetic protein pvcC [Ensifer adhaerens] |
| D2(BR) D3(BR) D5(BR) D7(BR) | 26 | chromosome 1 | OV14_RS00210 | WP_025424576.1 | WP_025424576.1 hypothetical protein [Ensifer adhaerens] |
|  |  | chromosome 1 | OV14_RS36800 | WP_065784874.1 | WP_065784874.1 MULTISPECIES: DUF1127 domain-containing protein [Ensifer] |
|  |  | chromosome 1 | OV14_RS05805 | WP_025425642.1 | WP_025425642.1 MULTISPECIES: 3D-(3,5/4)-trihydroxycyclohexane-1,2-dione acylhydrolase (decyclizing) [Ensifer] |
|  |  | chromosome 1 | OV14_RS07445 | WP_025425960.1 | WP_025425960.1 MULTISPECIES: alpha-glucoside ABC transporter substrate-binding protein [Ensifer] |
|  |  | chromosome 1 | OV14_RS07450 | WP_025425961.1 | WP_025425961.1 MULTISPECIES: alpha-glucoside ABC transporter permease [Ensifer] |
|  |  | chromosome 1 | OV14_RS07455 | WP_025425962.1 | WP_025425962.1 MULTISPECIES: alpha-glucoside ABC transporter permease [Ensifer] |
|  |  | chromosome 1 | OV14_RS36805 | WP_025426013.1 | WP_025426013.1 DUF1328 domain-containing protein [Ensifer adhaerens] |
|  |  | chromosome 1 | OV14_RS07750 | WP_025426020.1 | WP_025426020.1 MULTISPECIES: DUF1508 domain-containing protein [Ensifer] |
|  |  | chromosome 1 | OV14_RS11350 | WP_025426706.1 | WP_025426706.1 MULTISPECIES: hypothetical protein [Ensifer] |
|  |  | chromosome 1 | OV14_RS11675 | WP_025426771.1 | WP_025426771.1 MULTISPECIES: membrane protein [Ensifer] |
|  |  | chromosome 1 | OV14_RS36820 | WP_067698887.1 | WP_067698887.1 DUF1127 domain-containing protein [Ensifer adhaerens] |
|  |  | chromosome 1 | OV14_RS12360 | WP_025426902.1 | WP_025426902.1 hypothetical protein [Ensifer adhaerens] |
|  |  | chromosome 1 | OV14_RS14955 | WP_025427400.1 | WP_025427400.1 MULTISPECIES: hypothetical protein [Ensifer] |
|  |  | chromosome 1 | OV14_RS15275 | WP_025427465.1 | WP_025427465.1 hypothetical protein [Ensifer adhaerens] |
|  |  | chromosome 1 | OV14_RS36825 | WP_025427496.1 | WP_025427496.1 DUF1328 domain-containing protein [Ensifer adhaerens] |
|  |  | chromosome 1 | OV14_RS18455 | WP_025428079.1 | WP_025428079.1 hypothetical protein [Ensifer adhaerens] |
|  |  | chromosome 2 | OV14_RS21455 | WP_025428654.1 | WP_025428654.1 MULTISPECIES: ABC transporter substrate-binding protein [Ensifer] |
|  |  | chromosome 2 | OV14_RS21995 | WP_025428759.1 | WP_025428759.1 MULTISPECIES: hypothetical protein [Ensifer] |
|  |  | chromosome 2 | OV14_RS22685 | WP_038577888.1 | WP_038577888.1 hypothetical protein [Ensifer adhaerens] |
|  |  | chromosome 2 | OV14_RS23385 | WP_064742370.1 | WP_064742370.1 hypothetical protein [Ensifer adhaerens] |
|  |  | chromosome 2 | OV14_RS27070 | WP_025429734.1 | WP_025429734.1 MULTISPECIES: CsbD family protein [Ensifer] |
|  |  | plasmid b | OV14_RS30150 | WP_025430319.1 | WP_025430319.1 MULTISPECIES: hypothetical protein [Ensifer] |
|  |  | plasmid b | OV14_RS31245 | WP_025430531.1 | WP_025430531.1 Ku protein [Ensifer adhaerens] |
|  |  | plasmid b | OV14_RS32240 | WP_025430725.1 | WP_025430725.1 hypothetical protein [Ensifer adhaerens] |
|  |  | plasmid b | OV14_RS32265 | WP_041692939.1 | WP_041692939.1 hypothetical protein [Ensifer adhaerens] |
|  |  | plasmid b | OV14_RS34655 | WP_025431172.1 | WP_025431172.1 hypothetical protein [Ensifer adhaerens] |
| D1(BR) D3(BR) D5(BR) D7(BR) | 1 | chromosome 2 | OV14_RS21855 | WP_025428731.1 | WP_025428731.1 MULTISPECIES: membrane protein [Ensifer] |
| D1(BR) D2(BR) D3(BR) D5(BR) | 2 | plasmid b | OV14_RS31405 | WP_025430564.1 | WP_025430564.1 MULTISPECIES: hypothetical protein [Ensifer] |
|  |  | chromosome 1 | putA | WP_025425760.1 | WP_025425760.1 MULTISPECIES: bifunctional proline dehydrogenase/L-glutamate gamma-semialdehyde dehydrogenase [Ensifer] |
| D1(BR) D2(BR) D3(BR) D5(BR) D7(BR) | 9 | chromosome 1 | OV14_RS00280 | WP_025424588.1 | WP_025424588.1 phage capsid protein [Ensifer adhaerens] |
|  |  | chromosome 1 | OV14_RS00295 | WP_038576400.1 | WP_038576400.1 terminase [Ensifer adhaerens] |
|  |  | chromosome 1 | OV14_RS05810 | WP_025425643.1 | WP_025425643.1 MULTISPECIES: 5-dehydro-2-deoxygluconokinase [Ensifer] |
|  |  | chromosome 1 | OV14_RS05820 | WP_025425645.1 | WP_025425645.1 MULTISPECIES: 1-carboxy-3-chloro-3,4-dihydroxycyclo hexa-1,5-diene dehydrogenase [Ensifer] |
|  |  | chromosome 1 | OV14_RS14055 | WP_025427221.1 | WP_025427221.1 phytanoyl-CoA dioxygenase [Ensifer adhaerens] |
|  |  | chromosome 1 | OV14_RS18300 | WP_025428049.1 | WP_025428049.1 MULTISPECIES: inositol 2-dehydrogenase [Ensifer] |
|  |  | chromosome 2 | OV14_RS23300 | WP_025429003.1 | WP_025429003.1 rhizopine-binding protein [Ensifer adhaerens] |
|  |  | chromosome 2 | OV14_RS23305 | WP_025429004.1 | WP_025429004.1 D-ribose transporter ATP-binding protein [Ensifer adhaerens] |
|  |  | chromosome 2 | OV14_RS23310 | WP_025429005.1 | WP_025429005.1 ABC transporter [Ensifer adhaerens] |

**Supplementary Table S3.**  **a**. Supporting FPKM values for 29 genes (25 *vir* genes plus, npt, aadA1, GUSPlus and hptII) from plasmid pCAMBIA5105 within EOV14_5105 across 6 timepoints (D0, D1, D2, D3, D5 and D7). **b.** Corresponding heatmap indicating presence (yes) / absence (no) of significance (*P*<0.05) between transition days D0 - D1, D1 - D2, D2 - D3, D3 - D5 and D5 - D7.

| **a.**   \| **Gene** \| **D 0** \| **D 1** \| **D 2** \| **D 3** \| **D 5** \| **D 7** \| \| --- \| --- \| --- \| --- \| --- \| --- \| --- \| \| **virK** \| 21075.7 \| 6068.69 \| 4780.93 \| 5318.24 \| 5821.16 \| 7341.17 \| \| **virA** \| 1734.24 \| 3479.11 \| 1874.84 \| 1277.67 \| 1025.96 \| 980.597 \| \| **virJ** \| 1625.63 \| 30598.6 \| 22786.7 \| 19298.8 \| 14607.5 \| 9821.18 \| \| **virB1** \| 632.34 \| 75692.5 \| 53939.5 \| 43196.1 \| 28316.5 \| 20021 \| \| **virB2** \| 1156.27 \| 51638.7 \| 41775.3 \| 28040.4 \| 16290.2 \| 9617.24 \| \| **virB3** \| 700.701 \| 38218.3 \| 36764.6 \| 25041.7 \| 16255 \| 9257.26 \| \| **virB4** \| 636.797 \| 19333.3 \| 16692.7 \| 12203.6 \| 7717.51 \| 4550.75 \| \| **virB5** \| 1043.34 \| 39300.3 \| 36108.9 \| 29936.2 \| 17940.7 \| 11727.2 \| \| **virB6** \| 462.414 \| 10276 \| 12350.8 \| 8630.1 \| 5684.08 \| 3051.87 \| \| **virB7** \| 1571.85 \| 9359.02 \| 15390.5 \| 19319.8 \| 11409.8 \| 8529.38 \| \| **virB8** \| 742.626 \| 9894.39 \| 10027.8 \| 7820.45 \| 5126.43 \| 3321.54 \| \| **virB9** \| 643.206 \| 10507 \| 10105.7 \| 7291.46 \| 4641.27 \| 2464.49 \| \| **virB10** \| 1056.69 \| 10418.6 \| 10726.8 \| 8183.21 \| 5424.21 \| 3413.23 \| \| **virB11** \| 1280.2 \| 6259.13 \| 6246.02 \| 4612.8 \| 3061.87 \| 2113.48 \| \| **virG** \| 2545.37 \| 29973.1 \| 16790.9 \| 13229.5 \| 9646.71 \| 7731.46 \| \| **virC2** \| 607.143 \| 4107.62 \| 3196.12 \| 2235.93 \| 1592.82 \| 1156.13 \| \| **virC1** \| 659.951 \| 3426.37 \| 1971.42 \| 1714.56 \| 1213.49 \| 1063.58 \| \| **virD1** \| 326.85 \| 34382.8 \| 19922 \| 13653.4 \| 10036.1 \| 6275.48 \| \| **virD2** \| 769.296 \| 15871.8 \| 11740.7 \| 8504.27 \| 5992.73 \| 3546.15 \| \| **virD3** \| 560.959 \| 7713.39 \| 5894.67 \| 4239.15 \| 2789.39 \| 1677.5 \| \| **virD4** \| 1487.67 \| 7007.1 \| 6008.07 \| 5406.74 \| 4017.43 \| 2869.14 \| \| **virD5** \| 1229 \| 4281.94 \| 3672.42 \| 3545.6 \| 2683.59 \| 1911.96 \| \| **virE1** \| 2618.3 \| 29915.2 \| 53424 \| 66163 \| 53147 \| 42126.3 \| \| **virE2** \| 1247.3 \| 29279.6 \| 36260 \| 28006 \| 19704 \| 10654.4 \| \| **virE3** \| 1388.14 \| 16676.9 \| 21231.1 \| 17737.1 \| 13636.6 \| 9067.97 \| \| **npt** \| 26903.5 \| 15252.2 \| 13353.8 \| 12062.2 \| 12304.1 \| 11099.8 \| \| **aadA1** \| 43332.6 \| 22895.8 \| 21831.5 \| 23343.9 \| 25362.4 \| 27368.3 \| \| **GUSPlus** \| 4742.72 \| 2570.69 \| 2758.5 \| 3263.57 \| 3477.93 \| 3046.36 \| \| **hptII** \| 5997.4 \| 3924.08 \| 3473.92 \| 3111.02 \| 3059.57 \| 2432.38 \| | **b.**   \| **Gene** \| **D 0-1** \| **D 1-2** \| **D 2-3** \| **D 3-5** \| **D 5-7** \| \| --- \| --- \| --- \| --- \| --- \| --- \| \| **P<0.05** \| **P<0.05** \| **P<0.05** \| **P<0.05** \| **P<0.05** \| \| **virK** \| yes \| no \| no \| no \| no \| \| **virA** \| yes \| yes \| no \| no \| no \| \| **virJ** \| yes \| no \| no \| no \| no \| \| **virB1** \| yes \| no \| no \| no \| no \| \| **virB2** \| yes \| no \| no \| no \| no \| \| **virB3** \| no \| no \| no \| no \| no \| \| **virB4** \| yes \| no \| no \| no \| no \| \| **virB5** \| yes \| no \| no \| no \| no \| \| **virB6** \| yes \| no \| no \| no \| yes \| \| **virB7** \| no \| no \| no \| no \| no \| \| **virB8** \| yes \| no \| no \| no \| no \| \| **virB9** \| yes \| no \| no \| no \| no \| \| **virB10** \| yes \| no \| no \| no \| no \| \| **virB11** \| yes \| no \| no \| no \| no \| \| **virG** \| yes \| yes \| no \| no \| no \| \| **virC2** \| yes \| no \| no \| no \| no \| \| **virC1** \| yes \| no \| no \| no \| no \| \| **virD1** \| yes \| no \| no \| no \| no \| \| **virD2** \| yes \| no \| no \| no \| no \| \| **virD3** \| no \| no \| no \| no \| no \| \| **virD4** \| yes \| no \| no \| no \| no \| \| **virD5** \| yes \| no \| no \| no \| no \| \| **virE1** \| yes \| no \| no \| no \| no \| \| **virE2** \| yes \| no \| no \| no \| yes \| \| **virE3** \| yes \| no \| no \| no \| no \| \| **npt** \| no \| no \| no \| no \| no \| \| **aadA1** \| no \| no \| no \| no \| no \| \| **GUSPlus** \| no \| no \| no \| no \| no \| \| **hptII** \| no \| no \| no \| no \| no \| |
| --- | --- | --- | --- | --- | --- | --- | --- | --- | --- | --- | --- | --- | --- | --- | --- | --- | --- | --- | --- | --- | --- | --- | --- | --- | --- | --- | --- | --- | --- | --- | --- | --- | --- | --- | --- | --- | --- | --- | --- | --- | --- | --- | --- | --- | --- | --- | --- | --- | --- | --- | --- | --- | --- | --- | --- | --- | --- | --- | --- | --- | --- | --- | --- | --- | --- | --- | --- | --- | --- | --- | --- | --- | --- | --- | --- | --- | --- | --- | --- | --- | --- | --- | --- | --- | --- | --- | --- | --- | --- | --- | --- | --- | --- | --- | --- | --- | --- | --- | --- | --- | --- | --- | --- | --- | --- | --- | --- | --- | --- | --- | --- | --- | --- | --- | --- | --- | --- | --- | --- | --- | --- | --- | --- | --- | --- | --- | --- | --- | --- | --- | --- | --- | --- | --- | --- | --- | --- | --- | --- | --- | --- | --- | --- | --- | --- | --- | --- | --- | --- | --- | --- | --- | --- | --- | --- | --- | --- | --- | --- | --- | --- | --- | --- | --- | --- | --- | --- | --- | --- | --- | --- | --- | --- | --- | --- | --- | --- | --- | --- | --- | --- | --- | --- | --- | --- | --- | --- | --- | --- | --- | --- | --- | --- | --- | --- | --- | --- | --- | --- | --- | --- | --- | --- | --- | --- | --- | --- | --- | --- | --- | --- | --- | --- | --- | --- | --- | --- | --- | --- | --- | --- | --- | --- | --- | --- | --- | --- | --- | --- | --- | --- | --- | --- | --- | --- | --- | --- | --- | --- | --- | --- | --- | --- | --- | --- | --- | --- | --- | --- | --- | --- | --- | --- | --- | --- | --- | --- | --- | --- | --- | --- | --- | --- | --- | --- | --- | --- | --- | --- | --- | --- | --- | --- | --- | --- | --- | --- | --- | --- | --- | --- | --- | --- | --- | --- | --- | --- | --- | --- | --- | --- | --- | --- | --- | --- | --- | --- | --- | --- | --- | --- | --- | --- | --- | --- | --- | --- | --- | --- | --- | --- | --- | --- | --- | --- | --- | --- | --- | --- | --- | --- | --- | --- | --- | --- | --- | --- | --- | --- | --- | --- | --- | --- | --- | --- | --- | --- | --- | --- | --- | --- | --- | --- | --- | --- | --- | --- | --- | --- | --- | --- | --- | --- | --- | --- | --- | --- | --- | --- | --- | --- | --- | --- | --- | --- | --- | --- | --- | --- | --- | --- | --- | --- | --- | --- | --- | --- | --- | --- | --- | --- | --- | --- | --- | --- | --- | --- | --- | --- | --- | --- | --- | --- | --- | --- | --- |

**Supplementary Table S4. a.** Supporting FPKM values for 29 genes (25 *vir* genes plus, npt, aadA1, GUSPlus and hptII) from plasmid pCAMBIA5105 within EOV14_5105 across 6 timepoints co-cultivated with *A. thaliana* roots across 6 timepoints (D0, D1, D2, D3, D5 and D7). **b.** Corresponding heatmap indicating presence (yes) / absence (no) of significance (*P*<0.05) between transition days D0 - D1, D1 - D2, D2 - D3, D3 - D5 and D5 - D7.

| **a.**   \| **Gene** \| **D 0** \| **D 1** \| **D 2** \| **D 3** \| **D 5** \| **D 7** \| \| --- \| --- \| --- \| --- \| --- \| --- \| --- \| \| **virK** \| 15198.5 \| 3461.86 \| 3821.02 \| 3665.29 \| 3697.38 \| 4152.57 \| \| **virA** \| 1674.53 \| 2507.19 \| 1157.9 \| 870.458 \| 705.079 \| 725.221 \| \| **virJ** \| 1510.5 \| 22666.1 \| 17970.6 \| 17548.6 \| 15227.7 \| 14016.5 \| \| **virB1** \| 640.938 \| 44349.9 \| 45249.1 \| 44221.7 \| 35224.8 \| 31454.4 \| \| **virB2** \| 1467.96 \| 36458.9 \| 34572.7 \| 34151.4 \| 25569.9 \| 17018 \| \| **virB3** \| 649.958 \| 33080.4 \| 31099.7 \| 27962.6 \| 22923.8 \| 15203.9 \| \| **virB4** \| 542.31 \| 15637 \| 14321.8 \| 12495 \| 9917.69 \| 7950.91 \| \| **virB5** \| 1088.87 \| 29293.6 \| 28988.8 \| 27250 \| 18983.9 \| 18040.8 \| \| **virB6** \| 428.811 \| 11848.4 \| 9983.69 \| 8813.48 \| 7206.84 \| 5329.76 \| \| **virB7** \| 1181.02 \| 9500.29 \| 7652.66 \| 7604.2 \| 6074.25 \| 8298.36 \| \| **virB8** \| 708.446 \| 10922.2 \| 8714.71 \| 7439.43 \| 6018.29 \| 4648.88 \| \| **virB9** \| 637.103 \| 11674.5 \| 8614.21 \| 7544.44 \| 6057.48 \| 4654.19 \| \| **virB10** \| 962.358 \| 10465 \| 9118.64 \| 8157.22 \| 6571.4 \| 5565.12 \| \| **virB11** \| 1208.89 \| 6330.64 \| 5262.27 \| 4778.87 \| 3769.9 \| 3228.68 \| \| **virG** \| 3123.96 \| 17863.2 \| 13830.1 \| 12320.9 \| 9881.07 \| 10455.1 \| \| **virC2** \| 730.35 \| 3523.04 \| 2580.45 \| 2371.83 \| 1938.82 \| 1492.22 \| \| **virC1** \| 424.291 \| 2453.03 \| 1497.55 \| 1366.45 \| 1107.98 \| 981.09 \| \| **virD1** \| 372.468 \| 23618.6 \| 15957.5 \| 15488.9 \| 13787.4 \| 10976.2 \| \| **virD2** \| 648.153 \| 12719.7 \| 8951.34 \| 8083.4 \| 7411.76 \| 5685.6 \| \| **virD3** \| 398.404 \| 5802.18 \| 4216.65 \| 3923.39 \| 3534.24 \| 2436.56 \| \| **virD4** \| 1075.29 \| 5496.48 \| 4748.07 \| 4596.43 \| 3912.72 \| 3630.02 \| \| **virD5** \| 985.687 \| 3079.34 \| 2985.52 \| 3090.54 \| 2708.49 \| 2646.38 \| \| **virE1** \| 3829.04 \| 17886.2 \| 28250.6 \| 37457.1 \| 35280.9 \| 42590.5 \| \| **virE2** \| 1356.13 \| 20469.4 \| 27123.6 \| 31446.3 \| 32428.4 \| 21844.3 \| \| **virE3** \| 1475.31 \| 11780.9 \| 16067.2 \| 18231.3 \| 18987.6 \| 14472.8 \| \| **npt** \| 27979 \| 11431.7 \| 12373.8 \| 14391.7 \| 16234.6 \| 12875 \| \| **aadA1** \| 41277.2 \| 15549.2 \| 17894.7 \| 19825.1 \| 21252.3 \| 25613.8 \| \| **GUSPlus** \| 4298.69 \| 1863.42 \| 2099.71 \| 2443.42 \| 2618.59 \| 3518.45 \| \| **hptII** \| 6111.51 \| 3513.41 \| 3211.41 \| 3370.68 \| 3115.47 \| 3325.72 \| | **b.**   \| **Gene** \| **D 0-1** \| **D 1-2** \| **D 2-3** \| **D 3-5** \| **D 5-7** \| \| --- \| --- \| --- \| --- \| --- \| --- \| \| **P<0.05** \| **P<0.05** \| **P<0.05** \| **P<0.05** \| **P<0.05** \| \| **virK** \| yes \| no \| no \| no \| no \| \| **virA** \| yes \| yes \| no \| no \| no \| \| **virJ** \| yes \| no \| no \| no \| no \| \| **virB1** \| yes \| no \| no \| no \| no \| \| **virB2** \| yes \| no \| no \| no \| no \| \| **virB3** \| no \| no \| no \| no \| no \| \| **virB4** \| yes \| no \| no \| no \| no \| \| **virB5** \| yes \| no \| no \| no \| no \| \| **virB6** \| yes \| no \| no \| no \| no \| \| **virB7** \| no \| no \| no \| no \| no \| \| **virB8** \| yes \| no \| no \| no \| no \| \| **virB9** \| yes \| no \| no \| no \| no \| \| **virB10** \| yes \| no \| no \| no \| no \| \| **virB11** \| yes \| no \| no \| no \| no \| \| **virG** \| yes \| no \| no \| no \| no \| \| **virC2** \| yes \| no \| no \| no \| no \| \| **virC1** \| yes \| yes \| no \| no \| no \| \| **virD1** \| yes \| no \| no \| no \| no \| \| **virD2** \| yes \| no \| no \| no \| no \| \| **virD3** \| no \| no \| no \| no \| no \| \| **virD4** \| yes \| no \| no \| no \| no \| \| **virD5** \| yes \| no \| no \| no \| no \| \| **virE1** \| yes \| no \| no \| no \| no \| \| **virE2** \| yes \| no \| no \| no \| no \| \| **virE3** \| yes \| no \| no \| no \| no \| \| **npt** \| no \| no \| no \| no \| no \| \| **aadA1** \| yes \| no \| no \| no \| no \| \| **GUSPlus** \| no \| no \| no \| no \| no \| \| **hptII** \| no \| no \| no \| no \| no \| |
| --- | --- | --- | --- | --- | --- | --- | --- | --- | --- | --- | --- | --- | --- | --- | --- | --- | --- | --- | --- | --- | --- | --- | --- | --- | --- | --- | --- | --- | --- | --- | --- | --- | --- | --- | --- | --- | --- | --- | --- | --- | --- | --- | --- | --- | --- | --- | --- | --- | --- | --- | --- | --- | --- | --- | --- | --- | --- | --- | --- | --- | --- | --- | --- | --- | --- | --- | --- | --- | --- | --- | --- | --- | --- | --- | --- | --- | --- | --- | --- | --- | --- | --- | --- | --- | --- | --- | --- | --- | --- | --- | --- | --- | --- | --- | --- | --- | --- | --- | --- | --- | --- | --- | --- | --- | --- | --- | --- | --- | --- | --- | --- | --- | --- | --- | --- | --- | --- | --- | --- | --- | --- | --- | --- | --- | --- | --- | --- | --- | --- | --- | --- | --- | --- | --- | --- | --- | --- | --- | --- | --- | --- | --- | --- | --- | --- | --- | --- | --- | --- | --- | --- | --- | --- | --- | --- | --- | --- | --- | --- | --- | --- | --- | --- | --- | --- | --- | --- | --- | --- | --- | --- | --- | --- | --- | --- | --- | --- | --- | --- | --- | --- | --- | --- | --- | --- | --- | --- | --- | --- | --- | --- | --- | --- | --- | --- | --- | --- | --- | --- | --- | --- | --- | --- | --- | --- | --- | --- | --- | --- | --- | --- | --- | --- | --- | --- | --- | --- | --- | --- | --- | --- | --- | --- | --- | --- | --- | --- | --- | --- | --- | --- | --- | --- | --- | --- | --- | --- | --- | --- | --- | --- | --- | --- | --- | --- | --- | --- | --- | --- | --- | --- | --- | --- | --- | --- | --- | --- | --- | --- | --- | --- | --- | --- | --- | --- | --- | --- | --- | --- | --- | --- | --- | --- | --- | --- | --- | --- | --- | --- | --- | --- | --- | --- | --- | --- | --- | --- | --- | --- | --- | --- | --- | --- | --- | --- | --- | --- | --- | --- | --- | --- | --- | --- | --- | --- | --- | --- | --- | --- | --- | --- | --- | --- | --- | --- | --- | --- | --- | --- | --- | --- | --- | --- | --- | --- | --- | --- | --- | --- | --- | --- | --- | --- | --- | --- | --- | --- | --- | --- | --- | --- | --- | --- | --- | --- | --- | --- | --- | --- | --- | --- | --- | --- | --- | --- | --- | --- | --- | --- | --- | --- | --- | --- | --- | --- | --- | --- | --- | --- | --- | --- | --- | --- | --- | --- | --- | --- | --- | --- | --- | --- | --- | --- | --- | --- | --- | --- | --- | --- | --- | --- | --- | --- | --- | --- | --- |

**Supplementary Table S5.** Functional categories from KEGG database assigned to protein IDs from *E. adhaerens* OV14 obtained using BlastKOALA with a cutoff E-value of 0.00001

| **KEGG functional categories** | **KO identifier** | **Gene name** | **Function** | **Protein ID** | **Gene ID** |
| --- | --- | --- | --- | --- | --- |
| - **Environmental Information Processing (EIP)** |  |  |  |  |  |
| Membrane Transporters (MT) |  |  |  |  |  |
| *ABC transporters (ABC-t)* | K01996 | *livF* | Quorum sensing | WP_025424980.1 | OV14_RS02350 |
|  | K01997 | *livH* | Quorum sensing | WP_025426479.1 WP_025428147.1 | OV14_RS10105  OV14_RS18800 |
|  | K01998 | *livM* | Quorum sensing | WP_025428146.1 | OV14_RS18795 |
|  | K01999 | *livK* | Quorum sensing | WP_025424978.1 WP_025426192.1 WP_025428142.1 WP_025429159.1 | OV14_RS02340  OV14_RS08630  OV14_RS18775  OV14_RS24100 |
| …..*Bacterial secretion system (BSS)* | K03116 | *tatA* | Twin-arginine translocation (Tat) system  Sec-independent protein translocase protein TatA | WP_025430220.1 | OV14_RS29640 |
| - **Cellular processes** |  |  |  |  |  |
| Cellular community – prokaryotes (CCP) |  |  |  |  |  |
| *Quorum sensing (QS)* | K03666 | *hfq* | Quorum sensing  RNA degradation  Biofilm formation - Vibrio cholerae | WP_003535434.1 | OV14_RS11740 |
|  | [K10914](http://www.kegg.jp/dbget-bin/www_bget?K10914) | *crp* | Two-component system  Quorum sensing  Biofilm formation - Pseudomonas aeruginosa  Biofilm formation - Escherichia coli  Biofilm formation - Vibrio cholerae | WP_025425766.1 | OV14_RS06440 |
|  | K11749 | *rseP* | Quorum sensing | WP_025426824.1 | OV14_RS11950 |
|  | K20529 | *trbD* | Quorum sensing | WP_025430065.1 | OV14_RS28805 |
| Cell motility (CM) |  |  |  |  |  |
| *Bacterial chemotaxis (BCh)* | K02410 | *fliG* | Bacterial chemotaxis  Flagellar assembly | WP_025425910.1 | OV14_RS07185 |
|  | K02416 | *fliM* | Bacterial chemotaxis  Flagellar assembly | WP_025425912.1 | OV14_RS07195 |
|  | K02417 | *fliNY, fliN* | Bacterial chemotaxis  Flagellar assembly | WP_025425911.1 | OV14_RS07190 |
|  | K02557 | *motB* | Bacterial chemotaxis  Flagellar assembly | WP_025425934.1 | OV14_RS07315 |
|  | K03406 | *mcp* | Two-component system  Bacterial chemotaxis | WP_025425031.1 WP_025425751.1 WP_025425895.1 WP_025426142.1 WP_025426143.1 WP_025427253.1 | OV14_RS02625  OV14_RS06355  OV14_RS07110  OV14_RS08375  OV14_RS08380  OV14_RS14215 |
|  | K03407 | *cheA* | Two-component system  Bacterial chemotaxis | WP_025425898.1 | OV14_RS07125 |
|  | K03413 | *cheY* | Two-component system  Bacterial chemotaxis | WP_025425897.1 | OV14_RS07120 |
|  | K10439 | *rbsB* | ABC transporters  Bacterial chemotaxis | WP_025429274.1 WP_025429301.1 WP_025429865.1 WP_025430852.1 | OV14_RS24680  OV14_RS24820  OV14_RS27755  OV14_RS32910 |
| *Flagellar assembly (FA)* | K02388 | *flgC* | Flagellar assembly | WP_025425919.1 | OV14_RS07230 |
|  | K02390 | *flgE* | Flagellar assembly | WP_025425939.1 | OV14_RS07340 |
|  | K02391 | *flgF* | Flagellar assembly | WP_025425915.1 | OV14_RS07210 |
|  | K02392 | *flgG* | Flagellar assembly | WP_025425921.1 | OV14_RS07240 |
|  | K02396 | *flgK* | Flagellar assembly | WP_025425940.1 | OV14_RS07345 |
|  | K02397 | *flgL* | Flagellar assembly | WP_025425941.1 | OV14_RS07350 |
|  | K02401 | *flhB* | Flagellar assembly | WP_025425909.1 | OV14_RS07180 |
|  | K02406 | *fliC* | Flagellar assembly | WP_025425931.1 WP_025425932.1 | OV14_RS07290  OV14_RS07305 |
|  | K02409 | *fliF* | Flagellar assembly | WP_025425905.1 | OV14_RS07160 |
|  | K02410 | *fliG* | Flagellar assembly | WP_025425910.1 |  |
|  | K02412 | *fliI* | Flagellar assembly | WP_025425916.1 | OV14_RS07215 |
|  | K02416 | *fliM* | Bacterial chemotaxis  Flagellar assembly | WP_025425912.1 | OV14_RS07185 |
|  | K02417 | *fliNY, fliN* | Bacterial chemotaxis  Flagellar assembly | WP_025425911.1 | OV14_RS07190 |
|  | K02557 | *motB* | Bacterial chemotaxis  Flagellar assembly | WP_025425934.1 | OV14_RS07315 |
| - **Genetic Information Processing (GIP)** |  |  |  |  |  |
| Replication and repair (RR) |  |  |  |  |  |
| DNA replication (DR) | K03470 | *rnhB* | DNA replication | WP_025426103.1 | OV14_RS08180 |
| Base excision repair (BER) | K01142 | *E3.1.11.2, xthA* | Base excision repair  single strand breaks repair  base exicision repair | WP_025425159.1 WP_025430449.1 | OV14_RS03300  OV14_RS30820 |
| Mismatch repair (MR) | K03572 | *mutL* | Mismatch repai  single strand breaks repair  mismatch exicision repair | WP_025426077.1 | OV14_RS08050 |
| Homologous recombination (HR) | K03550 | *ruvA* | double strand breaks repair  homologous recombination | WP_025424742.1 | OV14_RS01085 |
| Non-homologous end-joining (NHEJ) | K01971 | *ligD* | non-homologous end-joining  double strand breaks repair | WP_025430452.1 WP_041692940.1 | OV14_RS30835  OV14_RS32320 |
|  | K10979 | *ku* | non-homologous end-joining  double strand breaks repair | WP_025430450.1 WP_025430451.1 WP_025430531.1 WP_051509374.1 | OV14_RS30825  OV14_RS30830  OV14_RS31245  OV14_RS31240 |

**Supplementary Table S6.** Differential expression of genes proposed by Rudder et al. 2015 (by homology) to be involved in transformation process. Asterisks indicate timepoints where the expression was found to be significantly different in bacteria exposed to roots

| **gene_name** | **gene_locus** | **Co-cultivation days** | **Up- or down-regulated in BR** | **log2fold change BR/B** |
| --- | --- | --- | --- | --- |
| aopB | OV14_RS10135 | D0 | Upregulated | 0.76 |
|  |  | D1 | Upregulated | 0.36 |
|  |  | D2 | Upregulated | 1.06* |
|  |  | D3 | Upregulated | 0.905* |
|  |  | D5 | Upregulated | 0.94* |
|  |  | D7 | Upregulated | 1.00* |
| trbD | OV14_RS28805 | D0 | Upregulated | 0.27 |
|  |  | D1 | Downregulated | -0.002 |
|  |  | D2 | Upregulated | 1.35 |
|  |  | D3 | Upregulated | 1.18 |
|  |  | D5 | Upregulated | 1.00* |
|  |  | D7 | Upregulated | 1.00* |
